# Everolimus in *de novo* kidney transplant recipients participating in the Eurotransplant senior program: Results of a prospective randomized multicenter study (SENATOR)

**DOI:** 10.1371/journal.pone.0222730

**Published:** 2019-09-19

**Authors:** Susanne Brakemeier, Wolfgang Arns, Frank Lehner, Oliver Witzke, Oliver Vonend, Claudia Sommerer, Anja Mühlfeld, Thomas Rath, Robert Schuhmann, Bianca Zukunft, Irena Kroeger, Martina Porstner, Klemens Budde

**Affiliations:** 1 Department of Nephrology and Medical Intensive Care, Charité Campus Mitte, Berlin, Germany; 2 Department of Nephrology and Transplantation, Cologne Merheim Medical Center, Cologne, Germany; 3 Department of General, Visceral and Transplantation Surgery, Hannover Medical School, Hannover, Germany; 4 Department of Infectious Diseases, University Duisburg-Essen, Essen, Germany; 5 Department of Nephrology, University Duisburg-Essen, Essen, Germany; 6 Department of Nephrology, University Dusseldorf, Medical Faculty, Dusseldorf, Germany; 7 Department of Nephrology, University of Heidelberg, Heidelberg, Germany; 8 Division of Nephrology and Immunology, University Hospital RWTH Aachen, Aachen, Germany; 9 Department of Medicine III, Westpfalz- Kaiserslautern, Germany; 10 Medical Department, Novartis Pharma GmbH, Nürnberg, Germany; University of Glasgow, UNITED KINGDOM

## Abstract

Early conversion to everolimus was assessed in kidney transplant recipients participating in the Eurotransplant Senior Program (ESP), a population in whom data are lacking. The SENATOR multicenter study enrolled 207 kidney transplant recipients undergoing steroid withdrawal at week 2 post-transplant (ClinicalTrials.gov [NCT00956293]). At week 7, patients were randomized (1:2 ratio) to continue the previous calcineurin inhibitor (CNI)-based regimen with mycophenolic acid (MPA) and cyclosporine or switch to a CNI-free regimen with MPA, everolimus (5–10 ng/mL) and basiliximab at weeks 7 and 12, then followed for 18 weeks to month 6 post-transplant. The primary endpoint was estimated GFR (eGFR). At week 7, 77/207 (37.2%) patients were randomized (53 everolimus, 24 control). At month 6, eGFR was comparable: 36.5±10.8ml/min with everolimus versus 42.0±13.0ml/min in the control group (p = 0.784). Discontinuation due to adverse events occurred in 27.8% of everolimus-treated patients and 0.0% of control patients (p = 0005). Efficacy profiles showed no difference. In conclusion, eGFR, safety and efficacy outcomes at month 6 post-transplant showed no difference between groups. The everolimus group experienced a higher rate of discontinuation due to adverse events. However, the high rate of non-randomization is highly relevant, indicating this to be a somewhat unstable patient population regardless of treatment.

## Introduction

The transplant population is ageing [1]. The proportion of patients transplanted within the Eurotransplant Senior Program (ESP), which allocates grafts from donors aged over 65 years to recipients older than 65 years, has risen to more than 25% of all kidney transplant (KTx) patients in Europe [2]. Presenting with a high rate of co-morbidities on one hand, and decreased innate and adaptive immunity on the other [3], immunosuppressive regimens tailored specifically for ESP transplant recipients have long been the subject of debate [4]. However, the number of randomized studies investigating immunosuppressive regimens in elderly patients is remarkably small [4–8]. The protocols of many clinical trials exclude elderly recipients, a practice that has been repeatedly criticized by the Food and Drug Administration (FDA) [9].

Older KTx recipients are at increased risk for infection and malignancy, and extended criteria donor (ECD) kidney grafts have higher rates of delayed graft function (DGF) and are more vulnerable to calcineurin-inhibitor (CNI)-induced toxicity [10]. As a consequence, despite the lack of randomized trials in this setting, CNI minimization or avoidance and steroid withdrawal have been recommended for elderly KTx recipients at low immunological risk [4, 11, 12]. However, elderly patients do reject their graft [13, 14], especially poorly-matched donor organs from elderly donors, and T-cell-mediated rejection (TCMR) affects long-term graft function. A tailored immunosuppressive strategy is therefore required for this subgroup of patients [15, 16].

In order to reduce CNI-related effects–especially CNI-induced toxicity–a number of randomized conversion studies have been performed in the general transplant population to investigate immunosuppressive regimens using mammalian target of rapamycin inhibitor (mTORi) therapy to facilitate CNI withdrawal [17–24]. Results have been partly conflicting, but indicate improved long-term renal function, slightly higher rates of rejection, and in some studies higher rates of discontinuation under mTORi-based immunosuppression compared to CNI-based regimens. In addition, steroid-free immunosuppressive regimens have been studied extensively in recent years. Steroid-free therapy is considered justified because of significant benefits for cardiovascular risk, but is limited to low-risk recipients to avoid an increased risk of acute rejection episodes [25–28].

Although repeatedly recommended for elderly patients receiving ECD kidney transplants, CNI free and/or steroid-free regimens have never been investigated in randomized trials [15, 29].

The present study is a 6-month, open-label, randomized, multicenter, prospective, controlled study undertaken with the objective of evaluating the efficacy, safety and tolerability of a steroid- and CNI-free regimen with everolimus and mycophenolic acid (MPA) under the umbrella of induction with the interleukin-2 receptor (IL-2R) antibody basiliximab compared with a standard cyclosporine (CsA)-based immunosuppressive regimen in *de novo* renal transplant recipients participating in the Eurotransplant senior program (SENATOR).

## Materials and methods

This 6-month prospective, multicenter, open-label, randomized (2:1), controlled study with two parallel treatment groups in *de novo* senior renal transplant recipients participating in the ESP compared a CNI- free immunosuppressive regimen with everolimus with a standard CsA-based CNI regimen. The primary objective was to examine the superiority of the everolimus-based treatment regimen versus the standard CNI regimen with respect to renal function at month 6 after KTx. The planned follow-up time was 5 years. The study was conducted in compliance with Good Clinical Practice and the Declaration of Helsinki and is registered at www.clinicaltrials.gov (NCT00956293) and the European Clinical Trials Database (EudraCT-NO. 2008-005109-20). The authors confirm that all ongoing and related trials for this drug/intervention are registered.

Ten German transplant centers participated in the study. Ethical commitee approval was obtained in April 2009 and health authority approval in June 2009. The first patient was enrolled in July 2009, the last patient was enrolled in March 2012 with the final observation visit in March 2013. The clinical study report was submitted in March 2014. The study, and the follow-up period, were terminated prematurely because the required sample size of 240–260 randomized patients could not be achieved within a reasonable time frame.

### Inclusion and exclusion criteria

Patients aged >65 years receiving their first kidney from a deceased donor aged >65 years in the ESP were eligible for participation. Prior to randomization at week 7, patients were to have a stable serum creatinine level of <3.0 mg/dL and proteinuria <500 mg/g creatinine, and were to be on an immunosuppressive regimen which included CsA and MPA at a minimum 720 mg/d.

Exclusion criteria were a historical or current peak panel reactive antibody (PRA) >25%, ABO-incompatible transplantation, thrombocytopenia (platelets < 75,000/mm^3^), leukopenia (leukocytes <2,500/mm^3^), or hemoglobin <6 g/dL. Exclusion criteria for randomization were rejection (Banff >1A), thrombocytopenia, leukopenia or hemoglobin <6 g/dL, or intractable immunosuppressant complications or side effects (e.g. severe gastrointestinal adverse events) at the randomization visit.

### Treatment and comedication

**Fig 1:**
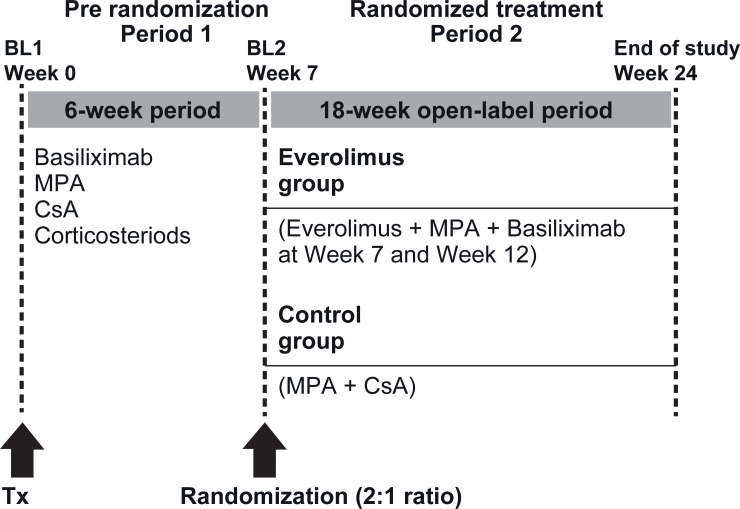
Study design. BL1, baseline visit pre-transplant; BL2, baseline visit at randomization; Tx, transplantation; MPA, mycophenolic acid; CsA cyclosporine.

For the first 6 weeks post-KTx (prior to randomization), all patients were to receive induction therapy with 2 doses (each 20 mg) of basiliximab on day 0 and day 4 post-KTx, and were to start on an immunosuppressive regimen consisting of MPA (with a loading dose of 2880 mg/day during week 1 then 2160 mg/day from week 2 to week 6) and CsA (target trough levels: week 1–4: 150–200 ng/mL, week 5–6: 100–150 ng/mL) (Fig 1). Corticosteroids were to be added to the immunosuppressive regimen according to local standard practice, but at a minimum dose of 5 mg/day. Corticosteroids were to be discontinued after week 2.

A randomization list was produced centrally using a system which automates the random assignment of treatment groups to randomization numbers in the specified 1:2 ratio (control: everolimus). Two sets of randomization numbers were prepared for stratified randomization, one set for the group of patients who did not experience any rejection between prior to week 7 and one set for the group of patients who did experience at least one rejection prior to week 7.

At randomization (week 7), patients were randomized to one of the two treatment groups in a 1:2 ratio: A) Control group: Continue the prior CNI-based regimen with MPA + CsA as administered prior to randomization, or B) Everolimus group: Stepwise switch to a steroid- and CNI-free regimen with MPA + everolimus: Starting on the day of randomization, 3 mg everolimus were to be taken on day 1 and 2 x 1.5 mg on day 2 after randomization; afterwards the everolimus dosage was to be based on blood trough level (5–10 ng/mL). Two additional basiliximab doses (each 20 mg) were given at week 7 and week 12.

The randomized treatment period was to be 18 weeks, followed by an originally planned extension period up to month 60. However, due to slow enrollment and only low sample size that could be reached, the study was prematurely terminated, hence none of the randomized patients completed the 54-month follow-up phase.

### Study endpoints and evaluation

The primary endpoint was renal function at month 6 after KTx as assessed by estimated glomerular filtration rate (eGFR, Cockcroft-Gault method). Secondary endpoints were renal function, safety and tolerability, efficacy and treatment failure at month 6. Renal function was assessed by eGFR (Modification of Diet in Renal Disease [MDRD] and Nankivell formulae) and by serum creatinine level at month 6 after KTx as well as the creatinine slope between week 7 (randomization) and month 6. Efficacy was analyzed assessing occurrence of treatment failure (a composite endpoint of biopsy-proven acute rejection, graft loss, death, loss to follow up and discontinuation due to lack of efficacy or toxicity, or conversion to another regimen) up to or at month 6.

Safety analyses included evaluation of adverse events (AE) and serious adverse events (SAE) (MedDRA version 12.1) with a special focus on infections, tumors and diabetes mellitus, and evaluation of hematology, blood chemistry, viral serology, urinanalysis, vital signs, physical condition and ECG.

### Statistical methods

Due to early termination of the study, all statistical tests were exploratory. Analysis of the primary endpoint (eGFR according to the Cockcroft-Gault method) was performed by fitting an analysis of covariance (ANCOVA) model to the data, with the primary endpoint as dependent variable and with the factor treatment and the covariate eGFR (Cockcroft-Gault method) at randomization as predictors. P values and unadjusted and adjusted (i.e., least square; LS) means of the treatment contrast with 95% confidence intervals (CIs) are presented. The full analysis set (FAS) was used for the primary analysis. Sensitivity analyses included repetition of the primary analysis based on the per protocol set (PPS), applying the last observation carried forward (LOCF) method for missing value imputation in the FAS, and using a mixed model for repeated measures (MMRM) to deal with missing values occurring in the FAS after randomization. All analysis was performed according to the intent-to-treat (ITT) principle.

Secondary efficacy variables were defined as eGFR, calculated according to the Nankivell and MDRD methods, and the observed serum creatinine value. These variables were analyzed in the same way as the primary efficacy variable. In an additional analysis, the creatinine slope (1/serum creatinine versus time) was calculated for each treatment group using a mixed model with a time × treatment group interaction.

For all events (biopsy-proven acute rejection, graft loss, death, treatment failure), Kaplan Meier estimates were tabulated and event rates were tested for differences between treatment groups by the log rank test, based on the FAS. Safety data were analyzed using summary statistics and shift tables, as appropriate.

Statistical analyses were performed using SAS version 9.2.

## Results

**Fig 2:**
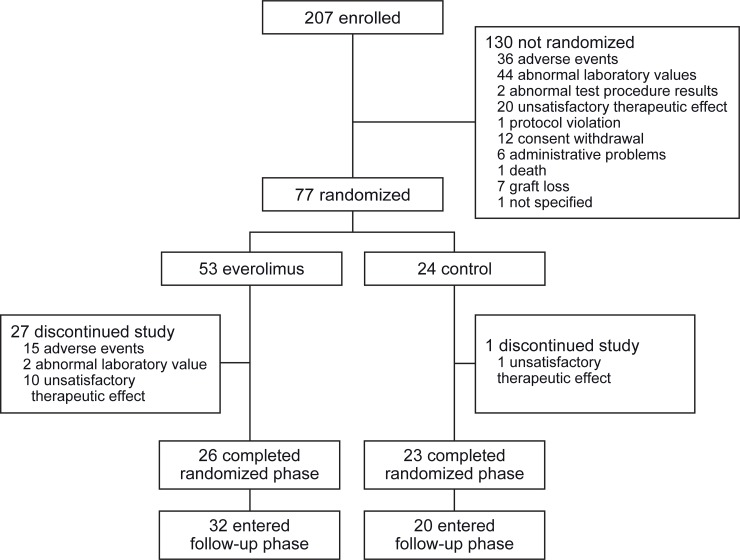
Patient disposition.

A total of 207 patients were enrolled in the study, of whom 77 were randomized at week 7 (77/207 [37.2%]; 53 in the everolimus group and 24 in the control group) (Fig 2). Since two patients were randomized to the everolimus group but not converted from CsA, the FAS consisted of 75 patients (51 patients in the everolimus group and 24 patients in the control group) (Table 1).

**Table 1:** Patient disposition (enrolled safety set).

	Control N (%)	Everolimus N (%)	Total N (%)
Enrolled			207 (100.0)
Discontinued prior to randomization			130 (62.8)
*Main reasons for discontinuation*^a^			
Abnormal laboratory value			44 (21.3)
Acute rejection			36 (17.4)
Unsatisfactory therapeutic effect			20 (9.7)
Subject withdrew consent			12 (5.8)
Graft loss			7 (3.4)
Administrative problems			6 (2.9)
Protocol violation			1 (0.5)
Death			1 (0.5)
Not specified			1 (0.5)
Randomized (PPS)	24 (100.0)	53 (100.0)	77 (100.0)
Randomized (FAS)^b^	24	51	75
Completed treatment phase^c^	23 (95.8)	26 (49.1)	49 (63.6)
Discontinued treatment phase^c^	1 (4.2)	27 (50.9)	28 (36.4)
*Main reasons for discontinuation*			
Adverse event	0 (0.0)	15 (28.3)	15 (19.5)
Unsatisfactory therapeutic effect	1 (4.2)	10 (18.9)	11 (14.3)
Abnormal laboratory value	0 (0.0)	2 (3.8)	2 (2.6)
Rejection	5 (20.8)	12 (23.5)	19 (24.7)
Included in the follow-up phase	20 (83.3)	32 (60.4)	52 (67.5)

^a^ Prior to randomization

^b^ 2 patients randomized to everolimus were not converted to everolimus therapy

^c^ From randomization to month 6 post-transplant

FAS, full analysis set; PPS, per protocol set

Patient characteristics are shown in Table 2. The proportion of patients with glomerulonephritis as the end-stage renal disease leading to KTx was nearly twice as high in the control group than in the everolimus group, but overall the two treatment groups were adequately balanced in terms of demographic and other baseline characteristics.

**Table 2:** Patient characteristics.

	Control N = 24	Everolimus N = 51	Total N = 75
Mean age ± SD, years	69.3±3.1	68.4±3.3	68.7±3.3
Male, n (%)	16 (66.7)	25 (49.0)	41 (54.7)
White, n (%)	23 (95.8)	51 (100)	74 (98.6)
Mean BMI ± SD, kg/m2	26.0±3.6	25.7±3.4	25.8±3.4
Hypertension, n (%)	14 (73.7)	30 (66.7)	44 (68.8)
Diabetes, n (%)	2 (8.3)	6 911.8)	8 (10.7)
Cytomegalovirus (IgG), n (%)	15 (62.5)	35 (68.6)	50 (66.7)
End-stage disease leading to KTx, n (%)			
Glomerulonephritis	10 (41.7)	12 (23.5)	22 (29.3)
Polycystic disease	2 (8.3)	5 (9.8)	7 (9.3)
Hypertension/nephrosclerosis	1 (4.2)	8 (15.7)	9 (12.0)
Diabetes mellitus	2 (8.3)	6 (11.8)	8 (10.7)
Other	9 (37.5)	20 (39.2)	29 (38.7)
Mean PRA ± SD, %	0.0 ± 0.0	0.3 ± 1.7	0.2 ± 1.4
HLA mismatches, n (%)			
0	1 (4.2)	0 (0.0)	1 (1.3)
1	0 (0.0)	1 (2.0)	1 (1.3)
2	1 (4.2)	5 (9.8)	6 (8.0)
3	6 (25.0)	11 (21.6)	17 (22.7)
4	7 (29.2)	11 (21.6)	18 (24.0)
5	6 (25.0)	13 (25.5)	19 (25.3)
6	3 (12.5)	10 (19.6)	13 (17.3)
Mean donor age ± SD, years	69.5 ± 7.5	72.8 ± 4.5	71.7 ± 5.8
Donor gender: male, n (%)	9 (37.5)	17 (33.3)	26 (34.7)
Donor hypertension, n (%)	14/19^a^ (73.7)	30/45^a^ (66.7)	44/64^a^ (68.8)

^a^ Data on hypertension not available for all donors

BMI, body mass index; CMV, cytomegalovirus; KTx, kidney transplantation; PRA, panel reactive antibodies; HLA, human leukocate antigen; SD, standard deviation.

### Pre-randomization phase

In total, 130 patients (130/207, 62.8%) discontinued the study before randomization. The most frequent reasons were abnormal laboratory values (44/207, 21.3%) and AEs (36/207, 17.4%). The most common AEs reported prior to randomization are shown in Table 3. The incidence of SAEs was higher in non-randomized patients compared to randomized patients up to the point of randomization (65.9% versus 39.6%) (Table 4). One patient died from multi-organ failure prior to randomization, and eight grafts were lost (Table 1).

**Table 3:** Most frequent adverse events (AEs) prior to randomization (enrolled safety set).

	Randomized (N = 77), n (%)	Not randomized (N = 126), n (%)
Number (%) of patients with AEs	77 (100.0)	126 (100.0)
*Injury*, *poisoning and procedural complications*	56 (72.7)	106 (84.1)
Wound complications	23 (29.9)	37 (29.4)
Procedural pain	14 (18.2)	40 (31.7)
Complications of transplanted kidney	10 (13.0)	35 (27.8)
*Infections and infestations*	51 (66.2)	89 (70.6)
Urinary tract infection	35 (45.5)	62 (49.2)
*Gastrointestinal disorders*	49 (63.6)	90 (71.4)
Constipation	30 (39.0)	49 (38.9)
Diarrhea	6 (7.8)	30 (23.8)
Nausea	25 (32.5)	35 (27.8)
Vomiting	8 (10.4)	30 (23.8)
*Blood and lymphatic system disorders*	40 (51.9)	65 (51.6)
Anemia	15 (19.5)	29 (23.0)
*Vascular disorders*	39 (50.6)	82 (65.1)
Hypertension	21 (27.3)	41 (32.5)
Hypotension	11 (14.3)	27 (21.4)
*Investigations*	26 (33.8)	65 (51.6)
Blood creatinine increased	18 (23.4)	47 (37.3)
*Renal and urinary tract disorders*	36 (46.8)	74 (58.7)
Hematuria	16 (20.8)	29 (23.0)
Proteinuria	9 (11.7)	26 (20.6)
*Metabolism and nutrition disorders*	63 (81.8)	97 (77.0)
Hypokalemia	30 (39.0)	29 (23.0)
Hyperkalemia	17 (22.1)	33 (26.2)
Hypocalcemia	16 (20.8)	21 (16.7)
*Psychiatric disorders*	25 (32.5)	47 (37.3)
*Cardiac disorders*	17 (22.1)	30 (23.8)
*Musculoskeletal disorders*	13 (16.9)	25 (19.8)
*Respiratory*, *thoracic and mediastinal disorders*	12 (15.6)	26 (20.6)
*Nervous system disorder*	7 (9.1)	17 (13.5)
*Endocrine disorders*	6 (7.8)	2 (1.6)
*Skin disorders*	6 (7.8)	6 (4.8)
*Eye disorders*	4 (5.2)	2 (1.6)

**Table 4:** Number of patients with adverse events.

	Control n (%)	Everolimus n (%)	Not randomized n (%)	Total n (%)
**Prior to randomization**	N = 24	N = 53	N = 126	N = 203
AEs	24 (100.0)	53 (100.0)	126 (100.0)	203 (100.0)
SAEs	11 (45.8)	21 (39.6)	83 (65.9)	115 (56.7)
Deaths	0 (0.0)	0 (0.0)	1 (0.8)	1 (0.5)
Graft loss	0 (0.0)	0 (0.0)	8 (6.3)	8 (3.9)
**Randomization to month 6**	N = 24	N = 51		N = 75
AEs	21 (87.5)	50 (98.0)	-	71 (94.7)
SAEs	11 (45.8)	28 (54.9)	-	39 (52.0)
Deaths	0 (0.0)	0 (0.0)	-	0 (0.0)
Graft loss	0 (0.0)	0 (0.0)	--	0 (0.0)
Diabetes	0 (0.0)	1 (2.0)	-	1 (1.3)
Malignancy	0 (0.0)	1 (2.0)	-	1 (1.3)
AEs causing permanent study drug discontinuation	0 (0.0)	16 (30.2)	-	16 (7.9)
AEs requiring study drug dose adjustment/interruption	0 (0.0)	9 (17.0)	-	9 (4.4)
AEs requiring significant additional therapy	17 (70.8)	49 (92.5)	-	66 (32.5)
**Follow-up phase**	N = 20^a^	N = 32^a^	-	N = 52^a^
Malignancies	2 (10.0)	2 (6.3)	-	4 (7.7)
Serious infections	5 (25.0)	13 (40.6)	-	18 (34.6)
Diabetes	1 (5.0)	1 (3.1)	-	2 (3.8)
Hospitalizations	12 (60.0)	26 (81.3)	-	38 (73.1)
Deaths	2 (10.0)	2 (6.3)	-	4 (7.7)
Graft loss	1 (5.0)	2 (6.3)		3 (5.8)

^a^ During follow-up phase only serious infections with causality to study drug, hospitalizations and/or fatal outcome were reported.

AEs, adverse events; SAEs, serious adverse events

Prior to randomization, biopsy-proven acute rejection occurred in 31/199 (15.6%) of enrolled patients for whom data were available (3.9% [3/77] among randomized patients and 23.0% [28/122] in non-randomized patients; log rank p<0.001). Based on the safety population, the overall rate of dialysis prior to randomization at week 7 was 33.0% (16.9% [13/77] in the randomized group and 46.0% [58/126] in the non-randomized group). In addition, surgical complications before randomization were more common in the non-randomized patients compared to the randomized group (27.8% versus 13.0%) and more patients in the non-randomized group were reported to have diarrhea and vomiting compared to the randomized group (23.8% versus 7.8%, and 23.8 versus 10.4%, respectively). Proteinuria was also more frequent in the non-randomized group (20.6% versus 11.7%) (Table 3).

### Immunosuppression

Trough levels of everolimus and CsA, and MPA dosing, are summarized in Table 5. At randomization, 8 patients of the control group (8/24, 33.9%) and 16 patients of the everolimus group (16/51, 31.4%) were steroid-free according to the study protocol.

**Table 5:** Immunosuppression.

	Randomized (N = 77)	Non-Randomized (N = 124)
**Pre-randomization**		
*CsA trough level*, *ng/mL*		
Week 2	251.5 ± 205.7	200.2 ± 77.0
Week 7	153.6 ± 40.6	162.9 ± 77.4
*MPA*		
Mean dose, mg/day (including zero doses)	2181 ± 268	2295 ± 417
*Steroids*		
Immunosuppression containing corticosteroids (n, % of patients)	77 (100)	124 (100)
Dose pre-randomization, mg/daya		
Mean (SD)	80.3 (70.4)	95.4 (101.7)
Median (range)	67.0 (18.2, 333.3)	62.8 (10.0, 500.0)
**Post-randomization**	Control N = 24	Everolimus N = 51
*CsA or everolimus trough level*, *ng/mL*	*CsA*	*Everolimus*
Week 8	137.1 ± 39.3	7.4 ± 4.4
Week 12	147.7 ± 84.5	6.8 ± 2.8
Month 6	122.4 ± 36.6	6.1 ± 1.5
Month 12	111.0 ± 33.0	7.2 ± 3.9
*MPA*		
Mean dose, mg/day (including zero doses)	1389 ± 388	1399 ± 317
*Steroids*		
Immunosuppression containing corticosteroids at randomization (n, % of patients)	16 (66.7)	35 (68.6)
Immunosuppression containing corticosteroids Month 12 (n, % of patients)	11 (61.1)	20 (64.5)
Dose post-randomization, mg/day^a^		
Mean (SD)	28.4 (44.3)	87.4 (127.4)
Median (range)	7.6 (4.5, 175,0)	15.0 (4.0, 500.0)

^a^ Excluding patients in whom steroids had been permanently discontinued

Values are shown as mean ± SD

CsA, cyclosporine; MPA, mycophenolic acid (enteric-coated mycophenolic acid)

### Graft function

**Fig 3:**
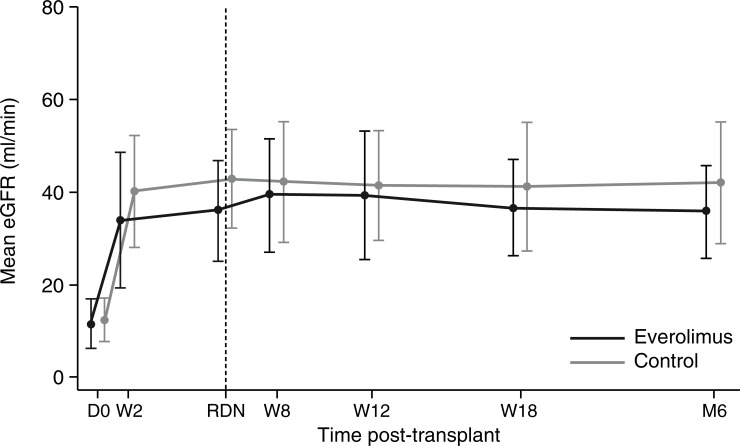
Observed eGFR (Cockcroft-Gault) to month 6 post-transplant according to treatment group. Values are shown as mean (SD). eGFR, estimated glomerular filtration rate; RDN, randomization (week 7).

Fig 3 shows the observed mean eGFR according to treatment group. At month 6, the mean eGFR (Cockroft-Gault method) was 42.0±13.0 ml/min in the control group and 36.5±10.8 ml/min in the everolimus group. Using the LS means adjusted for randomization from the ANCOVA analysis, the point estimate for the difference between the two groups (everolimus minus control) was -0.72 ml/min with a 95% CI of -5.93 to 4.5 ml/min. Thus, no relevant difference between the two treatment regimens was detectable in this exploratory analysis (p = 0.784). The same analysis was performed for eGFR determined according to Nankivell and MDRD methods, and the results were very similar to those obtained using the Cockroft-Gault method. Therefore, no relevant differences between the two groups were detectable.

### Efficacy events between randomization and month 6

**Fig 4:**
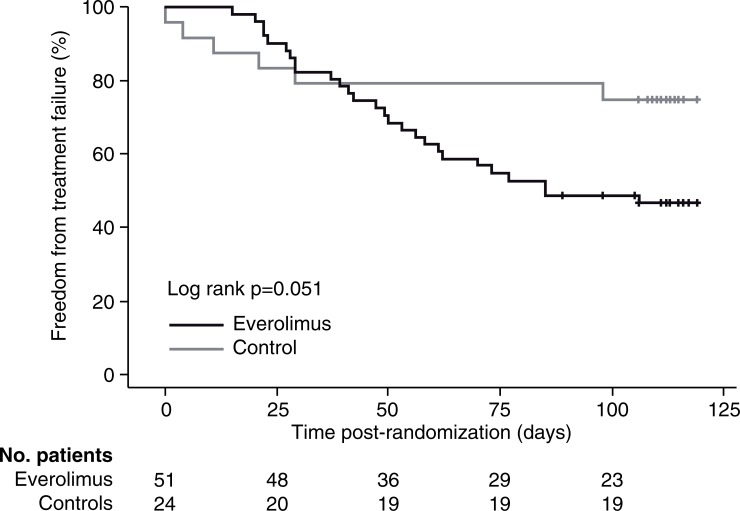
Freedom from treatment failure from randomization (day 0) to month 6 according to treatment group (Kaplan-Meier estimates). Vertical marks indicates patients censored at their last study visit.

No patient in either treatment group lost their graft, died or was lost to follow-up after randomization. For all other events, the Kaplan-Meier estimates were numerically higher in the everolimus group compared to the control group. Between-group comparisons of the estimated incidences of ‘discontinuation due to AE (27.8% versus 0.0%, log rank p = 0.005) and ‘conversion to another regimen' (32.5% versus 4.3%, log rank p = 0.007) were significantly in favor of the control group. As a consequence, the estimated incidence of treatment failure showed a trend to being higher in the everolimus than in the control group (53.2% versus 25.0%, log rank p = 0.051) (Fig 4) (Table 6).

**Table 6:** Incidence of efficacy events between randomization and month 6.

	Control (N = 24) n (%)^a^	Everolimus (N = 51) n (%)^a^	P-value (Log rank test)
Graft loss	0 (0.0)	0 (0.0)	-
Death	0 (0.0)	0 (0.0)	-
Lost to follow-up	0 (0.0)	0 (0.0)	-
Biopsy-proven acute rejection (BPAR)	5 (20.8)	12 (29.2)	0.711
Discontinuation due to lack of efficacy	1 (4.2)	9 (21.1)	0.075
Discontinuation due to adverse event	0 (0.0)	13 (27.8)	**0.005**
Conversion to another regimen	1 (4.3)	16 (32.5)	**0.007**
Treatment failure (composite endpoint)^b^	6 (25.0)	27 (53.2)	0.051
eGFR at month 6 (Nankivell)^c^	52.1	53.1	0.798
eGFR at month 6 (MDRD)^c^	38.6	40.0	0.652
Serum creatinine level (μmol/l)^c^	162.8	158.3	0.716

^a^ n is the number of patients with the respective event. Percentages are calculated as Kaplan-Meier estimates

^b^ Multiple events per patient were possible

^c^ LS mean values

p values <0.05 are shown in bold.

eGFR, estimated glomerular filtration rate; LS, least means; MDRD, Modification of Diet in Renal Disease

After randomization, the incidence of biopsy-proven acute rejection was 29.2% in the everolimus group versus 20.8% in the control group (p = 0.71).

### Safety results between randomization and month 6

The overall incidence of AEs was similar in the everolimus group and in the control group between randomization (week 7) and month 6 (98.0% and 87.5%, respectively). The AE categories in which AEs were more than 10% higher in the everolimus group versus the control group were infections (80.4% versus 62.5%), blood and lymphatic system disorders (58.8% versus 20.8%), metabolism and nutrition disorders (54.9% versus 25.0%), gastrointestinal disorders (54.9% versus 12.5%), and respiratory, thoracic and mediastinal disorders (23.5% versus 12.5%).

In both groups, the most common AE was urinary tract infection, but the incidence was higher in the everolimus group than in controls (49.0% versus 37.5%). Other AEs for which the incidence was more than 10% larger in the everolimus group versus controls were leukopenia (45.1% versus 16.7%), diarrhea (21.6% versus 0.0%), hypokalemia (23.5% versus 0.0%), and proteinuria defined as protein/creatinine ≥ 500 mg/g (15.7% versus 0.0%). Interestingly peripheral edema occurred more often in the control group (29.2% versus 25.5%) as well as pyrexia (16.7% versus 11.8%), dyspnea (12.5% vs 5.9%) and blood crea increase (20.8% vs 17.6%). (Table 7).

**Table 7:** Most frequent adverse events (AEs).

	Control (N = 24) n (%)	Everolimus (N = 51) n (%)
Number (%) of patients with AEs	21 (87.5)	50 (98.0)
**AEs**		
Anemia	2 (8.3)	7 (13.7)
Blood creatinine increased	5 (20.8)	9 (17.6)
Diarrhea	0 (0.0)	11 (21.6)
Dyspnea	3 (12.5)	3 (5.9)
Hypertension	3 (12.5)	6 (11.8)
Hypokalemia	0 (0.0)	12 (23.5)
Leukopenia	4 (16.7)	23 (45.1)
Peripheral edema	7 (29.2)	13 (25.5)
Proteinuria	0 (0.0)	8 (15.7)
Pyrexia	4 (16.7)	6 (11.8)
Urinary tract infection	9 (37.5)	25 (49.0)

Thirty-eight of the 51 randomized patients in the everolimus group (74.5%) experienced AEs assessed as being causally related to everolimus treatment. The everolimus-related AEs that occurred in at least 3 patients were leukopenia (25.5%), aphthous stomatitis (17.6%), urinary tract infection (13.7%), proteinuria (11.8%), pancytopenia (5.9%), and pneumonia (5.9%).

Between randomization and month 6, the incidence of SAEs was 54.9% in the everolimus group and 45.8% in the control group. AEs leading to permanent study drug discontinuation or requiring study drug dose adjustment/interruption occurred in 30.2% and 17.0% of everolimus-treated patients, respectively, and in no control patients. The most frequent reasons for study drug discontinuation in the everolimus group were gastrointestinal disorders (9.8%), blood and lymphatic system disorders (7.8%), and infections (7.8%).

Two patients in the everolimus group (3.9%) developed tumors (one parathyroid adenoma and one basal cell carcinoma). There were no tumors in the control group. New-onset type II diabetes mellitus developed in 1 patient (2.0%) in the everolimus group and none of the control patients.

### Efficacy and safety results during the follow-up phase

Fifty-two patients were included in the follow-up phase (32 in the everolimus group, 20 in the control group. Two patients died in each group, due to glioblastoma and fungal sepsis in the everolimus group, and due to bronchial carcinoma and unknown causes in the control group. There were two graft losses in the everolimus group (both due to chronic rejection) and one graft was lost in the control group (due to unknown reasons). The incidence of acute rejection during follow-up was 21.9% (7/32 patients) and 10% (2/20 patients) in the everolimus and control groups, respectively.

During follow-up, the following AEs were documented: severe infections, new-onset diabetes mellitus and tumors. Severe infections occurred in 75.0% of patients (24/32) in the everolimus group and in 40.0% of patients (8/20) in the control group. Two patients in each treatment group were diagnosed with tumors during the follow-up phase (melanoma and multifocal carcinoma in the everolimus group, and ovarian carcinoma and squamous cell carcinoma in the control group). One patient in each group had a new onset of diabetes mellitus. EGFR in the follow-up phase after 6 months is given in Table 8.

**Table 8:** eGFR (Cockcroft-Gault) of patients included in follow-up phase.

Mean eGFR ± SD (mg/dl), follow-up phase	Control (N)	Everolimus (N)
Month 6	43.0 ± 12.5 (19)	35.6 ± 11.2 (21)
Month 12	43.0 ± 10.9 (18)	35.3 ± 12.7 (31)
Month 24	41.9 ± 14.9 (16)	33.4 ± 12.1 (23)
Month 36	46.3 ± 10.8 (13)	33.9 ± 11.1 (18)

## Discussion

This prospective, randomized trial is the first to compare the efficacy and safety of early conversion to a CNI-free regimen comprising everolimus with MPA and anti-IL-2R induction therapy versus a standard CNI-based immunosuppressive regimen in KTx recipients over the age of 65 receiving ECD kidneys.

Although almost a third of all patients receiving a KTx in the Eurotransplant region are now older than 65 years, data are scarce regarding the optimal immunosuppressive regimen in this subgroup. For many years, patients over the age of 65 have been systematically excluded from randomized trials, exacerbating the lack of relevant data [30]. This is particularly problematic because KTx patients over the age of 65 years are known to have more peri-operative complications, a more complex post-transplant course with regards to co-morbidity, and increased risk of side effects from immunosuppressive drugs [10].

These difficulties are clearly demonstrated in the present study, in which only 37% of the 207 enrolled patients could be randomized at week 7. The main reason for non-randomization was AEs, especially surgical complications, but proteinuria and gastrointestinal disorders also occurred in a relatively high number of patients. This high rate of AEs, especially surgical complications and delayed graft function (DGF), are consistent with previous experience in elderly patients. KTx from ECD grafts are known to have significantly increased rates of DGF [31], which is associated with a higher risk of graft dysfunction and acute rejection [32]. DGF substantially increases the time to recovery of graft function, and GFR may still be improving later than in younger patients or with higher-quality grafts. This presents difficulties if study protocols randomize patients to an intervention too early. A more flexible randomization period, or later randomization (e.g. at 12–16 weeks after transplantation) might result in a higher proportion of ESP patients being eligible for randomization.

The primary endpoint of eGFR at 6 months post-transplant was similar in both groups, as was the rate of biopsy-proven acute rejection. Only a small number of patients entered the follow-up phase beyond 6 months after randomization. In these patients, eGFR remained stable and within the range of eGFR at randomization. However, discontinuation after randomization was frequent in the everolimus group (32% compared to 4% in the control group). This corresponds to findings from other conversion studies using mTOR inhibitors, which have reported discontinuation rates of up to 36% after 12 months [7, 22, 33, 34]. The incidence of SAEs was slightly higher in the everolimus group (55% versus 46% with controls), but this only partly accounted for the higher rate of study drug discontinuation due to AEs in the everolimus group compared to controls (17% versus 0%). Although confirmatory statistical analysis was not performed due to the low number of randomized patients these results demonstrate that discontinuation of everolimus due to AEs is common in ESP patients.

It should be noted that the high rate of gastrointestinal side effects seen prior to randomization was probably partly related to the increased MMF dose used to support early steroid withdrawal. In general, the combination of early conversion to everolimus at week 7 with early steroid withdrawal after week 2, and with a relatively high dose of MPA (2880 mg/day during week 1 and 2160 mg/day from week 2 to week 6 and thereafter), was too intensive in these vulnerable patients.

The question arises whether an early switch to CNI-free everolimus-based immunosuppression should be recommended at all in this fragile patient group, or whether the choice of regimen should be agreed prior to KTx and implemented *de novo* whenever feasible. Alternatively, the switch could be made at a later stage, when graft function and the patient's health has stabilized.

Compared to the ZEUS study, which showed a sustained benefit in renal function for patients converted to a CNI-free everolimus-based regimen [17, 34], the current study did not show any difference for graft function between the two groups. It remains to be investigated whether *de novo* everolimus in combination with low-dose CNI would preserve renal function more effectively in this patient group.

Rejection rates in elderly KTx patients are an ongoing subject of research. According to an analysis of United Network for Organ Sharing (UNOS) data, the frequency of rejection declines with increasing recipient age [35]. However, higher donor age is associated with increased rejection rates [36–38]. A recent study on the effect of HLA-DR mismatches in ESP patients confirmed donor age and DGF, as well as HLA-DR mismatches, to be independent risk factors for T-cell mediated rejection whereas older recipient age turned out to be protective [16]. In that study, the effect of recipient age was outweighed by the increased immunogenicity of the donor organ, and the rejection was associated with significantly decreased graft survival in the elderly KTx patients [16]. In the current trial, despite a highly mismatched population, the observed rejection rates did not differ significantly between groups up to month 6 post-transplant. Moreover, the rates were in line with recent data from elderly KTx patients in other studies [16], indicating no adverse effect of the steroid-free regimen.

As shown in this study, immunosuppressive regimens tailored to the patient's age, taking into account co-morbidities, complication rates and immunological specificities, are still needed. It is a major challenge to design randomized studies tailored to ESP patients, and to include elderly patients in more general randomized trials, but this is a priority given the need to collect reliable data in this growing subgroup of patients. For future studies involving elderly transplant patients, the high rate of non-randomization is very relevant to study planning and patient calculations. In addition the complexity of the current study protocol proved challenging for the participating centers, as evidenced by the fact that only a few steroid-free patients underwent randomization. Protocols should be designed accordingly and, particularly in the old-for-old setting, non-randomized patients should remain in follow-up so that the outcomes of all patients can be evaluated. We hope that our experience will help define future study protocols for this growing population.

## Supporting information

S1 File SENATOR clinical study protocol 08-Feb-2009. (PDF)

S2 File SENATOR clinical study protocol including Amendment No.1 13-May-2009. (PDF)

S3 File SENATOR clinical study protocol including Amendment No.2 26-May-2010. (PDF)

S4 File SENATOR clinical study protocol including Amendment No.3 06-Feb-2012. (PDF)

S5 File SENATOR clinical study protocol including Amendment No.4 06-Nov-2012. (PDF)

S6 File CONSORT checklist. (DOCX)
