# Supplementary material for: Everolimus in de novo kidney transplant recipients participating in the Eurotransplant senior program: Results of a prospective randomized multicenter study (SENATOR)
Source: PLoS One. 2019 Sep 19;14(9):e0222730. doi: 10.1371/journal.pone.0222730 (PMC6752944; doi:10.1371/journal.pone.0222730)
Supplement: S2 File — (PDF) [file pone.0222730.s002.pdf]

NOVARTIS

Certican® (Everolimus)

CRAD001ADE19

# SENATOR

# 6-month, open-label, randomized, multicenter, prospective, controlled study to evaluate the efficacy, safety and tolerability of Everolimus in *de novo* renal transplant recipients participating in the Eurotransplant senior program

| | |
|---|---|
| Author(s) | Prof. Dr. Klemens Budde, Dr. Christoph May, Dr. Stefan Kramer |
| Document type | Clinical Study Protocol including Amendment No.1 |
| Development phase | IIIb/IV |
| EudraCT-NO. | 2008-005109-20 |
| Document status | Final 2.0 |
| Release date | 13-May-2009 |
| Number of Pages | 100 |

Property of Novartis Pharma GmbH Nürnberg
Confidential

May not be used, divulged, published, or otherwise disclosed
without the consent of Novartis

## Table of contents

List of Post-text supplements ..... 5
List of tables ..... 5
List of abbreviations ..... 6
Glossary of terms ..... 8
Signature Pages ..... 9
Protocol Synopsis ..... 11
1 Background ..... 17
2 Study purpose ..... 28
3 Objectives ..... 28
4 Study design ..... 28
5 Population ..... 31
6 Treatment ..... 34
6.1 Components of Investigational therapies ..... 34
6.2 Treatment groups ..... 35
6.3 Treatment assignment ..... 35
6.4 Treatment blinding ..... 36
6.5 Treating the patient ..... 36
6.5.1 Study drug administration ..... 36
6.5.2 Permitted study drug adjustments ..... 38
6.5.3 Rescue medication ..... 40
6.5.4 Other concomitant treatment ..... 41
6.5.5 Study drug discontinuation ..... 42
6.5.6 Premature patient withdrawal from study treatment ..... 42
6.5.7 Emergency unblinding of treatment assignment ..... 43
7 Visit schedule and assessments ..... 44
7.2 Information to be collected on screening failures ..... 49
7.3 Patient demographics/other baseline characteristics ..... 50
7.4 Treatments ..... 50
7.5 Efficacy ..... 50
7.6 Safety ..... 52
7.7 Tolerability ..... 56
7.8 Health-related Quality of Life ..... 56
7.9 Pharmacokinetics ..... 57
7.10 Pharmacogenetics/pharmacogenomics ..... 57
7.11 Other biomarkers ..... 57

7.12 Additional Assessments ..... 58
8 Safety monitoring ..... 58
8.1 Serious adverse event reporting ..... 58
8.2 Pregnancies ..... 59
8.3 Data Safety Monitoring Board ..... 59
9 Data review and database management ..... 59
9.1 Site monitoring ..... 59
9.2 Data collection ..... 60
9.3 Database management and quality control ..... 60
10 Statistical methods ..... 60
10.1 Analysis set ..... 61
10.2 Background and demographic characteristics ..... 62
10.3 Study treatments ..... 62
10.3.1 Study medication ..... 62
10.3.2 Concomitant therapy ..... 62
10.4 Analysis of the primary objective(s) ..... 63
10.4.1 Variable ..... 63
10.4.2 Statistical hypothesis, model, and method of analysis ..... 63
10.4.3 Handling of missing values/censoring/discontinuations with respect to the primary efficacy variable ..... 63
10.4.4 Supportive analyses ..... 64
10.5 Analysis of secondary objectives ..... 64
10.5.1 Efficacy (secondary) ..... 64
10.5.2 Safety ..... 65
10.5.3 Tolerability ..... 67
10.5.4 Resource utilization ..... 67
10.5.5 Health-related Quality of Life ..... 67
10.5.6 Pharmacokinetics ..... 67
10.5.7 Pharmacogenetics/pharmacogenomics ..... 68
10.5.8 Biomarkers ..... 68
10.6. Interim analysis ..... 68
10.7 Sample size calculation ..... 68
11 Discussion and rationale for study design features ..... 68
12 References ..... 70
Appendix 1: Administrative procedures ..... 82
Appendix 2: Notable laboratory value criteria, special methods and scales ..... 84

Appendix 3: Possible drug interactions ........ 86
Appendix 4: Dose Reduction of Certican® and Myfortic® ........ 88
Appendix 5: National Cholesterol Education Program Guidelines ........ 93
Appendix 6: Definition of renal allograft rejection ........ 94
Appendix 7: Coronary Disease Risk Prediciton Score (Framingham Score) ........ 99

## List of Post-text supplements

## List of tables

Table 4-1 Study outline ........ 30
Table 7-1 Assessment schedule ........ 44

## List of abbreviations

| | |
|---|---|
| AE | Adverse Event |
| ALT (SGPT) | Alanine Aminotransferase (Serum Glutamate Pyruvate Transaminase) |
| AST (SGOT) | Aspartate Aminotransferase (Serum Glutamatoxalacetate Transaminase) |
| ATC | Anatomical Therapeutic Chemical dictionary |
| ATG | Antithymocyte Globulin |
| AUC | Area Under the Curve |
| Bid | *bis in diem*/twice a day (12 hours apart) |
| BL | Baseline |
| BPAR | Biopsy-Proven Acute Rejection |
| CI | Confidence Interval |
| Cmax | Maximum Plasma Concentration |
| CMV | Cytomegalovirus |
| CNI | Calcineurin Inhibitor |
| CPK | Creatinine Phosphokinase |
| CR | Clinical Research |
| CRF | Case Report/Record Form |
| CRO | Clinical Research Organization |
| CRP | C-Reactive Protein |
| CsA | Cylcosporine A |
| CV | Cardiovaskular |
| DSMB | Data Safety Monitoring Board |
| ECG | Electrocardiogram |
| ENR | Enrolled Patient Population |
| EC-MPS | Enteric Coated Mycophenolate Sodium (Myfortic®) |
| ESP | Eurotransplant senior program |
| FAS | Full Analysis Set |
| GFR | Glomerular Filtration Rate |
| GI | Gastrointestinal |
| GCP | Good Clinical Practice |
| ICH | International Conference on Harmonization |
| IEC | Independent Ethics Committee |
| IMPDH | Inosine 5’-Monophosphate Dehydrogenase |
| IRB | Institutional Review Board |
| HBsAg | Hepatitis B Surface Antigen |

| | |
|---|---|
| HDL | High-Density Lipoproteine Cholesterine |
| LDL | Low-Density Lipoproteine Cholesterine |
| MDRD | Modification of Diet in Renal Disease Study Group |
| MedDRA | Medical Dictionary for Drug Regulatory Affairs |
| mmHg | Millimeters of Mercury |
| MMF | Mycophenolate Mofetil |
| mTOR | Mammalian Target of Rapamycin |
| MPA | Mycophenolic Acid |
| NODM | New-onset Diabetes Mellitus |
| NSAID | Non-Steroidal Anti-Inflammatory Drug |
| OGTT | Oral Glucose Tolerance Test |
| PK | Pharmacokinetics |
| PP | Per-Protocol Set |
| PRA | Panel Reactive Antibody |
| RAD | Everolimus, Certican® |
| RAN | Randomized Patients Population (at Visit 3) |
| SAE | Serious Adverse Event |
| SAF | Safety Set |
| SL | Serum Level |
| SOP | Standard Operating Procedures |
| SRL | Sirolimus |
| TL | Trough Level |
| tmax | Time until Maximum Plasma Concentration |
| Tx | Transplantation |
| ULN | Upper Limit of Normal |
| WHO | World Health Organization |

## Glossary of terms

| | |
|---|---|
| Assessment | A procedure used to generate data required by the study |
| Control; control drug | A study drug used as a comparator to reduce assessment bias, preserve blinding of investigational drug, assess internal study validity, and/or evaluate comparative effects of the investigational drug |
| Enrollment | Point/time of patient entry into the study; the point at which informed consent must be obtained (i.e., prior to starting any of the procedures described in the protocol) |
| Investigational drug | The study drug whose properties are being tested in the study; this definition is consistent with US CFR 21 Section 312.3 and is synonymous with “investigational new drug.” |
| Patient number | A unique identifier assigned to each patient who enrolls in the study |
| Premature patient withdrawal | Point/time when the patient exits from the study prior to the planned completion of all study drug administration and assessments; at this time all study drug administration is discontinued and no further assessments are planned |
| Randomization number | A unique identifier assigned to each randomized patient, corresponding to a specific treatment group assignment |
| Study drug | Any drug administered to the patient as part of the required study procedures; includes investigational drug and any control drugs |
| Study drug discontinuation | Point/time when patient permanently stops taking study drug for any reason; may or may not also be the point/time of premature patient withdrawal |
| Variable | Information used in the data analysis; derived directly or indirectly from data collected using specified assessments at specified timepoints |

# Signature Pages

## Certican® (Everolimus)

**Protocol number:** **CRAD001ADE19**

**Title:** **SENATOR**

**6-month, open-label, randomized, multicenter, prospective, controlled study to evaluate the efficacy, safety and tolerability of Everolimus in *de novo* renal transplant recipients participating in the Eurotransplant senior program**

**EudraCT-No.:** **2008-005109-20**

## Approved by the following

### Signatures of Novartis Personnel:

Dr. Stefan Kramer
(Head Therapeutic Area) signature date

Dr. Eva-Maria Vogel
(Clinical Trial Leader) signature date

Dr. Christoph May
(Trial Statistician) signature date

### Signature of Co-ordinating Investigator:

Prof. Dr. Klemens Budde
(Co-ordinating Investigator) signature date

## Signature Page for Investigator

### Certican® (Everolimus)

**Protocol number:** **CRAD001ADE19**

**Title:** **SENATOR**

**6-month, open-label, randomized, multicenter, prospective, controlled study to evaluate the efficacy, safety and tolerability of Everolimus in *de novo* renal transplant recipients participating in the Eurotransplant senior program**

**EudraCT-No.:** **2008-005109-20**

I have read this protocol and agree to conduct this trial in accordance with all stipulations of the protocol and in accordance with the principles outlined in the Declaration of Helsinki.

(Investigator) ______________________________ Signature ____________ Date

# Protocol Synopsis

**Title of study:**

6-month, open-label, randomized, multicenter, prospective, controlled study to evaluate the efficacy, safety and tolerability of Everolimus in de novo renal transplant recipients participating in the Eurotransplant senior program.

**Study purpose:**

This study wants to address whether a CNI-free regimen six weeks after transplantation for ESP patients is as safe and well tolerated as standard treatment but optimising immunosuppressive therapy with benefits in renal function, NODM, CV risk, cancer and allograft nephropathy.

**Objectives:**

**Primary**

- Renal function assessed by glomerular filtration rate (GFR) – Cockcroft-Gault method – 6 months after renal transplantation (Tx)

**Secondary**

- To assess renal function by GFR – MDRD and Nankivell method – at Month 6 post Tx
- To assess renal function by serum creatinine at Month 6 post Tx
- To assess efficacy (biopsy proven acute rejection, graft loss, death) at Month 6
- To assess occurrence of treatment failures up to or at Month 6, while treatment failure is defined as a composite endpoint of biopsy proven acute rejection, graft loss, death, loss to follow up and discontinuations due to lack of efficacy or toxicity or conversion to another regimen (at least one condition must be present)
- To assess evolution of renal function (creatinine slope) between Week 7 (randomization) and Month 6
- To assess CD25 saturation on lymphocytes
- To assess safety and tolerability at Month 6:
  - AE / SAE
  - incidence of infections (CMV, BK virus)
  - tumor incidence
  - cardiovascular risk (changes in Framingham score, ECG)
  - occurrence of proteinuria
  - NODM

**Population:**

The study population consists of a representative group of approx. 240-260 senior de novo kidney transplant patients (patients will be randomized in a 1:2 ratio at Week 7 to either the Control or the Certican group; 82 patients in Control arm, 162 patients in Certican arm). Enrollment will be continued until the required sample size will be achieved. The patients will be recruited from about 8 – 12 transplant centers in Germany.

**Inclusion/Exclusion criteria:**

To be fulfilled at Baseline visit (Screening visit prior to transplantation)

**Inclusion Criteria**

1. Males or females, aged > 65 years, receiving a primary kidney from a donor aged > 65 years in the Eurotransplant Senior Program
2. Recipients of de novo cadaveric kidney transplants
3. Patients who are willing and able to participate in the study and from whom written informed consent has been obtained

**Exclusion Criteria**

1. Multi-organ recipients (e.g., kidney and pancreas)
2. Patients receiving a kidney from a non-heart beating donor
3. Patients who are recipients of A-B-O incompatible transplants
4. Patients with a historical (or current) peak PRA of > 25% (current = 3 months)
5. Patients with already existing antibodies against the HLA-type of the receiving transplant
6. Patients with any known hypersensitivity to Simulect®, Certican®, mycophenolic acid, cyclosporine A, other drugs similar to Certican® (e.g., macrolides), or other components of the formulations (e.g. lactose)
7. Patients who have received an investigational immunosuppressive drug within four weeks prior to study entry (Baseline visit 1)
8. Patients with thrombocytopenia (platelets < 75,000/mm³), with an absolute neutrophil count of < 1,500/mm³ or leucopenia (leucocytes < 2,500/mm³), or hemoglobin < 6 g/dL
9. Patients with symptoms of significant somatic or mental illness. Inability to cooperate or communicate with the investigator, who are unlikely to comply with the study requirements, or who are unable to give informed consent
10. Patients who are HIV, HCV RNA, or Hepatitis B surface antigen positive
11. Evidence of severe liver disease (incl. abnormal liver enzyme profile, i.e. AST, ALT or total bilirubin > 3 times UNL)
12. Females at randomization who will be not considered post-menopausal according to the following definition:

    = 12 months of natural (spontaneous) amenorrhea or

    = 12 month post surgical bilateral oophorectomy or

    hysterectomized women over or equal to 65 years of age
13. Presence of a clinically significant infection requiring continued therapy, severe diarrhea, active peptic ulcer disease, or uncontrolled diabetes mellitus that in the opinion of the investigator would interfere with the appropriate conduct of the study
14. History of malignancy of any organ system, treated or untreated, within the past 5 years whether or not there is evidence of local recurrence or metastases, with the exception of localized basal cell carcinoma of the skin
15. Evidence of drug or alcohol abuse
16. Patients receiving drugs known to strongly interact with CsA and/or everolimus according to the list provided in Appendix 3 to this protocol should be excluded, if in the opinion of the investigator this drug interaction interferes with the objectives of the study, namely a clinical meaningful potentiation of renal dysfunction and/or maintenance of adequate immunosuppressive drug levels

To be fulfilled at Baseline 2 (prior to randomization)

In addition to the above criteria the following must be met at BL2 prior to randomization.

**Inclusion Criteria**

1. Patients with an stable serum creatinine < 3.0 mg/dl
2. Patients have to be on an immunosuppressive regimen with ≥ 720 mg/d myfortic® and Sandimmun® Optoral
3. Patients with a proteinuria < 500 mg/g creatinine

**Exclusion Criteria**

1. Graft loss after current transplantation (since Baseline visit 1)
2. Patients who received a depleting antibody therapy or more than 10mg/day prednisolon (or an equivalent oral steroid therapy)
3. Patients who suffered from rejection (more than BANFF 1A), recurrent acute rejection, or steroid resistant acute rejection
4. Patients with thrombocytopenia (platelets < 75,000/mm³), with an absolute neutrophil count of < 1,500/mm³ or leucopenia (leucocytes < 2,500/mm³), or hemoglobin < 6 g/dL
5. Evidence of severe liver disease (incl. abnormal liver enzyme profile, i.e. AST, ALT or total bilirubin > 3 times ULN)
6. Current dialysis dependency at BL2 visit
7. Patients with clinically significant infection requiring continued therapy which would interfere with the objectives of the study
8. Presence of intractable immunosuppressant complications or side effects (e.g., severe gastrointestinal adverse events) at BL2 visit (Week 7)

**Components of Investigational and reference therapies**

**Investigational drug**

- **Certican®**

| | |
|---|---|
| Active ingredient: | Everolimus (RAD001) |
| Galenic form: | Tablets |
| Dose: | one tablet containing 0.5 mg, 0.75 mg or 1.0 mg |
| Dosing schedule: | initially 3 mg on day 1 and 2 x 1.5 mg on day 2 after randomization; afterwards based on blood level (5-10 ng/mL in CNI-free regimen) |
| Packaging: | Blisters of 10 Tablets |

- **Simulect®**

| | |
|---|---|
| Active ingredient: | Basiliximab |
| Galenic form: | lyophilisate in vials with ampoules of sterile water for injection (5 mL) |
| Dose: | one vial containing 20 mg lyophilisate |
| Dosing schedule: | 2 x 20 mg (day 0 (2 hrs prior to Tx) and day 4 post Tx) in Control and Certican group. Additional doses of Simulect (each 20 mg) in week 7 & 12 for patients randomized to Certican group to be applied as i.v. bolus injection |
| Packaging: | packages of 1 lyophilisate in vial with ampoules of sterile water |

**Other immunosuppressive drugs**

- **Sandimmun® Optoral**

| | |
|---|---|
| Active ingredient: | Cyclosporine A |
| Galenic form: | capsules |
| Dose: | one capsule containing 10, 25, 50, or 100 mg |
| Dosing schedule: | according to blood level |
| Packaging: | trade ware will be used |

- **Myfortic®**

| | |
|---|---|
| Active ingredient: | Enteric Coated Mycophenolate Sodium (EC-MPS) |
| Galenic form: | Tablets |
| Dose: | one tablet containing 180 mg or 360 mg |
| Dosing schedule: | "Loading Dose"-Myfortic concept for both arms<br>Week 1-2 2880 mg/d, Week 3-6 2160 mg/d, Week 7-24 1440 mg/d; dose reduction due to side effects are possible (minimal dose at BL2: 720 mg/d) |
| Packaging: | trade ware will be used |

- **Corticosteroids**
  according to local standard. A minimum dose of 5 mg prednisolon or equivalent should be administered. Steroids should be withdrawn after week 2 in all patients.

---

**Study design**

Prospective, multi-center, randomized, controlled, parallel group, open-label study in *de novo* senior renal transplant recipients.

For the first 6 weeks post transplantation, all patients will be treated with Simulect® (induction therapy) and immunosuppressive regimen consisting of Myfortic® + Sandimmun® Optoral + corticosteroids (steroids only first 2 weeks).

Control Assessments for renal function will be performed 2 weeks after transplantation.

At Baseline [BL]2 (Visit 3, Week 7) all eligible patients (according to in- and exclusion criteria as specified for BL2) will be randomized to one of the two treatment groups in a 1:2 ratio, stratified by occurrence of rejections between BL1 and BL2:

| | |
|---|---|
| **Group 1: Control** | **maintain prior immunosuppressive regimen consisting of**<br>Myfortic® + Sandimmun® Optoral |
| **Group 2: Certican®** | **switch immunosuppressive therapy in the following steps** |
| Step 1: | Day 1 morning: Myfortic® + Sandimmun® Optoral<br>Day 1 evening: Myfortic® + Sandimmun® Optoral + Certican® (3 mg) |
| Step 2: | Day 2 morning: Myfortic® + Certican® (1.5 mg)<br>Day 2 evening: Myfortic® + Certican® (1.5 mg) |
| Step 3: | Day 3 morning: Myfortic® + Certican® (Trough Level [TL] 5-10 ng/mL) |

In group 2, step 1 of the therapy switch will be performed the day after randomization and will be continued on day 2 with step 2. The therapy switch will be completed on day 3 with step 3.

Patients randomized to Certican group will receive two additional doses of Simulect® (each 20 mg) as rejection prophylaxis in week 7 and 12.

The established treatment will be continued until Month 6 (final assessment). Control assessments will be performed at Week 12, 18 and 24 (end of study and early discontinuation) post Tx. Renal function (creatinine, GFR) as well as safety and efficacy measures will be assessed at every study visit.

Additionally, on day 7 ± 2 after randomization a blood level control will be performed.

**Efficacy assessments**

- Glomerular Filtration Rate (GFR), calculated according to Cockcroft-Gault method (primary endpoint), MDRD method, and Nankivell-method (secondary endpoints)
- Serum creatinine and slope of creatinine for assessments after BL2
- Rejection episodes (treated and biopsy proven), graft loss, death, lost to follow up, as well as treatment failure as a composite endpoint.

**Other assessments**

- Change in immunosuppressive therapy
- Infections (Cytomegalovirus [CMV], BK virus)
- Tumor incidence
- (Serious) Adverse Events
- Physical Examination
- Vital signs
- Cardiovascular risk (Framingham Score, ECG)
- (Safety) Laboratory evaluation
- Blood levels of cyclosporine (C0-h level) and Certican® (everolimus, C0-h level)
- New-onset of diabetes mellitus (NODM)
- CD25 saturation on lymphocytes

**Data analysis**

The data will be analyzed by Novartis and/or by the designated CRO when all patients have completed the study treatment at Visit 6 (Week 24/Month 6) or discontinued prematurely. Data will be summarized with respect to demographic and baseline characteristics, efficacy observations and measurements, safety observations and measurements, and pharmacokinetic measurements. All summary statistics will be presented by treatment group. Categorical variables will be summarized by absolute and relative frequencies. Continuous variables will be summarized by descriptive statistics of mean, standard deviation, minimum, median and maximum. Time-to-event data including rates of affected patients will be assessed by Kaplan-Meier statistics.

The primary variable for assessment of renal function is the glomerular filtration rate (GFR) at Month 6, as assessed by the Cockcroft-Gault method (recalculated values). The hypotheses will be tested with an analysis of covariance (ANCOVA) with treatment, center, and occurrence of rejections between BL1 and BL2 as factors, and GFR at Visit 3 (Baseline 2) as covariate for the full analysis set (FAS). Raw- as well as adjusted (LS-) means will be provided as point estimates for all pair-wise treatment contrast. Two-sided, 95% confidence intervals and p-values for the null hypothesis of no treatment difference will be calculated for all treatment contrasts. For superiority testing, LS-means of the standard and CNI-free regimen will be compared with a 2-sample-test. Missing data with respect to the primary efficacy parameter will be dealt with multiple imputation. Supportively, the primary analysis will be performed for the per-protocol set. Additionally, data will be analyzed with an ANCOVA after having imputed missing values by last observation carried forward (LOCF) for the FAS.

The assessment of safety will be based mainly on the frequency of adverse events and on the number of laboratory values that fall outside of pre-determined ranges. Adverse events will be summarized by presenting, for each treatment group, the number and percentage of patients having any adverse event, having an adverse event in each body system and having each individual adverse event. Any other information collected (e.g. severity or relatedness to study medication) will be listed as appropriate. Other safety data (e.g. electrocardiogram, vital signs, special tests) will be considered as appropriate.

**Sample size**

From data for the subgroup of elderly patients in study CRAD001A2418, the probable difference in the GFR (Cockcroft-Gault) between the CNI-free regimen group and the standard regimen group is estimated as $\delta$ = 8 ml/min with a standard deviation of 16 ml/min at Month 6 after transplantation. Assuming a difference in the mean GFR of 8 ml/min (60 ml/min in the CNI-free group and 52 ml/min in the standard regimen group) and a common standard deviation of 19 ml/min, a two-group t-test with a 5% two-sided significance level will have 80% power to detect this difference when the sample sizes in the CNI-free group is 135 and in the standard regimen group is 68, respectively (a total sample size of 203). Taking into account a common drop-out rate of 10%, 162 patients need to be randomized into the CNI-free group, and 82 into the standard regimen group (a total drop-out adjusted sample size of 244 patients). Enrollment will be continued until the required sample size will be achieved.

# 1 Background

The ultimate goal of immunosuppressive therapy in transplantation is to provide an efficacious regimen without compromising safety and minimizing non-immune toxicities. By combining synergistic drugs, it may be possible to reduce the exposure to the individual agents and therefore avoid or reduce drug related toxicities whilst maintaining favorable clinical outcomes. As the population ages, the incidence and prevalence of elderly patients with end-stage renal disease also increases. This has subsequently led to a dramatic increase in the number of older patients on waiting lists to receive a renal transplant (US Renal Data System; Annual Data Report 2003). Older age alone should not be a hindrance to eligibility for allograft transplantation with recent clinical studies providing evidence of the viability of this treatment option for older patients with end-stage renal disease who receive organs from donors of any age (Fabrizii et al., 2005; Meier-Kriesche et al., 2000) and for those who receive organs from older donors (Smits et al., 2002; Frei et al., 2008; Fritsche et al., 2003).

Progress has been made in transplantation medicine with the introduction of calcineurin inhibitors (CNIs) such as **cyclosporine** (CsA) and **tacrolimus**. Besides effective immunosuppression for new and stable transplant patients, a major therapeutic benefit of that generation of drugs particularly consists in the reduction in early severe acute rejections. In the longer run, however, CsA (Nankivell et al., 2003) does not reduce the development of chronic transplant nephropathy. Conversely, both CNIs may even cause both acute and chronic nephrotoxicity, aggravating the long-term decline in renal function. CNIs not only have a direct negative impact on renal blood flow and glomerular filtration rate (GFR) but may lead to tubulointerstitial fibrosis and arteriolopathy (Fellström 2004). CNIs have also been shown to worsen or promote new cardiovascular risk factors such as hypertension, hypercholesterolemia, and diabetes mellitus after transplantation (Moien-Afshari et al., 2003). While CsA favors the development of hyperlipidemia, tacrolimus was shown to be pro-diabetogenic, especially in an elderly population (Webster et al. 2005; Vincenti et al. 2007). There has, therefore, been an increasing interest to explore combinations of treatment that allow for reduction in CNI exposure, as this may potentially enhance long term outcomes.

In the mid-1990ies, the use of **mycophenolate mofetil** (MMF), the morpholino ethylester prodrug of mycophenolic acid (MPA), has been documented to be safe and effective in the prevention of organ rejection in renal transplantation when used in combination with cyclosporine and corticosteroids (Srinvas et al, 2003). MMF is an approved drug product in the U.S. and Europe. Reduced exposure of MPA was observed when MMF and CsA were administered concomitantly. This is probably caused by decreased secretion of MPA into the bile, which in turn leads to reduction of enterohepatic recirculation (Shaw et al, 2003; Srinivas et al., 2003). In order to avoid the nephrotoxicity associated with CNIs several clinical trials were performed, in which CsA was replaced by MMF in the first two years after transplantation (Smak

Gregoor et al., 2002; Vincenti et al., 2001; Abramowicz et al., 2001, Abramowicz et al., 2005, Ekberg et al., 2007a). In contrast to reports on successful CsA withdrawal in maintenance patients > 4 years after transplantation (Suwelack et al., 2004, Dudley et al., 2002; Houde et al., 2000), early CNI withdrawal was clearly associated with an increased risk for acute rejection, suggesting that the potency of MMF is not sufficient to allow early elimination of CNIs.

Another new therapeutic option in the prevention of (renal) transplant rejection is **enteric coated mycophenolate sodium (EC-MPS)** that had been developed with the objective to enhance upper gastrointestinal (GI) tolerability. Under the name of **Myfortic®** the drug has been approved in Europe since 2004. EC-MPS 720 mg was shown to deliver bioequivalent MPA exposure (AUC) to MMF 1000 mg (Arns 2005, Budde 2007). Similar to MMF, concomitant immunosuppressive therapy has been shown to influence MPA exposition differently: MPA exposure was 19% lower under CsA therapy compared to tacrolimus treatment (Kaplan et al., 2005). Salavadori and coworkers (2004) demonstrated in a 12-month double-blind study that EC-MPS is therapeutically equivalent to MMF in de novo renal transplant patients regarding biopsy-proven acute rejection (BPAR), graft loss and death, the overall safety profile and incidence of GI adverse events. In another double-blind 12-month study, an international study group (Budde et al., 2004) showed that stable renal patients can be converted from MMF to Myfortic® (EC-MPS) without compromising the safety and efficacy profile associated with MMF. Moreover, GI adverse event severity tended to be lower, and the number of serious infections was significantly lower in EC-MPS patients. A recent report from a German multicenter study involving 79 de novo patients after renal transplantation (Budde et al., 2007) assessed the safety and efficacy of Myfortic® in combination with Simulect®, steroids and either full or reduced dose Sandimmun® Optoral in *de novo* kidney transplant recipients at 12 months post transplantation. This study resulted in excellent tolerability of this combination therapy, with an overall patient and graft survival rate of 95.5% after 1 year. The incidence of BPAR was only 16.9% after 12 months. The authors concluded that Myfortic® in combination with Simulect®, Neoral® and steroids is an effective and safe combination in *de novo* kidney transplant recipients. In addition this regimen allows lower Sandimmun® Optoral target levels and calcineurin inhibitor dose reduction without compromizing immunosuppression and safety. And yet, such combined regimen is not entirely devoid of the nephrotoxic hazards inherent to treatments with CNIs. A very recent study (Budde et al., ICTS 2008) investigated, whether an initially intensified dosing regimen with EC-MPS results in a higher proportion of patients reaching therapeutic MPA exposure early after transplantation. In contrast to patients with standard EC-MPS dose, who achieved adequate MPA exposure after a median of 43 days, the intensified dosing regimen resulted in adequate MPA exposure after a median of 7 days. First data indicate no safety concern of the intensified dosing regimen, however there was a tendency towards better efficacy. Thus, an initially intensified MPA regimen may allow to further reduce concomitant immunosuppressive therapy, e.g. to enable an early steroid withdrawal. So far, early steroid withdrawal on the basis of Basiliximab, EC-MPS and CsA resulted in an increased rejection rate, however without compromising graft survival (Vincenti et al.,

2008). There is a general consensus, that steroid withdrawal is especially preferable in patients with a high cardiovascular risk and elderly patients, which are more likely to develop severe steroid associated side effects such as diabetes mellitus.

The introduction of the potent immunosuppressive agent sirolimus (SRL, Rapamune, rapamycin, RAPA) offered a novel opportunity to develop a regimen designed to maximize prophylaxis of early acute rejection, without inherent drug-induced nephrotoxicity. Because sirolimus and CNIs exhibit strong synergistic properties with respect to rejection prophylaxis, initial attempts aimed at combining sirolimus with CNIs (Kahan B. et al. 1996, Kahan B et al. 2000, Yakupoglu YK et al. 2003). However, soon it was discovered that sirolimus also potentiates CNI-associated side effects, most importantly CNI associated nephrotoxicity (Kuypers DR 2005). As a consequence subsequent trials investigated lower CNI doses for the combination therapy with sirolimus. (Baboolal K. 2003). At 3 months, 87 patients were randomized 1:1 to CsA elimination or CsA dose minimization. While rejection rates in both arms were low after randomization, withdrawal of CsA from a small-dose sirolimus maintenance regimen was safe and was associated with an improvement in renal function. However it is important to note that CsA levels in the minimization arm were not as low as anticipated. Another large multicentre study (Vitko et al. 2006) compared the efficacy of combining tacrolimus and two different dosages of sirolimus (0,5 and 2mg) with an established tacrolimus MMF (1g) regimen. The incidence of biopsy-proven acute rejection was lowest in the higher (2mg) sirolimus dose group (15.7%) compared with the lower sirolimus (0.5mg) dose group (25.2%, $p = 0.003$) and the tacrolimus 1g MMF control group (22.3%, $p = 0.036$). Although combining 2 mg sirolimus with tacrolimus resulted in lower rates of acute rejection, this combination was associated with a higher incidence of adverse events and worse renal function. Lo and coworkers (2004) designed a study to determine the optimal dosage combinations of tacrolimus and sirolimus in a high-risk renal transplant population with cadaveric grafts. Both treatments, standard tacrolimus (trough 10-15 ng/mL) plus reduced sirolimus (trough 5-10 ng/mL) versus reduced tacrolimus (trough 5-10 ng/mL) plus standard sirolimus (trough 10-15 ng/mL), were associated with a low risk of acute rejection. However, 50% of patients who received standard tacrolimus and reduced sirolimus had to be discontinued from treatment because of biopsy-proven nephrotoxicity. This is another piece of evidence for the adverse effects of regimens based on CNIs in combination with sirolimus. So far, an increased rate and severity of CNI toxicity (most importantly nephrotoxicity) was observed in all studies in which CNIs were used in combination with sirolimus (Kuypers et al. 2005; Oberbauer et al. 2005). In a recent retrospective analysis of a large American database (Meier-Kriesche 2004, 2005) the combination of CNI and sirolimus was associated with significantly worse graft survival and death-censored graft survival compared to CsA and MMF. Although this likely reflects the known detrimental effects of full-dose CsA treatment in combination with sirolimus, and alternative strategies of sirolimus utilization with reduced CsA may yield better outcomes, this analysis argues against the long-term use of the combination therapy of mTor inhibitors with standard doses of CNIs.

Multicenter trials have demonstrated the beneficial effects of CsA withdrawal in terms of improved renal function in de novo renal transplant patients initially receiving CsA, SRL and steroids (Johnson, 2001; Hricik, 2001). In an open-label study (Johnson et al., 2001) renal allograft recipients at Month 3 were randomized to remain on the previous sirolimus-CsA-steriod regimen or to have CsA withdrawn and therapy continued with sirolimus-steroid. At 12 months, there was no difference in graft survival or patient survival, despite the fact that the rates of biopsy-proven acute rejection (BPAR) post-randomization (4.2% vs. 9.8%) were significantly different between treatments in favor of the CNI-containing regimen. Conversely, GFR and blood pressure significantly improved when CsA had been withdrawn. In long-term follow up CNI free therapy with sirolimus even resulted in better renal function, less graft failures and a lower incidence of malignancies compared to combination therapy of sirolimus and CsA (Oberbauer 2003, Kreis 2004, Russ 2005, Campistol 2006). This study suggests that CsA can be discontinued 3 months after transplantation under immunosuppressive therapy with the mTOR inhibitor sirolimus. Interstingly, the aggravated nephrotoxicity seen under the combination therapy of CsA and sirolimus resulted in inferior outcomes despite lower rejection rates, suggesting that a reduced nephrotoxicity is of greater importance for a favorable long-term outcome compared to a slightly elevated rejection rate (Oberbauer 2005, Mulay 2005).

In chronic renal transplant patients several studies showed beneficial effects on renal function, the feasibility and safety of CNI withdrawal and introduction of SRL for various reasons and in heterogenous groups of patients (Budde and Glander, 2008, Diekmann et al. 2006, Citterlo et al., 2003; Diekmann et al., 2004; Dominguez et al., 2000; Egidi et al., 2003). Diekmann et al described a baseline proteinuria > 800mg/die as the most important prognostic factor for the success of CNI withdrawal.

As demonstrated in small randomized trials in primary kidney transplant patients (Flechner et al., 2002, 2004, Kreis et al. 2000), sirolimus in combination with MMF was equally effective as cyclosporine with respect to percentages of 1- and 2-year patient survival, graft survival, and BPAR rates. In the first pilot study, Kreis had suggested that sirolimus in combination with MMF can be used as primary therapy for the prevention of acute rejection. With similar immunosuppressive efficacy of sirolimus compared to CsA, they observed better renal function, a surrogate marker for long-term outcome (Hariharan et al., 2004). Similarly, Flechner observed in his small study, that Sirolimus treated patients had similar rejection rates, but compared to CsA better renal function, a diminished prevalence of chronic allograft nephropathy (CAN) and down-regulated expression of genes responsible for progression of CAN (Flechner 2004). It was thought that these factors may provide the basis for an alternative natural history with improved long-term graft survival. A recent large and adequately powered study (Ekberg et al. 2007b) however did not confirm the results of this small proof-of concept studies, as CNI replacement by sirolimus in de novo renal allograft recipients was associated to inferior outcomes. Patients randomized to the CNI free treatment arm (Basiliximab+sirolimus+MMF+steroids) had significantly more rejections, inferior renal function, worse graft survival and significantly more side effects, mainly in the early postoperative period. From this study it must be concluded that sirolimus can not replace CNIs in de novo patients.

As pointed out, an important problem of sirolimus treatment in the initial postoperative period is the increased frequency of wound healing problems and lymphoceles (Kahan 2004; Giessing and Budde, 2003, Valente et al., 2003, Goel et al., 2004). It has been suggested that high initial exposure e.g. by the use of a loading dose, which is needed for adequate acute rejection prophylaxis, is associated with this problem (Mehrabi et al. 2006). The effective inhibition of the mTOR complex by sirolimus also leads to inhibition of the growth factor induced proliferation of fibroblasts, which is needed for normal wound healing. As a consequence, many transplant surgeons try to avoid sirolimus in the initial postoperative period and use alternative immunosuppressive regimens, which interfere with wound healing to a much lesser extent.

**Everolimus (Certican®**, RAD, [40-O-[2-hydroxyethyl]-rapamycin], SDZ RAD, RAD001) is a derivative of sirolimus, a macrocyclic lactone, originally isolated from Streptomyces hygroscopicus. Everolimus is more hydrophylic, exhibits a shorter elimination half-life ($t_{1/2}$ 28 ± 7 h), and demonstrates greater relative bioavailability compared with sirolimus (Dunn et al. 2006, Neumayer 2005, Pascual et al. 2006). Following oral administration, everolimus is rapidly absorbed with a median time to peak concentration of 1 hour after single doses in healthy subjects and 2 hours after multiple doses in transplant patients. Both $C_{max}$ and AUC are dose-proportional over the range tested in organ transplantation of 0.5 to 2 mg bid. In 24 healthy volunteers, a high-fat breakfast (44.5 g fat) altered the bioavailability characteristics of everolimus. Compared with a fasting administration, a 60% decrease in peak blood concentration ($C_{max}$), a median 1.3-hour delay in $t_{max}$, and a 16% decrease in total exposure (AUC) was observed. To minimize variability in exposure, it is recommended that everolimus be taken consistently either with food or without (Kovarik et al., 2002b). While Tacrolimus has only a minor influence on everolimus pharmacokinetics (Kovarik et al. 2006, Arns et al. 2006) CsA leads to a 2-3-fold increase of everolimus exposure (Dunn C, Croom KF. Everolimus: a review of its use in renal and cardiac transplantation. Drugs 66:547-70; 2006). In a study with healthy volunteers, Kovarik and colleagues (2002a) found that simultaneous administration of Sandimmun® Optoral (175 mg) with 2 mg everolimus increased everolimus Cmax and AUC by 82% and 168%, respectively (p = 0.0001). Coadministration of 300 mg Sandimmun® Optoral with everolimus did not affect everolimus Cmax (p = 0.59) but increased everolimus AUC by 74% on average (p = 0.0001). From these data it is concluded that if CsA (Sandimmun® Optoral or Sandimmun®) is removed from an everolimus-cyclosporine immunosuppressive regimen, a two- to three-fold decrease in everolimus exposure might be expected. Budde and co-workers (Budde et al. 2006) confirmed this in a prospective pharmacokinetic study in 15 stable maintenance renal allograft recipients on CsA. CsA withdrawal had a marked impact on everolimus pharmacokinetics: Everolimus trough level (C0) was 33% and exposure was 36% lower. In order to obtain similar trough levels after CsA withdrawal a significant higher everolimus dose was necessary (3.9 vs. 2.5 mg/d; p<0.001). The ratio of everolimus dose to C0 as an estimate of apparent clearance increased significantly from 0.4 with CsA (reduced by 50%) to 0.69 l/24h after complete CsA

withdrawal ($p<0.001$). In summary, much lower everolimus doses are needed, when everolimus is used in combination with CsA, and in most patients a daily dose of 1.5mg is sufficient to reach the proposed target range. However, after CsA withdrawal higher doses (around 3-4mg/d) are needed in order to stay within the therapeutic window.

Whole blood trough everolimus concentrations were significantly correlated ($r = 0.86$) with AUC. Upon repeated bid administration in a multiple-dose study, everolimus AUC increased approx. 2- to 3-fold over the initial 4 days of therapy, at which time steady-state was reached. The mean ± SEM apparent total body clearance of everolimus was 8.8 ± 0.2 L/h and the mean ± SD elimination half-life was 28 ± 7 h. Everolimus dosed bid with CsA yields steady-state drug exposure by Day 4 after initiation of therapy in renal and heart transplant recipients. Thereafter, drug blood levels remain relatively constant over time through the first year post-transplant. Demographic characteristics such as age, weight and sex do not influence everolimus metabolism, nor does post-transplant renal impairment (Budde et al., 2006; Certican® IB 2003, p. 25; Neumayer et al., 1999).

The most common side effects associated with everolimus are hematological side effects and hyperlipidemia, most notably leucopenia and hypercholesteremia, which each occurred in >10% of patients (Dunn et al. 2006, Neumayer 2005, Pascual et al. 2006). Other frequent side effects are anemia, thrombocytopenia, edema, gastrointestinal symptoms, and wound healing problems including lymphoceles. From Certican® trials it became evident that there is an as of yet unexplained pharmacodynamic (PD) interaction between Certican® and Sandimmun® Optoral, resulting in enhanced renal toxicity of Sandimmun® Optoral (Eisen, et al 2003). This interaction was first seen with the combination of sirolimus (rapamycin, Rapamune®) and both calcineurin inhibitors (MacDonald, et al 2001, Podder, et al 2001). Similar to sirolimus, everolimus seems to aggravate CsA toxicity (e.g. renal toxicity, hypertension or hemolytic uremic syndrome), when given in combination therapy with CsA. As a consequence, many everolimus associated side effects including hyperlipidemia and exacerbation of CsA-induced nephrotoxicity, can be suppressed with a low incidence of acute rejection when everolimus is applied in combination with reduced CsA doses (Formica et al., 2004). Infectious complications and malignancies were similar to the comparator regimens.

As pointed out, from registration trials it became apparent, that patients on everolimus and CsA had worse renal function compared to patients treated with MMF and CsA (Dunn 2006, Neumayer 2005, Pascual et al. 2006, Vitko et al. 2005, Lorber et al. 2005). Subsequent parallel-group open-label studies (Studies 2306, 2307) aimed at lower CsA exposure in combination with everolimus and provided good evidence that this combination results in excellent outcomes with an acceptable renal function (Vitko et al 2004). Concentration-controlled everolimus (1.5 vs. 3 mg/day) with low exposure CsA provided effective protection against rejection with good renal function (Dunn et al. 2006, Vitko et al. 2004). The incidence of BPAR was significantly higher in patients with a Certican® (everolimus) trough less than 3 ng/mL. Reduced Sandimmun® Optoral exposure did not result in an increased risk of

rejection, and in addition led to improvements in other cyclosporine induced side effects such as hypertension. Surprisingly, higher CsA exposure was even associated with more biopsy-proven rejections and worse long-term outcome (Nashan et al. 2004). However, similar to sirolimus, a higher frequency of lymphoceles and wound healing problems were observed in everolimus treated patients.

Due to the aggravated nephrotoxicity lower CNI levels are therefore strongly recommended for the combination therapy with everolimus, even early after transplantation. In general, CNI levels should be reduced approximately by 50%, however the lower threshold in maintenance patients has not clearly been defined yet (Pascual et al. 2006). In summary, low dose CNI therapy in combination with Certican® has a proven record of safety and efficacy.

Certican® has also demonstrated a potential to reduce the risk factors for the development of chronic rejection. In Phase III heart transplantation studies, the use of Certican® in a subset of patients demonstrated a significant reduction in the occurrence of chronic allograft vasculopathy, a manifestation of chronic rejection and a leading cause of late graft loss (Eisen et al., 2003).

Until now, no therapy has been recognized as being efficient in the prevention of long-term allograft loss due to chronic allograft dysfunction (CAD) reflecting an unmet medical need in transplantation. CNI toxicity is the most common identifiable factor contributing to CAD affecting 18% of patients with failing grafts (Solez et al., 1998; McKane et al. 2001; Nankivell et al., 2001). In addition, CNI-induced renal insufficiency of native kidneys is an important problem in other solid organ transplant recipients (Ojo et al., 2003; Jain et al. 1999; Satchithananda et al., 2002; van Gelder et al. 1998). Therefore, the use of antiproliferative drugs in combination with the withdrawal of CNI has become an interesting concept in treating calcineurin inhibitor toxicity leading to chronic slow deterioration of renal allograft function. However, the association between renal function 1 year after transplantation and long-term outcome (Hariharan et al., 2004) suggests that rather the prevention of CNI induced toxicity and not the treatment of established nephrotoxicity is the ultimate goal in transplant medicine.

In recent studies whereby the use of CNIs was avoided in the initial immunosuppressive regimen and CNIs were replaced by MMF, an increase in the incidence of acute rejection rates was demonstrated (Vincenti et al., 2001). In initial small pilot studies , in which sirolimus was used instead of CNIs the rejection rates were comparable (Flechner et al. 2002, 2004, Kreis et al. 2000), however the most recent large SYMPHONY study (Ekberg et al. 2007b) demonstrated increased frequency of rejection with subsequent worse outcome. In addition wound healing problems and lymphoceles are an important problem, when mTOR inhibitors are administered in the early postoperative period leading to inacceptable high withdrawal rates from mTOR containing regimens (Kuypers 2005).

However, the higher incidence of co-morbidities in older patients combined with age-related physiological changes and age-related alterations in immunocompetence

present a challenge to physicians to plan therapeutic regimens that optimize immunosuppression and are tolerable in older transplant patients (Martins et al. 2005). Whether older patients need less immuosuppression than younger patients to achieve similar outcomes remains unclear. While progressively decreasing relative risks of acute rejection were reported in one retrospective study of older patients who received organs from donors of any age (Meier-Kriesche et al. 2000), higher rejection rates were reported in two retrospective studies of older recipients receiving organs from older donors (Frei et al 2008; Fritsche et al. 2003).

Optimization of both immunosuppression and tolerability without compromising efficacy may be achieved in older patients by rapid tapering of prednisone to low maintenance doses or using regimens that avoid steroids (Meier-Kriesche et al. 2000; Marcén et al 2006). In comparison to younger patients, older transplant recipients are more susceptible to serious infections, cardiovascular complications and corticosteroid-induced diabetes mellitus. Better efficacy outcomes might be obtained when tacrolimus rather than cyclosporine mircroemulsion (CSA-ME) is used as the cornerstone agent in reduced steroid regimens. For example, corticosteroid-free immunosuppression with tacrolimus was as efficacious in preventing acute rejection as a regimen with standard steroids (Rostaing et al. 2005) while efficacy outcomes were less favorable when CSA-ME was administered without steroids or with minimal steroids compared with a CSA-ME standard-steroid regimen in a prospective randomized study of standard risk patients (Vincenti et al. 2008). While tacrolimus may provide slightly better rejection prophylaxis, there is no doubt that tacrolimus containing regimens favor the development of Diabetes and Polyoma nephropathy. Especially the development of post-transplant diabetes is related to tacrolimus use and age. (Kasiske et al., 2003). In a prospective randomized study comparing CsA versus tacrolimus (Vincenti et al. 2007), 35% of patients above the age of 50 developed post-transplat diabetes with CsA compared to 48% of tacrolimus treated elderly patients. Because the development of post-transplant diabetes is a negative predictor for long-term outcome, future regimens for elderly patients need to avoid this complication by avoiding prodiabetogenic immunosuppression such as tacrolimus and/or steroids. In summary, immunosuppresion for elderly recipients should take specific age-related side effects into account, while providing time dependant optimal immunosuppressive rejection prophylaxis.

Recently, many transplant centers have expanded their donor pool to include organs from older donors. Since the initiation of the Eurotransplant Senior Program, older organs are frequently used for older recipients. Despite the risks inherent in using older donors, favorable clinical outcomes have been reported in age-matched transplant populations (Frei et al. 2008; Emparan et al 2004; Arbogast H et al. 2005). These organs are more prone to develop ischemia and reperfusion injury and are more susceptible to calcineurin-induced nephrotoxicity. For example a higher incidence of early phase dialysis was reported in recipients of organs from older donors in a non-comparative study (Osuna A et al. 2005). To balance the potential negative effects of using marginal donor organs (for example, a greater risk of delayed graft function [DGF] and increased susceptibility to calcineurin inhibitor induced nephrotoxicity), induction therapy with basiliximab has been evaluated in

clinical studies with favorable results (Fabrizii et al. 2005, Hong et al. 1999, Emparan et al. 2004). It has been shown that Basiliximab, a chimeric monoclonal antibody directed to the $\alpha$-chain of the interleukin-2 receptor, expressed on the surface of activated T lymphocytes, significantly reduced the incidence of acute rejection (Nashan et al., 1997; Kahan et al., 1999, Ponticelli et al., 2001). Therefore, patients received in addition to an immunosuppressive therapy of cyclosporine and corticosteroids with or without azathioprine a concomitant medication of 20mg basiliximab by i.v. infusion preoperatively and 20mg on day 4 after transplantation. With this application scheme of 40mg basiliximab an adequate receptor saturation defined as a serum concentration more than 0.2 µg/ml was maintained for 36 ± 14 days and a basiliximab clearance of 36.7 ± 15.2 ml/hr was observed (Kovarik et al., 1999). Interesingly, Kovarik and co-workers revealed a significantly lower clearance of basiliximab in patients treated with triple immunosuppression consisting of cyclosporine, corticosteroids and mycophenolate mofetil resulting in a prolonged duration of CD25 saturation (59 ± 17 days) compared with a dual therapy (Kovarik et al., 2001) No data is available concerning the influence of mycophenolic acid on a single dose of 20mg basiliximab but it can be assumed that the period of IL-2 receptor blockade of 20 ± 7 days in patients receiving only one injection of basiliximab is extended under concomitant administration of mycophenolic acid (Kovarik et al., 1999). Nevertheless, the induction therapy with IL-2R antibodies has become the standard of care in many centers because they allow for better rejection prophylaxis despite lower CNI levels, thereby substantially reducing the nephrotoxic potential of the immunosuppressive regimen (Ekberg et al. 2007). While the reduction of the potential nephrotoxic CNIs is performed in many centers under the umbrella of induction therapy e.g. with IL-2R antibodies, other centers prefer the complete avoidance of CNIs or the delayed administration of CNIs in order to prevent the development of DGF. As pointed out, the complete avoidance of CNIs was not successful in large prospective randomized trials (Ekberg et al. 2007). Similarly, the delayed administration of the potentially nephrotoxic CNIs provided no benefit on incidence of DGF in patients at risk of developing DGF (Kamar et al. 2006). Importantly, delayed administration of CsA was not associated with better graft function, but resulted (although not significantly) in a higher incidence of acute rejection (15,5% vs. 26,5%). The lack of benefit for a delayed introduction of CNIs was confirmed in two additional prospective randomized studies conducted on adult patients (Mourad G et al. 2001, Andres et al. ESOT 2007 Senior study). In summary, the delayed use of CNIs is not advantageous, and even may predispose to higher rejection rates, especially in the highly mismatched participants of the ESP Program (Fritsche et al. 2003). Because restoration of renal function in organs from older donors is important, and because these organs tend to be more fragile and more susceptible to the long-term consequences of the nephrotoxic effects of calcineurin inhibitors (Meier-Kriesche et al. 2000) an early conversion to a CNI-free regimen may be adisible, because all attempts to delay or avoid CNIs were not successful yet.

To date, only a handful of randomized clinical studies have prospectively evaluated age-adapted immunosuppressive regimens for older kidney transplant recipients. As pointed out such regimens have to account the fragility of the older organs and have

to take the higher age related morbidity, especially cardiovascular morbidity, into account. Renal allograft recipients have per se a 10-fold higher risk of death from cardiovascular disease compared to the general population due to immunosuppressive therapy (Kasiske, 2000; Wheeler et al., 2000; Miller, 2002) and it is not surprising that elderly renal transplant patients exhibit a greater mortality (Doyle et al, 2000). Whereas the immunosuppressive therapy with CNIs is associated with an increased cardiovascular risk, it might be beneficial for the cardiovascular system to switch immunosuppression to an mTOR inhibitor based therapy. Paoletti and colleagues investigated in renal transplant recipients the effect of sirolimus on left ventricular hypertrophy (LVH) – a powerful risk factor for cardiovascular death. Conversion from CNI to sirolimus was associated with a regression of LVH after kidney transplantation (Paoletti et al., 2008). Similar results were reported in a study which evaluated the impact of an early switch of the immunosuppressive therapy from CsA to everolimus (Seckinger et al., 2008). The prolonged treatment with CsA patients led to a significant increase of pulse wave velocity, a well-known correlate for arterial stiffness and as well a predictor of cardiovascular mortality. Interestingly, conversion to everolimus resulted in a stable pulse wave velocity.

Until now, combination therapy of CNIs with MPA and steroids remains the standard for the early postoperative period. As elderly patients are more prone to develop diabetes mellitus, and steroid associated complications, early steroid withdrawal seems important. Because tacrolimus also favors the development of post transplant diabetes mellitus, especially in elderly patients, CsA should be preferable in this population. However, it seems to be slightly less potent (Webster et al. 2005, Vincenti et al. 2007, Ekberg et al. 2007b), which could be due to the lower MPA exposure observed with CsA. One logical approach therefore would be to adequately increase MPA exposure in order to increase the potency of a CsA-based regimen, without increasing the risk for post transplant diabetes mellitus. Because the long-term nephrotoxic effects of CNIs however may jeopardize the elderly graft, an early CsA withdrawal may be crucial for improving renal function and long-term outcomes. Thus a new immunosuppressive approach utilizes the well known and effective CNIs during the initial post-transplant period, with subsequent CNI elimination. In this way, the initial therapeutic actions are directed towards a protective effect against acute rejection during the early critical period, with a later focus on CNI withdrawal in order to avoid long-term nephrotoxicity. Several studies in de novo renal transplant recipients who underwent CNI withdrawal after 3-6 months while receiving mTORi or MPA showed good graft and patient survival rates with an improved renal function in most studies (Velosa et al., 2001, Oberbauer et al. 2005, Mulay et al. 2005, Smak Gregoor et al., 2002; Vincenti et al., 2001; Abramowicz et al., 2002, Ekberg et al., 2007a). However, all studies reported an increased incidence of acute rejection rates. In order to reduce the risk of rejection the combination of mTORi plus MPA may overcome the increased rejection rates, and may allow for a more timely and safe CNI withdrawal. Because the combination therapy of MMF and sirolimus in de novo patients is safe and as effective as CsA in combination with MMF, this combination may reduce the risk of rejection further to normal levels. In the search for new potent therapeutic strategies virtually getting along without long-term CsA application, it

appears quite compelling to utilize possibly synergistic effects of Myfortic® and Certican®. The pharmacokinetic properties as well as clinical safety and efficacy of Myfortic® and Certican® have been adequately documented separately for each drug in clinical trials. Over the last years two smaller conversion studies to a CNI-free regimen in renal maintenance patients have been performed with Certican®, without noting any rejection when used in combination with Myfortic® (Budde et al. 2006, Arns et al. 2006). It is important to point out that when conversion was done over a prolonged period (e.g. more than 4 weeks) in maintenance patients on Myfortic® doses > 720mg/d, even signs of overimmunosuppression (e.g. infections) were observed providing further evidence that the risk of rejection is clearly lower under Certican® with adjunct MPA therapy. Similarly, combination therapy of MMF and sirolimus has been explored in several clinical trials and is considered as a safe and promising option to avoid the nephrotoxic side effects of CNIs (Olyaei AJ et al., 2001; Flechner et al., 2002; Flechner 2004; Kreis et al., 2004).

For this purpose, a sequential study design may further reduce the drug specific side effects, while maintaining excellent efficacy. The design chosen for this study was also used in other studies (Johnson et al., 2001; Flechner et al., 2002; Vitko et al 2004). So far, the preliminary results of three studies confirm the safety and efficacy of this sequential approach with an early (3-6 months) conversion from CNI to mTORi, resulting in significantly improved renal function (Pearson et al., ATC 2008, Budde et al., ICTS 2008). Because elderly kidneys from elderly donors are more susceptible to long-term nephrotoxic effects of CNIs an earlier conversion seems necessary for patients in the ESP program. The extended use of the well tolerated IL-2R antibodies therefore may provide a safe and efficacious anti-rejection prophylaxis, allowing an early CsA conversion to Everolimus. However, long-term data of these new regimens are lacking and no study of any early CNI-free strategy (either withdrawal or avoidance) has shown a positive impact on long-term outcome compared to the standard immunosuppressive regimen with CNI and MPA.

A CNI-free regimen with Certican® and Myfortic® under the umbrella of the extended use of the IL-2R Ab Simulect will be compared with a standard Sandimmun® Optoral based immunosuppressive regimen. It is expected, that the Sandimmun® Optoral-free regimen will result in a superior renal function, while the experimental arm is as safe and effective as the Sandimmun® Optoral-based regimen with regard to occurrence of biopsy proven acute rejection episodes, graft loss, and death. The current study therefore aims to compare two different treatment regimens rather than individual drugs. Assuming a similar overall efficacy and safety of both regimens, the frequency and occurance of specific treatment emergent side effects are of special interest. The identification of such side effects and sub-populations, which are at risk for these side effects could allow a more individualized treatment approach in the future.

# 2 Study purpose

This study wants to address the following question: Is a CNI-free regimen six weeks after transplantation for ESP patients as safe and well tolerated as standard treatment optimised for immunosuppression (benefit in renal function, NODM, CV risk, cancer, allograft nephropathy)?

# 3 Objectives

The primary objective of this trial is to demonstrate superiority of a treatment regimen with Certican with respect to the renal function at 6 months after renal transplantation (Tx) assessed by glomerular filtration rate (GFR) – Cockcroft-Gault method - as compared to the standard regimen in *de novo* senior renal transplant recipients.

Secondary objectives of this trial are:

- to assess renal function by GFR – MDRD and Nankivell method – at Month 6 post Tx
- to assess renal function by serum creatinine at Month 6 post Tx
- to assess efficacy (biopsy proven acute rejection, graft loss, death) at Month 6
- to assess occurrence of treatment failures up to or at Month 6, while treatment failure is defined as a composite endpoint of biopsy proven acute rejection, graft loss, death, loss to follow up and discontinuations due to lack of efficacy or toxicity, or conversion to another regimen (at least one condition must be present)
- to assess evolution of renal function (creatinine slope) between Week 7 (randomization) and Month 6
- to assess CD25 saturation on lymphocytes
- to assess safety and tolerability at Month 6:
  - AE/SAE
  - incidence of infections (CMV, BK virus)
  - tumor incidence
  - cardiovascular risk (Framingham Score, ECG)
  - occurrence of proteinuria
  - NODM

# 4 Study design

This is a prospective, multi-center, randomized, controlled, open label study in *de novo* senior renal transplant recipients. Approximately 240-260 patients fulfilling the inclusion and exclusion criteria will be randomized in a 1:2 fashion to either Control or Certican arm.

Once consent is obtained, baseline data will be collected to determine each patient's eligibility for study participation. Baseline (BL) assessment will be performed prior to transplantation.

For the first six weeks post transplantation, all study patients will receive induction therapy with Simulect® (2 x 20 mg (day 0 [2 hrs prior to Tx] and day 4 post Tx) and will commence on an immunosuppressive regimen consisting of Myfortic® "loading dose" + Sandimmun® Optoral (based on C0-h level) with corticosteroids (steroid withdrawal after week 2).

At BL2 (Week 7, Visit 3), patients whose eligibility is confirmed by additional in- and exclusion criteria will be randomized and hence allocated to one of the two treatment groups in a 2:1 ratio.

**Group 1: Certican® switch immunosuppressive therapy in the following steps**

Step 1: Day 1 morning: Myfortic® + Sandimmun® Optoral
Day 1 evening: Myfortic® + Sandimmun® Optoral + Certican® (3 mg)

Step 2: Day 2 morning: Myfortic® + Certican® (1.5 mg)
Day 2 evening: Myfortic® + Certican® (1.5 mg)

Step 3: Day 3 morning: Myfortic® + Certican® (TL 5 – 10 ng/mL)

**Group 2: Control** **maintain prior immunosuppressive regimen consisting of** Myfortic® + Sandimmun® Optoral

In group 1, step 1 of the therapy switch will be performed the day after randomization and will be continued on Day 2 with step 2. The therapy switch will be completed on Day 3 with step 3.

Patients randomized to Certican group will receive two additional doses of Simulect® (each 20 mg) as rejection prophylaxis on Week 7 and 12.

The established treatment will be continued until Month 6 (final assessment of the core trial). Control assessments will be performed at Week 12, 18 and 24 (end of study or early discontinuation) post Tx. Renal function (creatinine, GFR) as well as safety and efficacy measures will be assessed at every study visit.

In the event of suspected acute rejections, renal biopsies will be performed.

Additionally, on day 7 ± 2 after randomization a blood level control will be performed.

Statistical analysis and report writing will be performed when all patients have completed Month 6 after Tx assessment (or discontinued the study prematurely).

An independent Safety-Monitoring-Board will review the safety data every 3 months during the recruitment phase and every 6 months thereafter, until all patients have completed the study treatment (Month 6).

### Table 4-1 Study outline

| Phase | Prephase | | Study Drug Treatment | | | | |
|---|---|---|---|---|---|---|---|
| Period | BL1 | | BL2 | Maintenance | | | |
| Visit | 1 | 2 | 3 | 4 | 5 | 6 | 7 |
| Week | 0 | 2 | 7 | 8 | 12 | 18 | 24 |
| Month | | | | | | | 6 |
| Random. | | | X | | | | |

| Treatments | | | | | | | |
|---|---|---|---|---|---|---|---|
| **Certican group** | | | | | | | |
| - Simulect® | 20 mg day 0 (2hrs prior Tx) day 4 post Tx | none | 20 mg (week 7) | none | 20 mg (week 12) | | |
| - Myfortic® 2) | 2880 mg/day | 2160 mg/day | 1440 mg/day | | | | |
| - Sandimmun® Optoral 1) | acc. to blood level | acc. to blood level | none | | | | |
| - Corticosteroids | 5 mg/day (minimum dose) | removal | none | | | | |
| - Certican® | None | none | introduction | acc. to blood level (5-10 ng/ml) | | | |
| **Control group** | | | | | | | |
| - Simulect® | 20 mg day 0 (2hrs prior Tx) day 4 post Tx | none | none | | | | |
| - Myfortic® 2) | 2880 mg/day | 2160 mg/day | 1440 mg/day | | | | |
| - Sandimmun® Optoral 1) | acc. to blood level * | removal | Acc. to blood level (see below) | | | | |
| - Corticosteroids | 5 mg/day (minimum dose) | removal | none | | | | |
| - Certican® | None | none | none | | | | |

BL1 = Baseline visit 1: is to be performed prior to transplantation

BL2 = Baseline visit 2: day of randomization

Dosages: The given total daily dosages of the immunosuppressive drugs (Myfortic®, Sandimmun® Optoral, Certican®) should be divided into 2 (equal) doses, applied 12 hours apart (i.e., 08:00 a.m. and 08:00 p.m.)

1) Optoral® Dosage:

| | |
|---|---|
| Week 1 – Week 4: | C0-h 150 – 200 ng/ml |
| Week 5 – Week 18: | C0-h 100 – 150 ng/ml |
| Week 18 – Week 24: | C0-h 80 – 120 ng/ml |

(cf. Section 6.5.2)

2) Myfortic® Dose reductions to are possible in case of tolerability problems (cf. Section 6.5.2)

3) Switch: Introduction of Certican® and removal of Sandimmun® Optoral will be performed as described in Section 6.5.1.

Patients should be seen for all visits on the designated day or as close to it as possible.

Baseline visit 2 assessments and Randomization (Visit 3, Week 7) may be performed in a time window of Week 7 and week 8.

# 5 Population

The study population will consist of a representative group of approximately 240-260 *de novo* senior kidney transplant patients participating at the Eurotransplant senior program (ESP). These patients will be randomized at Week 7 to either the Control arm or the Certican arm in a 1:2 ratio; 82 patients in the control arm, 164 patients in Certican arm. Enrollment is planned for 24 months, but will be continued until the required sample size will be achieved. The patients will be recruited from about 8 – 12 transplant centers in Germany.

### Inclusion / Exclusion criteria for Baseline visit (day of transplantation)

The investigator must ensure that all patients who meet the following inclusion and no exclusion criteria are offered enrollment in the study. No additional exclusions can be applied by the investigator, in order that the study population will be representative of all eligible patients.

### <u>Inclusion Criteria</u>

The following inclusion criteria must be present at Baseline visit 1 (BL1, Screening visit prior to transplantation

1. Males or females, aged > 65 years, receiving the first kidney from a donor aged > 65 years in the Eurotransplant Senior Program
2. Recipients of de novo cadaveric kidney transplants

3. Patients who are willing and able to participate in the study and from whom written informed consent has been obtained

**Exclusion Criteria**

The following exclusion criteria must not be present at Baseline visit 1 (BL1, Screening visit prior to transplantation)

1. Multi-organ recipients (e.g., kidney and pancreas)
2. Patients receiving a kidney from a non-heart beating donor
3. Patients who are recipients of A-B-0 incompatible transplants
4. Patients with a historical or current peak PRA of > 25% (current = 3 months)
5. Patients with already existing antibodies against the HLA-type of the receiving transplant
6. Patients with any known hypersensitivity to Simulect®, Certican®, mycophenolic acid, cyclosporine A, other drugs similar to Certican® (e.g., macrolides), or other components of the formulations (e.g. lactose)
7. Patients who have received an investigational immunosuppressive drug within four weeks prior to study entry (Baseline visit 1)
8. Patients with thrombocytopenia (platelets < 75,000/mm$^3$), with an absolute neutrophil count of < 1,500/mm$^3$ or leucopenia (leucocytes < 2,500/mm$^3$), or hemoglobin < 6 g/dL
9. Patients with symptoms of significant somatic or mental illness. Inability to cooperate or communicate with the investigator, who are unlikely to comply with the study requirements, or who are unable to give informed consent
10. Patients who are HIV, HCV RNA, or Hepatitis B surface antigen positive
11. Evidence of severe liver disease (incl. abnormal liver enzyme profile, i.e. AST, ALT or total bilirubin > 3 times UNL)
12. Females at randomization who will be not considered post-menopausal according to the following definition:

    = 12 months of natural (spontaneous) amenorrhea <u>or</u>

    = 12 month post surgical bilateral oophorectomy <u>or</u>

    hysterectomized women over or equal to 65 years of age
13. Presence of a clinically significant infection requiring continued therapy, severe diarrhea, active peptic ulcer disease, or uncontrolled diabetes mellitus that in the opinion of the investigator would interfere with the appropriate conduct of the study
14. History of malignancy of any organ system, treated or untreated, within the past 5 years whether or not there is evidence of local recurrence or metastases, with the exception of localized basal cell carcinoma of the skin
15. Evidence of drug or alcohol abuse
16. Patients receiving drugs known to strongly interact with CsA and/or everolimus according to the list provided in Appendix 3 to this protocol should be excluded, if in the opinion of the investigator this drug interaction interferes with the objectives

of the study, namely a clinical meaningful potentiation of renal dysfunction and/or maintenance of adequate immunosuppressive drug levels

**Inclusion / Exclusion criteria for randomization (Baseline visit 2)**

Of all patients included into the study at BL1 (prior to transplantation), those can continue into the randomized study period, in whom the following condition is observed at BL2, prior to randomization.

**Inclusion Criteria**

1. Patients with an stable serum creatinine < 3.0 mg/dl
2. Patients have to be on an immunosuppressive regimen with ≥ 720 mg/d Myfortic® and Sandimmun® Optoral
3. Patients with a proteinuria < 500 mg/g creatinine

**Exclusion Criteria**

Of all patients included into the study at BL1 (prior to transplantation), those can not continue into the randomized study period, in whom one of the following criteria are met at BL2, prior to randomization:

1. Graft loss after current transplantation (since Baseline visit 1)
2. Patients who received a depleting antibody therapy or more than 10mg/day prednisolon (or an equivalent oral steroid therapy)
3. Patients who suffered from rejection (more than BANFF 1A), recurrent acute rejection, or steroid resistant acute rejection
4. Patients with thrombocytopenia (platelets < 75,000/$mm^3$), with an absolute neutrophil count of < 1,500/$mm^3$ or leucopenia (leucocytes < 2,500/$mm^3$), or hemoglobin < 6 g/dL
5. Evidence of severe liver disease (incl. abnormal liver enzyme profile, i.e. AST, ALT or total bilirubin > 3 times ULN)
6. Current dialysis dependency at Baseline visit 2
7. Patients with clinically significant infection requiring continued therapy which would interfere with the objectives of the study
8. Presence of intractable immunosuppressant complications or side effects (e.g., severe gastrointestinal adverse events) at randomization visit (Baseline visit 2)

# 6 Treatment

## 6.1 Components of Investigational therapies

**Investigational drug**

- **Certican®**
  Active ingredient: Everolimus (RAD001)
  Galenic form: Tablets
  Dose: one tablet containing 0.5 mg,0.75 mg or 1.0 mg
  Dosing schedule: initially 3 mg on day 1 and 2 x 1.5 mg on day 2 after randomization
  afterwards based on blood trough level (5 – 10 ng/mL)
  Packaging: Blisters of 10 Tablets

- **Simulect®**
  Active ingredient: Basiliximab
  Galenic form: lyophilisate in vials with ampoules of sterile water for injection (5 mL)
  Dose: one vial containing 20 mg lyophilisate
  Dosing schedule: 2 x 20 mg (Day 0 (2 hrs prior to Tx) and Day 4 post Tx) in Control and Certican group. Additional doses of Simulect (each 20 mg) on Week 7 and 12 in Certican group to be applied as i.v. bolus injection
  Packaging: packages of 1 lyophilisate in vial with ampoules of sterile water

**Other immunosuppressive drugs**

- **Sandimmun® Optoral**
  Active ingredient: Cyclosporine A
  Galenic form: capsules
  Dose: one capsule containing 10, 25, 50, or 100 mg
  Dosing schedule: according to blood level
  Packaging: trade ware will be used

- **Myfortic®**
  Active ingredient: Enteric Coated Mycophenolate Sodium (EC-MPS)
  Galenic form: Tablets
  Dose: one tablet containing 180 mg or 360 mg
  Dosing schedule: “loading dose” regimen: week 1 – 2: 2880 mg/day;
  week 3 – 6: 2160 mg/day; week 7 – 24: 1440 mg/day, if tolerated

dose reduction due to side effects are possible

Packaging: trade ware will be used

- **Corticosteroids**
  according to local standard. Steroid withdrawal after week 2.

## 6.2 Treatment groups

At Baseline visit 2 (Visit 3, Week 7) patients will be assigned to one of the following two treatment groups in a 2:1 ratio:

**Group 1: Certican:** **switch to immunosuppressive regimen consisting of:**
Certican® + Myfortic®

**Group 2: Control:** **maintain prior immunosuppressive regimen consisting of:**
Sandimmun® Optoral + Myfortic®

## 6.3 Treatment assignment

A randomization list will be produced by or under the responsibility of Novartis Biometrics Department using a validated system that automates the random assignment of treatment groups to randomization numbers in the specified 2:1 ratio (Certican®, Control). Two sets of randomization numbers will be prepared for stratified randomization, one set for the group of patients who did not experience any rejection between Baseline 1 and Baseline 2 (Visit 3/Week 7) and one set for the group of patients who did experience at least one rejection in this period. Randomization numbers will be different in the two sets. The randomization scheme will be reviewed and locked after approval. According to the recommendations given in the ICH E9 Guideline “Statistical Principles for Clinical Trials” (CPMP, 1998), the used block length is specified in a separate document which is withhold from the study centers. The randomization list will be kept sealed in a secure location.

At Baseline 1 which has to be performed prior to the transplantation surgery, patients will be assigned a unique patient identification by the investigator. The patient identification has two parts. The first part is the four-digit center number, which is assigned by Novartis. The second part will be assigned by the investigator starting with number “00001” in each study site. Once assigned to a patient, the patient identification will not be reused. If the patient fails to be randomized for any reason, the patient’s identification and the reason for not being randomized will be entered on

the Study Completion Page. Note: The patient identification is different from the randomization number.

At Baseline 2 (visit 3, Week 7), all eligible patients will be given a randomization number that assigns them to one of the treatment groups. The randomization numbers are sequentially allocated to the patients per center in the order of inclusion in the randomized treatment period. Each patient will receive only that treatment regimen which is labeled with the randomization number allocated to him/her.

Randomization of individual patients will be performed centrally by the designated CRO. Allocation of a patient to one of the two treatment groups will be performed in the following steps.

1. Information about patient inclusion (BL 1)
   Study sites will inform the randomization department of the designated CRO about each patient inclusion via fax
2. Reminder (Visit 2/Week 2):
   By a reminder that will be included in the CRF of the Week 2 assessments, site will be reminded to schedule the next Visit 3 (Week 7) on time
3. Randomization (BL 2):
   At day of randomization, the site will provide the patient information of eligible patients to the designated CRO by fax.
   Depending on the stratum (previous rejections yes/no), the designated CRO randomization department will allocate the patient the next free consecutive randomization number allocated to the site per stratum. Randomization number and allocated treatment (according to the randomization list) will be provided to the site by fax.

The randomization number will be entered on the CRF of an individual patient.

## 6.4 Treatment blinding

Not applicable. This is an open-label study.

## 6.5 Treating the patient

### 6.5.1 Study drug administration

Each study site will be supplied by Novartis with study drug (everolimus, Certican®) for patients who are treated in the Certican®-group. Study drug will be provided as tablets, packaged in boxes containing strips of blisters packed tablets, and labeled as RAD001 Tablets. Study drug will be available in 0.75 mg strength. Additional medication for dose titrations will be provided in 0.5 mg and 1.0 mg strength.

One component of the box consists of a 2-part label which includes an identifier of each single box of study medication. Before dispensing the study medication to an

individual patient, investigator staff must enter the 4-digit patient code and the date of dispensing of the medication box on both parts of the label. The outer part of the label will be detached from the packaging and affixed to the source document (Drug Label Form in the CRF) containing that patient's unique patient number.

For the other immunosuppressive drugs (Simulect® induction therapy, Sandimmun® Optoral, Myfortic®, Corticosteroids) the patient will receive a prescription.

Patients randomized to Certican®-group will receive additional doses of Simulect® (each 20 mg) on Week 7 and 12 as rejection prophylaxis. These doses will be provided as investigational drug.

The prescribed total daily dosage of the immunosuppressive drugs should be divided in two (equal) doses, applied 12 hours apart (i.e. 08:00 a.m. and 08:00 p.m.).

The investigator should instruct the patient to take the study drug exactly as prescribed in order to promote compliance. All dosages prescribed and dispensed to the patient and all dose changes during the study must be recorded on the Dosage Administration Record CRF.

In patients randomized to the Certican® regimen, withdrawal of Sandimmun® Optoral and commencement of Certican® will be performed in different steps as described below, starting at Visit 3 (Week 7).

Step 1: Day 1 morning: Myfortic® + Sandimmun® Optoral
Day 1 evening: Myfortic® + Sandimmun® Optoral + Certican® (3 mg)

Step 2: Day 2 morning: Myfortic® + Certican® (1.5 mg)
Day 2 evening: Myfortic® + Certican® (1.5 mg)

Step 3: Day 3 morning: Myfortic® + Certican® (TL 5 – 10 ng/mL)

Dose increase / adjustment of Certican® and Sandimmun® Optoral should be performed based on the investigator's experience and the given therapeutic blood levels, adverse events, etc. Dose adjustments should be monitored 7 days ± 2 later (Visit 4).

Sandimmun® Optoral will be administered according to blood trough levels: week 1 – 4 150 – 200 ng/ml (all patients), week 5 – 6: 100 – 150 ng/ml (all patients), week 7 – 18: 100 – 150 ng/ml (only patients in Control group), week 18 – 24: 80 – 120 ng/ml (only patients in Control group).

Myfortic® will be given in both groups according to the "loading dose" regimen: week 1 – 2: 2880 mg/day; week 3 – 6: 2160 mg/day; week 7 – 24: 1440 mg/day.

Corticosteroids will be added to the immunosuppressive regimen according to local standard but with minimum dose of 5 mg/day. Steroids will be withdrawn after week 2 in both groups.

### 6.5.2 Permitted study drug adjustments

For patients who are unable to tolerate the protocol-specified dosing schedule, dose adjustments are permitted in order to keep the patient on study drug. The recommendations described below should be followed as far as clinically feasible.

**Certican®**

Because Certican® is the mainstay of immunosuppression in the CNI-free group, Certican® dose should be continued on a stable level according to trough levels. Therefore reduction or elimination of Myfortic® should be considered first (step 1) before adjusting the Certican® dose (step 2).

Steroids could be added to the immunosuppressive regimen if necessary (step 3). In case of not achieving sufficient immunosuppression Sandimmun® Optoral could be added to the IS treatment in the Certican®-group (step 4).

Target therapeutic ranges for Certican® (everolimus) whole blood trough levels after CNI-removal should be 5 to 10 ng/mL.

Dose adjustments may be performed if Certican® (everolimus) whole blood trough levels outside the target range are measured. Dose adjustments of Certican® should be monitored 7 days later by a measurement of the everolimus blood trough level.

In both renal and heart transplantation, everolimus trough concentrations ≤ 3 ng/mL were associated with a significantly lower protection from acute rejection compared with trough levels > 3 ng/mL. Although hyperlipidemias were common over the full exposure range, they responded to corticosteroid dose reduction, dietary management, and lipid-lowering therapies, and were, therefore, not dose-limiting. The incidence of leukocytopenia did not show any exposure-response relationship to everolimus trough concentrations. The incidence of notable thrombocytopenia (< 100 x $10^9$/L) increased with Certican® exposure; however, the incidence of clinically meaningful platelet reductions (< 75 x $10^9$/L) was generally low.

When Certican® is used with conventionally dosed CsA, the lower therapeutic concentration was 3 ng/mL (Kovarik et al., 2001, 2002b; Vitko et al., 2004). For safety reasons, therefore, a higher lower limit seems necessary, when CsA is discontinued. This was the rationale to increase the lower limit to 5 ng/mL after withdrawal of Sandimmun® Optoral. A more precise upper therapeutic limit could not be defined. There is, however, limited clinical experience that doses causing concentrations >8 ng/mL may lead to an increased frequency of side effects (Kovarik et al., 2001, 2002b, 2002c; Vitko et al., 2004). Certican® (everolimus) trough levels > 12 ng/mL should be avoided.

If Certican® medication is interrupted for safety related considerations for more than 14 consecutive days within the 6 months study treatment (or cumulative for more than 4 weeks), discontinuation of the medication should be considered and the patient should be withdrawn from the study. Appropriate information needs to be recorded on the Dosage Administration CRF.

## Sandimmun® Optoral

Target therapeutic range for cyclosporine whole blood levels should be the following.

| Group | Certican | Control |
|---|---|---|
| **Timepoint** | **C0-h levels [ng/ml]** | |
| Week 1 – Week 4 | 150 – 200 | 150 – 200 |
| Week 5 – Week 6 | 100 – 150 | 100 – 150 |
| Week 7 – Week 18 | 0 | 100 –150 |
| Week 18 – Week 24 | 0 | 80 – 120 |

Dose may be adjusted if measured cyclosporine whole blood levels are outside the target range. In case of severe CsA toxicitiy, dose reductions below the target levels may be performed on the investigators' discretion.

If Sandimmun® Optoral medication is interrupted for more than 14 consecutive days within the 6 weeks (Certican group), respectively 6 months (Control group) of study treatment (or cumulative for more than 4 weeks), discontinuation of the medication should be considered and the patient should be withdrawn from the study.

In the CNI-free group the Sandimmun® Optoral dosage will be reduced starting at Week 7 and removed completely the day after.

## Myfortic®

In both treatment groups, Myfortic® should be given according to the "loading dose" regimen: 2880 mg/day during week 1 to 2, 2160 mg/day in week 3 to 6 and than maintained at 1440 mg/day in week 7 to 24 , if tolerated.

In case of leukopenia (leukocyte count <2,500/mm$^3$), neutropenia (neutrophil count <1,500/mm$^3$), or anemia, Myfortic® medication doses may be reduced at the discretion of the investigator or eliminated completely until the event resolves. Furthermore, for any other moderate/severe AE which in the opinion of the investigator warrants a dose reduction or temporary interruption of the medication, this will be allowed. However, the Myfortic® medication should be restarted once the AE has resolved or returned to an acceptable grading (see instructions given in Appendix 4).

If Myfortic® medication is interrupted for safety related considerations for more than 2 consecutive weeks or more than 4 cumulative weeks within the 6 months study treatment, discontinuation of the medication should be considered and the patient should be withdrawn from the study. Appropriate information needs to be recorded on the Dosage Administration CRF.

The Month 6 assessments will be performed and documented for all patients who are withdrawn prematurely from the treatment at timepoint of discontinuation.

## Oral corticosteroids

Oral corticosteroids will be given at a minimum dose of 5 mg prednisolon or equivalent during the first two weeks post transplantation. If possible, it should remain stable during these two weeks. However, dose adjustments for medically indicated reasons will be possible.

All immunosuppressive medication, with single dose, unit, frequency, route of administration, indication, start and end dates will be recorded in the Immunosuppressive Therapies CRF. Only approved but no investigational drugs are permitted.

### 6.5.3 Rescue medication

**Treatment for acute rejection episodes**

A core biopsy must be obtained for all suspected cases of acute rejection. Such biopsies are to be performed prior to or within 24 hours after the start of anti-rejection therapy. Whenever possible, anti-rejection therapy should be postponed until a histological diagnosis of rejection is confirmed. These biopsies will be read locally at the center, and assessed according to Banff 07 criteria (see Appendix 6).

Acute rejection should be treated with methylprednisolone intravenously. The recommended dose is 500 – 1000 mg for three days. In cases of Grade III/vascular rejection, the administration of ATG/OKT3 or high doses of i.v. Immunoglobulin is allowed as first line treatment. Plasmapheresis is also allowed in these patients. Patients receiving such therapy may interrupt study medication during that period.

A **steroid-resistant acute rejection** episode will be defined when no stabilization or improvement of creatinine is seen within five days after initiation of methylprednisolone, when the patient has received at least 4 bolus injections with a minimum of 250 mg/bolus, or a total minimum of 1.5 g of methylprednisolone. For such steroid-resistant rejections, antilymphocyte therapy may be initiated at the appropriate dose for 7 – 14 days. However, if there is inadequate response to a full course of steroids and initiation of antilymphocyte therapy is delayed more than 10 days from the biopsy-confirming acute rejection, a repeat biopsy should be performed to confirm ongoing rejection prior to initiating antilymphocyte antibody therapy.

Patients suffering rejection ≤ BANFF 1A should be randomized and treated according to immunosuppressive regimen given in the study protocol.

**Infection prophylaxis**

Prophylactic treatment for Cytomegalovirus (CMV) for high risk patients (a CMV positive donor organ transplanted into a CMV negative recipient) is strongly

recommended. Treatment will be at the investigator's discretion per standard practice of the individual centers.

All patients may receive prophylactic treatment for Pneumocystis Carinii Pneumonia (PCP) with trimethoprim/sulfamethoxazole (e.g. Bactrim® or Cotrim®) according to local practice. Duration and dosage depend on the discretion of the investigator. Aerosolized pentamidine or dapsone may be administered to patients unable to tolerate oral formulations.

**Treatment of hyperlipidemia**

Lipid lowering medications (e.g., fluvastatin, Lescol®) are to be administered for patients with increased LDL-cholesterol and triglyceride levels following the National Cholesterol Educating Program (NCEP) guidelines (Appendix 5). Lipid lowering medications should be maximized prior to reduction of Certican®. If the elevation of cholesterol and/or triglyceride levels persists, the dosing of Certican® will be adjusted following the recommendations in Appendix 4.

When HMG-CoA reductase inhibitors are used concomitantly with gemfibrozil (and probably other fibrates), cyclosporine or erythromycin, myopathy and rhabdomyolysis have been reported. Caution is advised if the use of such combinations is deemed necessary.

### 6.5.4 Other concomitant treatment

The investigator should instruct the patient to notify the study site about any new medications he/she takes after the start of the study treatment. All medications (other than study treatment) and significant non-drug therapies (including physical therapy and blood transfusions) administered after the patient starts study treatment must be listed on the "Concomitant medications/Significant non-drug therapies after start of study drug" CRF. If required for an AE, such concomitant medication should be cross-referenced on the AE CRF under "action taken", appropriately coded for "concomitant medication taken".

Inducers of CYP3A4 (e.g. rifampin, carbamazepine, phenytoin, and barbiturates have been shown or have the potential to decrease exposure to Certican®. If these drugs are necessary, Certican® doses may have to be increased. Potent inhibitors of CYP3A4 such as ketoconazole, itraconazole and fluconazole should be avoided while the patient is on study medication. Certican® has the potential to increase the exposure to terfenadine, astemizole and cisapride; hence these drugs should be avoided. In vitro data suggested that Certican® may have the potential to interact with quinidine, fluoxetine and paroxetine, and alternative medications should be considered. Since the potential for drug interaction with digoxin has not been evaluated, patients on digoxin should have periodic measurement of digoxin levels.

The concomitant administration of nephrotoxic drugs (e.g., aminoglycosides, NSAIDs) and drugs known to interfere with cyclosporine pharmacokinetics (i.e., ketoconazole, erythromycin, phenytoin, barbiturates, carbamazepine, diltiazem, verapamil, rifampin) should be associated with a careful monitoring of renal function

and cyclosporine blood trough levels. In addition, drugs known to interfere with Centican® or Myfortic® should be avoided (see Appendix 3 for possible drug interactions).

### 6.5.5 Study drug discontinuation

In case of a platelet count <75,000/$mm^3$, a leukocyte count <2,500/$mm^3$ or a neutrophil count <1,500/$mm^3$, at the discretion of the investigator, Myfortic® medication dose may be reduced by half, or eliminated completely until the event resolves. In case of dose reduction, Myfortic® should be reduced according to the guidelines given in Appendix 4. In the CNI-free group, Certican® should be continued on a stable dose level and reduced only if the symptoms do not respond to the reduction of the Myfortic® dosage.

In case of elevated cholesterol and/or triglyceride levels, lipid lowering medications should be maximized. If the elevation persists, the dosing of Certican® should be adjusted following the recommendations given in Appendix 4.

Dose reduction may be performed for those patients with other moderate/severe AEs according to the investigators' judgment. Also in these cases, the medication doses can be reduced by half or interrupted completely until event resolves.

If the Myfortic® or Certican® medication is interrupted for more than 2 consecutive weeks or more than 4 cumulative weeks within the 6 months study treatment, discontinuation of the respective medication should be considered and the patient withdrawn from the study.

Patients who discontinue study treatment before completing the 6-month period should be scheduled for a the end of study visit as soon as possible, at which time all of the assessments listed for the end of study visit (Visit 7, Month 6) will be performed.

All patients who discontinue study drug, including those who refuse to return for a final visit, will be contacted for safety evaluations during the 30 days following the last dose of study drug taken.

### 6.5.6 Premature patient withdrawal from study treatment

Patients *may* voluntarily withdraw from study treatment or study treatment may be discontinued at the discretion of the investigator at any time.

Patients *must* be withdrawn prematurely from the study treatment if any of the following occur:

- premature discontinuation of study drug (see Section 6.5.5)
- graft loss

- severe acute rejection (see Section 6.5.3, first line rescue medication) requiring change in baseline immunosuppressive therapy according to this protocol
- occurrence or detection of severe medical disorder jeopardizing the life of the patient in the immediate future

If such premature withdrawal from study treatment occurs, or if the patient fails to return for visits, the investigator must determine the primary reason for a patient's premature withdrawal from the study and record this information on the Study Completion CRF.

Patients *may* be withdrawn from the study treatment prematurely at the investigator's discretion for one of the following reasons:

1. adverse event(s)
2. abnormal laboratory value(s)
3. abnormal test procedure result(s)
4. unsatisfactory therapeutic effect (e.g. severe rejecetion)
5. protocol violation
6. administrative problems
7. patient's condition no longer requires study treatment

For patients who are lost to follow-up, the investigator should show "due diligence" by documenting in the source documents steps taken to contact the patient, e.g., dates of telephone calls, registered letters, etc.

Patients who discontinue participation in the trial for any reason will not be replaced.

All patients who prematurely discontinue study treatment will not be considered as withdrawn from the study and will be asked to attend the end of study visit (at time point of discontinuation).

The End of Study CRF should be completed at month 6 or earlier if the patient can no longer be followed, e.g., due to death, withdrawal of consent, or loss to follow-up.

### 6.5.7 Emergency unblinding of treatment assignment

Not applicable, since this is an open-label trial.

# 7 Visit schedule and assessments

Table 7-1 lists all of the assessments and indicates with an "X" the visits when they are performed. All these obtained data must be supported in the patient's source documentation.

### Table 7-1 Assessment schedule

| Phase | Prephase | | Study Drug Treatment | | | | |
|---|---|---|---|---|---|---|---|
| Period | BL1[1] | | BL2 | Maintenance | | | EOS[2] |
| Visit | 1 | 2 | 3 | 4 | 5 | 6 | 7 |
| Week | 0 | 2 | 7 | 8[3] | 12 | 18 | 24 |
| Month | | | | | | | 6 |
| Informed consent | x | | | | | | |
| Inclusion/ exclusion | x | | x | | | | |
| Randomization | | | x | | | | |
| Demography | x | | | | | | |
| General medical history | x | | | | | | |
| - smoking status | x | | x | | | | x |
| Transplantation information | x | | | | | | |
| Control biopsy[6] | x | | x | | | | x |
| Physical examination | x | | x | | | | x |
| Vital signs | x | x | x | x | x | x | x |
| ECG | x | | x | | | | x |
| Study medication check | | | | | x | x | x |
| Laboratory test: | | | | | | | |
| - Hemat. / Biochemistry | | | | | | | |
| basic program | | x | | x | x | | |
| extended program | x | | | | | x | |
| complete program | | | x | | | | x |
| - NODM | | | x | | | | x |
| - Viral Serology | x | | x | | | | x |
| - Urinalysis / GFR | | x | x | x | x | x | x |
| Biomarker[4] | | | x | | x | | x |

| Phase | Prephase | | Study Drug Treatment | | | | |
|---|---|---|---|---|---|---|---|
| Period | BL1[1] | | BL2 | Maintenance | | | EOS[2] |
| Visit | 1 | 2 | 3 | 4 | 5 | 6 | 7 |
| Week | 0 | 2 | 7 | 8[3] | 12 | 18 | 24 |
| Month | | | | | | | 6 |
| Drug concentration | | | | | | | |
| Sandimmun® Optoral $C_0$ level[5] | | x | x | x | x | x | x |
| Certican® trough level[5] | | | | x | x | x | x |
| Rejection episodes | ←——— as necessary ———→ | | | | | | |
| Renal biopsy | ←——— as necessary ———→ | | | | | | |
| Infections | ←——— as necessary ———→ | | | | | | |
| Tumour | ←——— as necessary ———→ | | | | | | |
| AEs | ←——— as necessary ———→ | | | | | | |
| SAEs | ←——— as necessary ———→ | | | | | | |
| Comments | ←——— as necessary ———→ | | | | | | |
| Concomitant therapy | ←——— as necessary ———→ | | | | | | |
| Immunosuppressive therapy | ←——— as necessary ———→ | | | | | | |
| End of treatment / End of study[2] | | | | | | | x |

BL: Baseline visit 1: prior to transplantation BL2: Baseline visit 2 : day of randomization
EOS: End of Study

1) Visit 1 (BL ) is to be performed prior to transplantation
2) At end of study (Week 24 post Tx) or at the time of early discontinuation
3) Blood level control on Day 7 ± 2 after randomization
4) Optional
5) To be assessed only if Sandimmun® Optoral or Certican® are administered at that visit. Certican®-troughlevels will be determined locally.
6) Optional – if part of center standard procedure – control biopsies may be taken only at indicated time points

Patients should be seen for all visits on the designated day or as close to it as possible. Visit 3 (Week 7 – BL2) may be performed within a time window of week 7 and week 8.

### Visit 1 (day 0 / Week 0) – Baseline visit 1

The Baseline visit 1 assessment is to be performed prior to transplantation, i.e. within 24 hours before surgery.

1. Patient signs informed consent

2. Patient's eligibility for the study according to in- and exclusion criteria will be checked
3. Demographic data will be recorded (sex, age, ethnic origin)
4. Relevant medical history will be obtained and recorded (diabetes mellitus); the smoking status will be recorded and the cardiovascular risk (Framingham Score, see Appendix 7) will be calculated.
5. Transplantation information will be recorded (donor and recipient information).
6. A complete physical examination will be performed.
7. Vital signs will be measured.
8. ECG will be performed.
9. Venous blood will be drawn for measurement of hematology and biochemistry (extended program)
10. Venous blood will be drawn for measurement of viral serology
11. Concomitant medications / significant non-drug therapies will be recorded.
12. Optionally –if part of center standard procedure- a control biopsy may be taken after transplantation. Control biopsies should be assessed by a central nephropathologist. In order to avoid any bias, which could interfere with the objectives of the study, investigators should be kept blinded until completion of the study.

**Visit 2 (week 2) – Control Assessments post Transplantation**

1. Vital signs will be measured.
2. Venous blood will be drawn for measurement of hematology and biochemistry (basic program)
3. Venous blood will be drawn for measurement of the cyclosporine C0-h level.
4. Urinalysis will be performed, the Glomerular Filtration Rate (GFR) will be assessd.
5. Changes in Concomitant medications / significant non-drug therapies will be recorded
6. (Changes in) immunosuppressive medication will be recorded
7. Information on rejection episodes, renal biopsies, hospitalization, infections, and other (S)AEs that occurred since the last visit will be recorded.

**Visit 3 (Week 7 post Tx) – Baseline visit 2**

This visit may be performed within a time window of week 7 and week 8.

1. Patient's eligibility for the study according to in- and exclusion criteria will be checked
2. The patient will be randomized to one of the two treatment arms taking stratification into account (occurrence of rejection between BL1 and BL2).
   Study medication for the following treatment weeks will be handed over to the patient; amount and mode of administration will be explained to the patient
3. A complete physical examination will be performed.

4. (Changes in) smoking status will be recorded and the cardiovascular risk (Framingham Score, see Appendix 7) will be calculated.
5. Vital signs will be measured.
6. ECG will be performed.
7. Venous blood will be drawn for measurement of hematology and biochemistry (complete program)
8. An oral glucose tolerance test (OGTT) with a concomitant insulin and/or C-Peptid measurement will be performed.
9. Venous blood will be drawn for measurement of viral serology
10. Venous blood will be drawn for measurement of the C0-h level of cyclosporine
11. Urinalysis will be performed, the Glomerular Filtration Rate (GFR) will be assessed.
12. Changes in Concomitant medications / significant non-drug therapies will be recorded
13. (Changes in) immunosuppressive medication will be recorded
14. Information on rejection episodes, renal biopsies, hospitalization, infections, and other (S)AEs that occurred since the last visit will be recorded.
15. Optionally – if part of center standard procedure – a control biopsy may be taken. Control biopsies should be assessed by a central nephropathologist and should only prompt therapy in cases of severe rejection, infection or toxicity. In order to avoid any bias, which could interfere with the objectives of the study, investigators should be kept blinded until completion of the study.

**Visit 4 (Week 8) – Maintenance Period**

1. Venous blood will be drawn for measurement of Certican® (Everolimus) trough level – local determination
2. Venous blood will be drawn for measurement of cyclosporine C0h-level – local determination
3. Venous blood will be drawn for measurement of hematology and biochemistry (basic program)
4. Urinalysis will be performed, the Glomerular Filtration Rate (GFR) will be assessed.

**Visit 5 (Week 12) – Maintenance Period**

1. Vital signs will be measured.
2. Returned study medication will be counted. Study medication for the interval to the next study visit will be handed over to the patient; amount and mode of administration will be explained to the patient.

3. Venous blood will be drawn for measurement of hematology and biochemistry (basic program)
4. Venous blood will be drawn for measurement of
   - cyclosporine C0h-level (Control group)
   - Certican® (everolimus) trough level - local determination - (Certican® group)
5. Urinalysis will be performed, the Glomerular Filtration Rate (GFR) will be assessed.
6. Changes in concomitant medications / significant non-drug therapies will be recorded
7. Changes in immunosuppressive medication (including study drug dosage) will be recorded
8. Information on rejection episodes, renal biopsies, hospitalization, infections, and other (S)AEs that occurred since the last visit will be recorded.

**Visit 6 (Week 18) – Maintenance Period**

1. Vital signs will be measured.
2. Returned study medication will be counted. Study medication for the interval to the next study visit will be handed over to the patient; amount and mode of administration will be explained to the patient.
3. Venous blood will be drawn for measurement of hematology and biochemistry (extended program)
4. Venous blood will be drawn for measurement of
   - cyclosporine C0h-level (Control group)
   - Certican® (everolimus) trough level -local determination- (Certican® group)
5. Urinalysis will be performed, the Glomerular Filtration Rate (GFR) will be assessed.
6. Changes in concomitant medications / significant non-drug therapies will be recorded.
7. Changes in immunosuppressive medication (including study drug dosage) will be recorded.
8. Information on rejection episodes, renal biopsies, hospitalization, infections, and other (S)AEs that occurred since the last visit will be recorded.

**Visit 7 (Week 24) – End of Treatment**

In case of early discontinuation, Week 24 assessments need to be performed
- as soon as possible after treatment discontinuation

1. A complete physical examination will be performed
2. (Changes in) smoking status will be recorded and the cardiovascular risk (Framingham Score, see Appendix 7) will be calculated.
3. Vital signs will be measured.
4. ECG will be performed.
5. Returned study medication will be counted.
6. Venous blood will be drawn for measurement of hematology and biochemistry (complete program).
7. An oral glucose tolerance test (OGTT) with a concomitant insulin and/or C-Peptid measurement will be performed.
8. Venous blood will be drawn for measurement of viral serology
9. Venous blood will be drawn for measurement of
   - cyclosporine C0h-level (Control group)
   - Certican® (everolimus) trough level -local determination- (Certican® group)
10. Urinalysis will be performed, the Glomerular Filtration Rate (GFR) will be assessed.
11. Changes in concomitant medications / significant non-drug therapies will be recorded.
12. Changes in immunosuppressive medication (including study drug dosage) will be recorded.
13. Information on rejection episodes, renal biopsies, hospitalization, infections, and other (S)AEs that occurred since the last visit will be recorded.
14. Optionally – if part of center standard procedure – a control biopsy may be taken. Control biopsies should be assessed by a central nephropathologist and should only prompt therapy in cases of severe rejection, infection or toxicity. In order to avoid any bias, which could interfere with the objectives of the study, investigators should be kept blinded until completion of the study.
15. End of Study / End of Treatment information will be obtained.

## 7.2 Information to be collected on screening failures

Patients who signed informed consent but in whom no transplantation was performed for any reason (“not transplanted patients”) will be recorded on the Screening Failure Log. No further information will be obtained.

If the patient fails to be randomized into the trial after Week 7 for any reason, the patient’s number and the reason for non-randomization will be entered on the End of Study Page. Information on CRF for Visits 1 to 3 will be completed, as far as applicable and obtained.

## 7.3 Patient demographics/other baseline characteristics

The following demographic data and other baseline characteristics will be recorded:

- **Demography:** date of birth, sex, and ethnic origin
- **Relevant medical history / current medical conditions:**
  Relevant prior diseases and surgeries as well as concomitant diseases will be recorded with date of diagnosis / surgery and information on whether it is an active problem.
  The smoking status (non-smoker, ex-smoker, smoker, number of cigarettes per day) will be recorded (at BL1, BL2, and Month 6).
  For women, the menopausal status will be recorded.

- **Transplantation information:**
  Donor information
  age, sex, race, kind of donation (cadaveric heart beating, cadaveric non-heart beating), hypertension prior to procurement, viral serology on CMV, EBV, HepC, HBsAg, HIV
  Recipient information
  date of transplantation, renal perfusion date and time, duration of cold ischemia time, number of mismatches at HLA locus, percentage of panel reactive antibodies (PRA) , viral serology on CMV, EBV, HepC, HBsAg, end stage disease leading to transplantation.

## 7.4 Treatments

Records of Simulect®, Myfortic®, Certican®, and Sandimmun® Optoral medications used and dosages administered are to be kept during the study. All changes to the medication dosing regimen should be recorded in the Dosage Administration Record CRF, along with the reason for change and dates.

Drug accountability for Certican® medication will be noted by the investigator and/or study personnel at each visit using pill counts. This information should be captured in the source document at each visit.

## 7.5 Efficacy

The following efficacy variables will be obtained and recorded:

- **Serum Creatinine**
  For the analysis of serum creatinine, venous blood will be drawn and analyzed in the center's local laboratory. Besides changes from baseline, the slope of the decline in reciprocal serum creatinine (1 divided by serum creatinine) versus time will be analyzed as an indicator of progressive renal disease (for the creatinine slope, see Gretz 1994; Lacour 1992; Kaplan et al., 2003; Kasiske et al., 2001).

**Glomerular Filtration Rate (GFR)**

The glomerular filtration rate (GFR) is the best clinical estimate of renal function in health and disease, and correlates well with the clinical severity of renal function disturbances. Several studies have shown that in patients with progressive renal disease, GFR declines or reciprocal serum creatinine levels elevates linearly over time in a predictable manner.

With the help of the serum creatinine values, the GFR will be calculated via **Cockcroft-Gault formula**

For men: $\text{GFR} = \frac{(140 - Age) \times Body\ Weight\ [kg]}{72 \times Serum\ Creatinine\ [mg/dl]}$

For women: $\text{GFR} = \frac{0{,}85 \times (140 - Age) \times Body\ Weight\ [kg]}{72 \times Serum\ Creatinine\ [mg/dl]}$

This equation has been validated in renal transplant patients against the true GFR measured by a radionuclide method and has been confirmed as a very accurate method to calculate the GFR in this specific population (Gaspari et al., 2004).

The GFR calculated according to Cockcroft-Gault method (Cockcroft and Gault, 1976) will be used as the primary outcome measure in this study. As secondary efficacy criterion, the Glomerular Filtration Rate will be calculated in addition using the Nankivell method (Nankivell et al., 1995) and the MDRD method (MDRD = Modification of Diet in Renal Disease Study Group, Levey et al., 1999, Rodrigo et al., 2003; Pierrat et al., 2003).

Calculated value of GFR in mL/min per $1.73m^2$ - **Nankivell formula**

GFR = *6.7 / serum creatinine* + *body weight / 4* – *serum urea / 2-100 / (height)²* + *C*

where Scr is the serum creatinine concentration expressed in mmol/L, BW the body weight in kg, Surea the serum urea in mmol/L, height in m, and the constant C is 35 for male and 25 for female patients.

Calculated value of GFR in ml/min - **MDRD formula**

For men: GFR = 170 x (serum creatinine $^{-0,999}$) x (age$^{-0,176}$) x (urea nitrogen $^{-0,17}$) x (albumin$^{0,318}$)

For women: GFR = 170 x (serum creatinine $^{-0,999}$) x (age$^{-0,176}$) x (urea nitrogen $^{-0,17}$) x (albumin$^{0,318}$) x 0.762

with urea nitrogen = urea / 2.144.

- **Rejection episodes and graft loss**
  All suspected rejection episodes must be recorded on the Rejection CRF, with the date rejection was first suspected, whether a biopsy was performed, whether follow-up biopsies were performed (with subsequent dates), whether anti-rejection therapy was administered, whether the acute rejection was confirmed or with final clinical diagnosis specified, and final clinical outcome.

  Biopsy-proven acute rejection

  In all suspected rejection episodes, a graft core biopsy will be performed prior to or at the latest within 24 hours after the initiation of anti-rejection therapy. Biopsies will be read and interpreted by local pathologists. The results including date of occurrence of first symptoms will be recorded in the Biopsy CRF and will be used for the efficacy analysis. A biopsy-proven acute rejection will be defined as a biopsy graded IA, IB, IIA, IIB, or III (see Appendix 6 for Banff 07 classification).

  Rejection episodes will be reported on the Rejection CRF only, and not on the AE CRF. A Rejection CRF will be completed each time a suspected or biopsy-proven acute rejection has occurred. Rejection episodes occurring more than 10 days after the start of the preceding one will be considered as a new rejection episode.

  Chronic rejection

  Chronic rejection is characterized by a slow progressive decline in renal function and is typically preceded by the histological picture of chronic allograft nephropathy. The presence of biopsy confirmed Grade I, II or III chronic allograft nephropathy by Banff 07 criteria will be assessed on all optional biopsies obtained for clinical suspicion of chronic rejection and reported on the Biopsy CRF.

  Graft loss

  The allograft will be presumed to be lost on the day the patient starts dialysis and is not able to subsequently be removed from dialysis. If the patient undergoes a graft nephrectomy, then the day of nephrectomy is the day of graft loss. This will be reported on the Premature Discontinuation of Study Medication CRF if medication is discontinued prematurely. The reason for graft loss will be recorded on the Graft Loss CRF. If graft loss occurs after the patient has discontinued study medication, the appropriate Follow-Up CRF should be completed. Graft loss is considered a Serious Adverse Event and should be reported on the Serious Adverse Event Case Report Form, which should be faxed to Novartis.

## 7.6 Safety

Safety assessments will consist of monitoring and recording all infections, tumors, AEs and SAEs, the regular monitoring of hematology, blood chemistry, and regular measurement of vital signs.

### Infections

All infection episodes must be recorded on the Infection CRF; they should not be collected on the AE CRF. Infections should be listed with severity, relationships,

sample site, genus and species of microorganism specified, start and end dates, action taken.

CMV infection is defined as seroconversion of a negative patient or isolation of CMV from urine, saliva, blood or any other tissue. Symptomatic or clinically apparent CMV infection should be classified according to the criteria of the Paris CMV workshop [13] into presumptive and proven CMV disease. Briefly, the diagnosis of presumptive CMV disease requires the demonstration of CMV viremia by any method in addition to fever >38°C for at least 2 days in the absence of another clinical source, combined with one of the following findings: atypical lymphocytosis >3%, WBC count <4,000/mm$^3$ or platelet count <100,000/mm$^3$. The diagnosis of proven CMV disease requires, apart from the presence of signs and/or symptoms of organ involvement, the detection of CMV in the affected organ. Particular attention will be given to clinically apparent CMV infections (presumptive and proven CMV disease).

**Tumor Incidence**

The occurrence of any tumor will be recorded on the AE-CRF. Additionally they will be subject to expedited reporting according to the SAE-criteria described below.

**Adverse events**

An adverse event is the appearance or worsening of any undesirable sign, symptom, or medical condition occurring after obtaining informed consent even if the event is not considered to be related to study drug. Study drug includes the investigational drug under evaluation and the comparator drug or placebo that is given during any phase of the trial. Medical conditions/diseases present before starting study drug are only considered adverse events if they worsen after starting study drug. Abnormal laboratory values or test results constitute adverse events only if they induce clinical signs or symptoms, are considered clinically significant, or require therapy.

The occurrence of adverse events should be sought by non-directive questioning of the patient at each visit during the study. Adverse events also may be detected when they are volunteered by the patient during or between visits or through physical examination, laboratory test, or other assessments. As far as possible, each adverse event should be evaluated to determine:

1. the severity grade (mild, moderate, severe)
2. its relationship to the study drug (Certican®, Simulect® dose 3 & 4) (suspected/not suspected)
3. its duration (start and end dates or if continuing at final exam)
4. action taken i.e:
   (a) no action taken;
   (b) study drug dosage adjusted/temporarily interrupted;
   (c) study drug permanently discontinued due to this adverse event;
   (d) concomitant medication taken/adjusted/stopped
   (e) non-drug therapy given;
   (f) hospitalization/prolonged hospitalization)

5. whether it is serious, where a serious adverse event (SAE) is defined as one which:
   - is fatal or life-threatening
   - results in persistent or significant disability/incapacity
   - constitutes a congenital anomaly/birth defect
   - requires inpatient hospitalization or prolongation of existing hospitalization, unless hospitalization is for:
     - routine treatment or monitoring of the studied indication, not associated with any deterioration in condition
     - elective or pre-planned treatment for a pre-existing condition that is unrelated to the indication under study and has not worsened since the start of study drug
     - treatment on an emergency outpatient basis for an event not fulfilling any of the definitions of a SAE given above and not resulting in hospital admission
     - social reasons and respite care in the absence of any deterioration in the patient's general condition
   - is medically significant, i.e., defined as an event that jeopardizes the patient or may require medical or surgical intervention to prevent one of the outcomes listed above
   - is a graft loss
   - is the occurrence of any neoplasm or tumor

**Unlike routine safety assessments, SAEs are monitored continuously and have special reporting requirements; see Section 8.1.**

All adverse events should be treated appropriately. Such treatment may include changes in study drug treatment including possible interruption or discontinuation, starting or stopping concomitant treatments, changes in the frequency or nature of assessments, hospitalization, or any other medically required intervention. Once an adverse event is detected, it should be followed until its resolution, and assessment should be made at each visit (or more frequently, if necessary) of any changes in severity, the suspected relationship to the study drug, the interventions required to treat it, and the outcome.

Information about common side effects already known about the investigational drug can be found in the Investigator Brochure or will be communicated between IB updates in the form of Investigator Notifications. This information will be included in the patient informed consent and should be discussed with the patient during the study as needed.

**<u>Physical examination</u>**

Information about the physical examination must be present in the source documentation at the study site. Significant findings that are present prior to study

enrollment must be included in the Relevant Medical History/Current Medical Conditions CRF. Significant findings made after the start of the study which meet the definition of an AE must be recorded on the AE CRF.

**Vital signs**

Height will be recorded at Baseline visit 1 (Visit 1) only. Vital signs will be recorded at every study visit, and include oral body temperature, radial pulse rate, systolic and diastolic blood pressure and weight (kg). Blood pressure and pulse rate will be assessed after the patient has rested in the sitting position for at least five minutes; blood pressure should be assessed at the same arm each time of determination.

**Electrocardiograms**

Standard ECGs will be performed at visits indicated in Table 7-1. ECGs will be read locally without delay for patients' management.

**Laboratory evaluations**

The center's local laboratory will be used to analyze the clinical laboratory data during the study.

The following variables will be examined:

| | |
|---|---|
| Hematology: | Hemoglobin, leukocytes (differential blood count), absolute neutrophil count and platelet count, hematokrit (HK) |
| Blood chemistry: | creatinine, urea, sodium, potassium, calcium, ALAT, ASAT, protein, AP, albumin, C-reactive protein (CRP), phosphate, magnesium, CPK<br>Additionally at Baseline Visit 1 (= extended program):<br>total cholesterol, LDL, HDL, triglycerides,<br>Additionally at Baseline Visit 2 and Month 6 (= complete program):<br>glucose (fasting), ferritin, transferrin, serum iron<br>total cholesterol, LDL, HDL, triglycerides, |
| Viral Serology: | BK virus, Hepatitis B (HBsAg), Hepatitis C (RNA), HIV (only at Baseline visit 1), CMV |
| Urinalysis: | creatinine, protein, albumin |

For determination of the drug levels see Section 7.9.

Viral serology

All patients will be tested for Hepatitis B (HBsAg), Hepatitis C (RNA), CMV and HIV at Baseline visit 1, earlier tests ≤ 6 months are acceptable. Any patients with a

positive Hepatitis B surface antigen or Hepatitis C or HIV will be excluded. If results are not available at Baseline visit 1, the patient may be included. If results of a newly initiated assessment at Baseline visit 1 or thereafter will be positive, the patient will subsequently be dropped from the study and administered the standard care provided by the center.

Any patients with a negative CMV status should be tested at Baseline visit 1 and Baseline visit 2 for CMV. Furthermore BK virus will be assessed in serum at Baseline visit 2 and at the end of the study.

**New-Onset of Diabetes Mellitus**

At Baseline visit 2 and on week 24 (EOS) assessments will be performed to investigate the development of a diabetes mellitus. Therefore, fasting blood sugar level will be analyzed as well as an oral glucose tolerance test with a concomitant insulin and/or C-Peptid measurement will be performed.

**Cardiovascular Risk (Framingham Score)**

At BL1 (Screening), BL2 (Week 7) and at the End of Study (Month 6) the patients' cardiovascular risk will be calculated using the Framingham score.

This update of the 1991 Framingham coronary prediction algorithm as used in this study (Wilson et al., 1998) provides estimates of total risk of developing one of the following coronary heart diseases: angina pectoris, myocardial infarction, or coronary disease death, over the course of 10 years. The factors used to estimate risk include age, LDL cholesterol, HDL cholesterol, blood pressure, cigarette smoking, and diabetes mellitus. Different score sheets are used for men and women ( Appendix 7).

## 7.7 Tolerability

No specific tolerability measure is used in this study. However, tolerability of study medication can be assessed from the rate of patients who withdraw prematurely from study medication or in whom study medication had to be converted to another immunosuppressive regimen due to abnormal laboratory results, adverse events, or toxicity.

All changes to the immunosuppressive medication dosing regimen will be recorded on the Dosage Administration Record CRF, along with the reason for change.

## 7.8 Health-related Quality of Life

None.

## 7.9 Pharmacokinetics

The actual sample collection date and values (Certican® or Sandimmun® Optoral) will be recorded on CRF.

Cyclosporine whole blood levels will be measured starting at Visit 2. For patients of the Control group cyclosporine blood level will be measured at every study visit, for patients of the CNI-free Certican group cyclosporine blood level will be measured until Sandimmun® Optoral is removed from the immunosuppressive regimen (Visit 3, Week 7).

Certican® (everolimus) blood levels will be measured in patients of the Certican® group starting 7 days after first Certican® dose application, and at every study visit thereafter.

- **Cyclosporine (CsA) whole blood tests**
  Analysis of the cyclosporine whole blood levels will be performed locally at the center. Whole blood level for CsA will be assessed by utilizing a monoclonal immunoassay or HPLC-method on blood taken 5 minutes prior to medication intake (C0-h level), as indicated in the visit schedule (C-0h).
- **Certican® (everolimus) whole blood tests**
  For the analysis of the everolimus whole blood levels 2 ml of blood should be drawn in EDTA tubes. Analysis will be performed using ELISA method. The C-0h level will be obtained with blood sampling within 5 minutes prior to medication intake.

## 7.10 Pharmacogenetics/pharmacogenomics

None.

## 7.11 Other biomarkers

Assessment of basiliximab concentration as well as lymphocyte immunophenotyping may be performed by some of the sites.

For the analysis of basiliximab serum concentration 1 ml venous blood samples should be collected before randomization and on week 12 prior to basiliximab injection. Analysis will be performed using ELISA method. An average serum basiliximab concentration of 0.2 µg/ml is referred to as sufficient to saturate CD25 on peripheral blood lymphocytes (Hoecker et al., 2008; Kovarik et al., 1999)

Analysis of 0.5 ml venous blood will be performed for lymphocyte immunophenotyping. Therefore monoclonal antibodies against two IL-2Rα regions - epitope A which is basiliximab blocked and epitope B which is not bound by basiliximab – will be used to investigate the course of $CD25^+$ T lymphocyte fraction after basiliximab injection (Hoecker et al., 2008).

All samples will be frozen and will be measured in a center laboratory.

### 7.12 Additional Assessments

Further assessments that might be of interest (e.g., Quality of Life, biopsies, etc.) may be performed by some of the sites. The assessment of further parameters may be fixed in additional site specific protocols. Except for the indicated time points (null biopsy and biopsies at Week 7 and 24) no further protocol biopsies are recommended.

The additional protocols will be submitted for review and approval to the responsible ethics committee prior to implementation.

## 8 Safety monitoring

### 8.1 Serious adverse event reporting

To ensure patient safety, every SAE, regardless of suspected causality, occurring after the patient has provided informed consent and until 30 days after the patient has stopped study participation (defined as time of last dose of study drug taken) must be reported to Novartis within 24 hours of learning of its occurrence. Any SAEs experienced after this 4-week period should only be reported to Novartis if the investigator suspects a causal relationship to the study drug. Recurrent episodes, complications, or progression of the initial SAE must be reported as follow-up to the original episode within 24 hours of the investigator receiving the follow-up information. An SAE occurring at a different time interval or otherwise considered completely unrelated to a previously reported one should be reported separately as a new event.

Information about all SAEs is collected and recorded on the Serious Adverse Event Report Form. The investigator must assess the relationship to study drug, complete the SAE Report Form in English, and send the completed, signed form by fax within 24 hours to the local Novartis Clinical Safety & Epidemiology Department. The telephone and telefax number of the contact persons in the local department of Clinical Safety and Epidemiology, specific to the site, are listed in the investigator folder provided to each site. The original copy of the SAE Report Form and the fax confirmation sheet must be kept with the case report form documentation at the study site.

Follow-up information is sent to the same person to whom the original SAE Report Form was sent, using a new SAE Report Form stating that this is a follow-up to the previously reported SAE and giving the date of the original report. Each re-occurrence, complication, or progression of the original event should be reported as a follow-up to that event regardless of when it occurs. The follow-up information should describe whether the event has resolved or continues, if and how it was treated and whether the patient continued or withdrew from study participation.

If the SAE is not previously documented in the Investigator's Brochure or Package Insert (new occurrence) and is thought to be related to the Novartis study drug, a Clinical Safety & Epidemiology Department associate may urgently require further

information from the investigator for Health Authority reporting. Novartis may need to issue an Investigator Notification (IN), to inform all investigators involved in any study with the same drug that this SAE has been reported.

### 8.2 Pregnancies

To ensure patient safety, each pregnancy in a patient on study drug must be reported to Novartis within 24 hours of learning of its occurrence. The pregnancy should be followed up to determine outcome, including spontaneous or voluntary termination, details of the birth, and the presence or absence of any birth defects, congenital abnormalities, or maternal and/or newborn complications.

Pregnancy should be recorded on a Clinical Trial Pregnancy Form and reported by the investigator to the local Novartis Clinical Safety & Epidemiology Department. Pregnancy follow-up should be recorded on the same form and should include an assessment of the possible relationship to the Novartis study drug of any pregnancy outcome. Any SAE experienced during pregnancy must be reported on the SAE Report Form.

### 8.3 Data Safety Monitoring Board

An external and independent Data Safety Monitoring Board (DSMB) will be instituted before study start. The DSMB will review safety-related issues and will be entitled to make recommendations for changes in study conduct. Details on the function of the DSMB will be laid out in a separate DSMB Charter.

## 9 Data review and database management

### 9.1 Site monitoring

Before study initiation, at a site initiation visit or at an investigator’s meeting, a Novartis representative will review the protocol and CRFs with the investigators and their staff. During the study, the field monitor will visit the site regularly to check the completeness of patient records, the accuracy of entries on the CRFs, the adherence to the protocol and to Good Clinical Practice, the progress of enrollment, and to ensure that study drug is being stored, dispensed, and accounted for according to specifications. Key study personnel must be available to assist the field monitor during these visits.

The investigator must maintain source documents for each patient in the study, consisting of case and visit notes (hospital or clinic medical records) containing demographic and medical information, laboratory data, electrocardiograms, and the results of any other tests or assessments. All information on CRFs must be traceable to these source documents in the patient's file. Data not requiring a separate written record will be defined before study start and will be recorded directly on the CRFs, which will be documented as being the source data. The investigator must also keep the original informed consent form signed by the patient (a signed copy is given to the patient).

The investigator must give the monitor access to all relevant source documents to confirm their consistency with the CRF entries. Novartis monitoring standards require full verification for the presence of informed consent, adherence to the inclusion/exclusion criteria, documentation of SAEs, and the recording of data that will be used for all primary and safety variables. Additional checks of the consistency of the source data with the CRFs are performed according to the study-specific monitoring plan. No information in source documents about the identity of the patients will be disclosed.

## 9.2 Data collection

Designated investigator staff must enter the information required by the protocol onto the Novartis CRFs that are printed on 3-part, non-carbon-required paper. Field monitors will review the CRFs for completeness and accuracy and instruct site personnel to make any required corrections or additions. The CRFs are forwarded to Data Management by field monitors, one copy being retained at the investigational site. Once the CRFs are received by Data Management, their receipt is recorded and they are reviewed prior to data entry.

## 9.3 Database management and quality control

Data from the CRFs are entered into the study database by Contract Research Organization staff following their own internal standard operating procedures that have been reviewed and approved by Novartis.

Subsequently, the entered data are systematically checked by Data Management staff, using error messages printed from validation programs and database listings. Obvious errors are corrected by Data Management personnel. Other errors or omissions are entered on Data Query Forms, which are returned to the investigational site for resolution. The signed original and resolved Data Query Forms are kept with the CRFs at the investigator site, and a copy is sent to Novartis so the resolutions can be entered into the database. Quality control audits of all key safety and efficacy data in the database are made prior to locking the database.

Concomitant medications entered into the database will be coded using the WHO Drug Reference List, which employs the Anatomical Therapeutic Chemical classification system. Medical history/current medical conditions and adverse events will be coded using the Medical dictionary for regulatory activities (MedDRA) terminology.

At the conclusion of the study the database has been declared to be complete and accurate and will be locked. Any changes to the database after that time can only be made by joint agreement between the Trial Statistician and Statistical Reporting and the Clinical Trial Leader.

# 10 Statistical methods

The data will be analyzed by Novartis and/or by the designated CRO. Any data analysis carried out independently by the investigator(s) should be submitted to

Novartis before publication or presentation. The final analysis will be done when all patients have completed the study treatment at Visit 6 (Week 24/Month 6) or discontinued prematurely. It is planned that the data from all centers that participate in this protocol will be used, so that an adequate number of patients will be available for analysis.

Data will be summarized with respect to demographic and baseline characteristics, efficacy observations and measurements, safety observations and measurements, and pharmacokinetic measurements. All summary statistics will be presented by treatment group. Categorical variables will be summarized by absolute and relative frequencies. Continuous variables will be summarized by descriptive statistics of mean, standard deviation, minimum, median and maximum. Time-to-event data including rates of affected patients will be assessed by Kaplan-Meier statistics.

The assessment of safety will be based mainly on the frequency of adverse events and on the number of laboratory values that fall outside of pre-determined ranges. Other safety data (e.g. electrocardiogram, vital signs, special tests) will be considered as appropriate. Adverse events will be summarized by presenting, for each treatment group, the number and percentage of patients having any adverse event, having an adverse event in each body system and having each individual adverse event. Any other information collected (e.g. severity or relatedness to study medication) will be listed as appropriate.

## 10.1 Analysis set

### Randomized set

The randomized set will consist of all patients who were randomized at Baseline Visit 2 (Visit 3/Week 7). Patients will be considered randomized when they have received a randomization number, and thus have been assigned to a treatment group. This does not necessarily mean that they have taken, or in fact have been administered, randomized medication.

### Full analysis set

The full analysis set (FAS) will consist of all patients randomized at Baseline Visit 2 (Week 7). Patients will be analyzed according to treatment assigned and their actual stratum. This set may include patients without any data after randomization (methods to account for missing data in the analysis are described in Section 10.4.3 below).

### Per-protocol set

The per-protocol set will consist of a subset of patients of the FAS who did not show major deviations from the protocol procedures that may have an impact on the study outcome. Reasons for exclusion of the per-protocol set may be (but are not limited to): deviations from entry criteria, errors in treatment assignment, use of excluded/forbidden/un-allowed medication, premature discontinuation of randomized treatment or study, poor compliance, loss to follow-up, missing data in the primary

efficacy variable. All criteria that are assumed to have such an impact will be defined before database lock during the central blinded review. The per-protocol set will be used for sensitivity analyses of the primary variable.

**Safety set**

The safety set will consist of all patients in whom transplantation was performed and who received at least one dose of any immunosuppressive medication after Baseline 1. Note: The statement that a patient had no adverse events constitutes a safety assessment.

**Enrolled set**

The enrolled set will consist of all patients who gave informed consent. The enrolled set will be used for analysis of background and demographic characteristics to account for patients who were enrolled but failed to be randomized (and thus are not part of the FAS).

## 10.2 Background and demographic characteristics

Demographic and background information will be summarized for the full analysis (FAS) and the enrolled set. Background information includes prior medication, past/current medical conditions and transplant history.

Medical history will be coded using MedDRA and will be presented by system organ class, MedDRA preferred term and treatment group. Separate tables will be provided for past medical condition and current medical condition. Prior medication will be coded according to WHO Drug Reference List.

## 10.3 Study treatments

### 10.3.1 Study medication

Duration (days) of exposure to each individual component of the immunosuppressive treatment regimen will be summarized. Dosage averages will be calculated including and excluding zero doses for periods of temporary interruption of treatment regardless of whether this was due to safety reasons or patients' non-compliance. Daily dose levels will be summarized with mean, standard deviation, minimum, median, and maximum by time interval. Frequencies of the number of patients with any dose reduction (including temporary dose interruption) as well as the number of dose reductions by reason will be given. These analyses will be performed for the safety and the full analysis sets.

### 10.3.2 Concomitant therapy

Prior and concomitant medications and non-drug therapies will be coded according to WHO-DRL. Medications will be summarized by preferred term, ATC class and treatment group separately for medications either ending before start of study drug

(prior medications) or concomitantly taken through the study (concomitant medications) for the safety set.

## 10.4 Analysis of the primary objective(s)

The primary objective of this study is to demonstrate superior renal function under the Certican®-based regimen as compared to the control regimen.

### 10.4.1 Variable

The primary variable for assessment of renal function is the glomerular filtration rate (GFR) at Month 6, as assessed by the Cockcroft-Gault method (recalculated values, see Section 7.5).

### 10.4.2 Statistical hypothesis, model, and method of analysis

The null hypothesis of no difference in GFR at Month 6 between both treatment groups

$H_0$: $\mu_{Certican} = \mu_{Control}$

will be tested against the two-sided alternative hypothesis of different mean GFR at Month 6 of the Certican® compared to control regimen

$H_1$: $\mu_{Certican} \neq \mu_{Control}$

μ indicates the mean GFR at Month 6.

The hypotheses will be tested with an analysis of covariance (ANCOVA) with treatment, center, and occurrence of rejections before randomization as factors, and GFR at Visit 3 (Baseline 2) as covariate. Raw as well as adjusted means (= LS-means, LS: least square means) will be presented for the treatment contrast together with its confidence interval and a two-sided p-value. The significance level will be 5% (two-sided). The full analysis set (FAS) will be used for the primary analysis. There are no multiplicity issues to be addressed since only the hypothesis of superior efficacy of the Certican® vs. the control regimen will be tested confirmatorily.

### 10.4.3 Handling of missing values/censoring/discontinuations with respect to the primary efficacy variable

If a patient discontinues from the study prematurely after Visit 3/Baseline 2, missing data with respect to the primary efficacy parameter will be imputed via multiple imputation. The multiple imputation procedure will be presented in detail (e.g. seed value, method for imputation) in the statistical analysis plan to be finalized before database lock.

Multiple imputation inference involves three distinct phases:

- The missing data are filled in m times to generate *m* complete data sets.
- The *m* complete data sets are analyzed according to the ANCOVA model.
- The results from the *m* complete data sets are combined for the inference.

For sensitivity, missing data will not be imputed but instead, a mixed model approach for repeated measures (MMRM) will be applied (see Section 10.4.4 below).

### 10.4.4 Supportive analyses

For sensitivity, the following analyses will be performed:

1. The primary analysis will be performed with the PP set. Missing values will be dealt by multiple imputation.
2. The primary analysis will be once more performed for the FAS after having imputed missing values by last observation carried forward (LOCF). If a patient discontinues from the study prematurely after Visit 3/Baseline 2, missing data with respect to the primary efficacy parameter will be substituted by the last recorded value (last observation carried forward, LOCF). The last observation will only be carried forward if a patient has had at least one assessment after BL2, i.e. on Visits 4 to 6. Under the assumption that the GFR will remain fairly stable in the control group but will probably improve in the Certican group, this strategy corresponds to a conservative estimate of the therapy effect within both treatment groups.
3. For sensitivity, the primary efficacy variable will be analyzed using a mixed model for repeated measures (MMRM) approach to deal with missing values occuring after Visit 3 (Week 7/Baseline 2). This analysis will be performed for the FAS.

The course of the GFR will be summarized by visit and treatment group. Changes from BL2 to each post-baseline measure will be calculated by treatment group for each study visit. If patients of the FAS are switched from the control to the Certican group during the study, they will be analyzed in their randomized group. If appropriate, a subgroup analysis will be performed comparing patients of the control with and without switch to Certican treatment including a further analysis comparing the subgroups to the randomized Certican group. If treatment will be modified in the Certican group, they will be analyzed as randomized.

Additionally to the analyses of data from the interval between Week 7 (BL2) and Month 6, the GFR according to Cockcroft-Gault will also be evaluated for the interval between BL1 and Month 6 analogous to the primary analysis for the FAS. No further sensitivity analyses will be performed for this time interval.

Results from supportive analyses will be interpreted in an explorative manner.

## 10.5 Analysis of secondary objectives

### 10.5.1 Efficacy (secondary)

The following secondary efficacy variables will be analyzed in an explorative manner for the full analysis set (FAS):

- Renal function as assessed by Nankivell and MDRD at Month 6 after Tx
- Serum creatinine at Month 6 and creatinine slope (1/serum creatinine versus time) between baseline and Month 6

- Occurrence of biopsy proven acute rejection, graft loss, or death at Month 6 after Tx
- Occurrence of treatment failure defined as composite endpoint of biopsy proven acute rejection, graft loss, death, loss to follow up, discontinuation due to lack of efficacy or due to adverse event, or conversion to another regimen up to or at Month 6 after Tx.

Additionally to the analyses of data from the interval between BL2 (Week 7) and Month 6, all secondary outcome criteria will also be evaluated exploratorily for the interval between BL1 and Month 6.

**Renal function:**

Glomerular filtration rate (GFR) will be computed according to Nankivell and MDRD method, respectively, and assessed at Month 6 post Tx by an ANCOVA model analogous to the primary analysis. Descriptive summary statistics will be provided by treatment group and visit.

Furthermore, serum creatinine at Month 6 will be analyzed by an ANCOVA model analogous to the primary analysis.

The creatinine slope (1/serum creatinine versus time) will be determined from all assessments between baseline and Month 6 using a linear regression model.

**Event data:**

Event rates will be estimated using the Kaplan-Meier method to handle adequately patients who discontinue the treatment prior to suffering from an event. Kaplan-Meier curves will be displayed graphically. The two groups will be compared using the logrank test. The following events or combination of events will be considered:

- Biopsy proven acute rejection (BPAR), defined as a rejection which was acute and proven by biopsy.
- Graft loss (GL)
- Death
- Treatment failure (composite endpoint) is defined as BPAR or GL or death or loss to follow-up or discontinuation due to lack of efficacy or adverse event or conversion to another regimen.

### 10.5.2 Safety

Safety variables will be analyzed separately for the safety population a) for the period between Week 7 (Baseline 2) and Month 6 (or date of premature discontinuation of the study), b) for the period between Baseline 1 and Baseline 2, and c) for the total treatment period (Baseline 1 (prior to Tx) to Month 6).

The safety variables will be as follows:

- Incidence and severity of Adverse Events (AEs) and Serious Adverse Events (SAEs)
- Incidence of infections

- Incidence of tumors
- Changes in cardiovascular risk (Framingham Score, ECG)
- Occurrence of proteinuria
- Incidence of new onset of diabetes mellitus (NODM)

### AEs/Infections/Tumors

Generally, infections and tumor data are analyzed together with AE data. In addition, infection and tumor data will be analyzed and presented separately.

Data collected by AE CRFs and by Infection CRFs are to be coded with the MedDRA dictionary that gives preferred term and body system information. The incidence of AE will be summarized by body system, preferred term, severity, and relationship to study drug. All information pertaining to AE noted during the study will be listed by treatment group and patient, detailing verbatim given by the investigator, the preferred term, the body system, start/end dates, severity, seriousness, relationship to study drug and action taken. The AE onset will also be shown relative (in number of days) to the day of initial dose per study period (within the first 6 weeks, after BL2).

In addition to being analyzed similarly as AE, as described above, the relative frequency of infections by type and micro-organism will be tabulated as well as the frequency of tumors by affected organ.

AE occurring 7 or more days after the discontinuation of study medication will not be considered as treatment-emergent and will not be included in AE/infection summary tables.

### Laboratory data

Abnormalities according notable criteria (see Appendix 2) will be identified. The proportions of patients with clinically notable abnormalities according to the notable criteria will be summarized. Shifts from baseline value to worst post-baseline value based on the normal ranges will be presented graphically (Amit et al., 2008). The worst observation is defined as the highest or lowest measure during the different observations periods whereby high or low are chosen according to the direction of abnormality. Further, descriptive statistics of change from baseline of all laboratory variables will be presented by visit. A by-patient listing of all clinically notable abnormal laboratory data will be generated. Only assessments obtained up to 7 days after the discontinuation of study medication will be considered “on-treatment” and analyzed with relationship to immunosuppressive therapy.

The relative frequency of proteinurea will be tabulated by treatment group and visit.

### Vital signs

Vital signs variables include measurements of oral body temperature, systolic and diastolic blood pressures, pulse and body weight. Vital signs will be examined for abnormal values and change from Baseline according to pre-specified clinically

notable criteria (see Appendix 2). Appropriate incidence rates of clinically notable abnormalities for between-group differences will be provided. Shifts from baseline value to worst post-baseline value based on the normal ranges/notable criteria will be presented graphically (Amit et al., 2008). The worst observation is defined as the highest or lowest measure during the different observations periods whereby high or low are chosen according to the direction of abnormality. Further, descriptive statistics of change from baseline of all vital signs variables will be presented by visit. A by-patient listing of all clinically notable abnormal vital signs will be generated. Only assessments obtained up to 7 days after the discontinuation of study medication will be considered “on-treatment”.

**Electrocardiograms**

At BL1, BL2 and at Month 6 standard ECGs will be performed and any clinically significant abnormalitys will be recorded.

**Cardiovascular risk (Framingham Score)**

At BL1, BL2 and at Month 6 the cardiovascular risk will be calculated using the Framingham Score (see Appendix 7). Descriptive statistics for changes from BL1 to BL2 and from BL2 to Month 6 will be presented. Changes from baseline will be tested using adequate tests.

### 10.5.3 Tolerability

Tolerability of study medication will be assessed from the rate of patients who withdraw prematurely from study medication or in whom study medication had to be converted to another immunosuppressive regimen due to abnormal laboratory results or adverse events. A number of reasons are pre-defined in Section 7.7 of this protocol. The frequency distribution of these reasons will be presented with absolute and percent values. The most frequent reasons (total $n \geq 5$) for discontinuation and/or conversion as well as the total number of patients who are affected by such intolerability of study medication will be compared between the treatment groups using Fisher’s exact test.

### 10.5.4 Resource utilization

Not applicable.

### 10.5.5 Health-related Quality of Life

Not applicable.

### 10.5.6 Pharmacokinetics

Analysis of the cyclosporine blood levels and Certican® (everolimus) blood levels will be done by treatment group for the treatment phase (up to Month 6).

Blood level values of cyclosporine will be summarized by descriptive statistics by visit and treatment group. The number of patients with deviations from the therapeutic window (see Section 6.5.2) will be counted and tabulated by visit and in total.

Trough levels of everolimus will be summarized descriptively by visit up to Month 6. The number of patients with deviations from the therapeutic window (see Section 6.5.1) will be counted and tabulated separately by visit and in total.

### 10.5.7 Pharmacogenetics/pharmacogenomics

Not applicable.

### 10.5.8 Biomarkers

Biomarker variables (e.g. CD25 saturation) will be analyzed separately and reported in an extension to the Clinical Study Report.

## 10.6. Interim analysis

No interim analysis is planned.

## 10.7 Sample size calculation

From data for the subgroup of elderly patients in study CRAD001A2418, the probable difference in the GFR (Cockcroft-Gault) between the CNI-free regimen group and the standard regimen group is estimated as $\delta = 8$ ml/min with a standard deviation of 16 ml/min at Month 6 after transplantation. Assuming a difference in the mean GFR of 8 ml/min (60 ml/min in the CNI-free group and 52 ml/min in the standard regimen group) and a common standard deviation of 19 ml/min, a two-group t-test with a 5% two-sided significance level will have 80% power to detect this difference when the sample sizes in the CNI-free group is 135 and in the standard regimen group is 68, respectively (a total sample size of 203). Taking into account a common drop-out rate of 10%, 162 patients need to be randomized into the CNI-free group, and 82 into the standard regimen group (a total drop-out adjusted sample size of 244 patients). Enrollment will be continued until the required sample size will be achieved.

# 11 Discussion and rationale for study design features

The study design includes an initial phase of 6 weeks with an Sandimmun® Optoral based immunosuppressive regimen which is required by clinical needs. Dose adjustments after randomization to the treatment regimens in the CNI-free group follows experience from previous clinical trials with everolimus (see Section 1 of this protocol).

Blinding of the two study treatments was not reasonable in this trial because dosing of Sandimmun® Optoral and Certican® has to be based upon trough levels. Therefore the study has to be planned open-label. However, the endpoint of the study is GFR which is an objective outcome variable depending on laboratory measures. An

influence of open-label treatment on this primary outcome variable is limited because dosing behavior will mainly be governed by drug trough levels but only in a few expected cases of overt renal toxicity by the creatinine level.

Because early cyclosporine withdrawal in previous studies was associated with better renal function, but slightly increased frequency of rejection, cyclosporine withdrawal in this study is attempted with the additional application of 2 doses of Basiliximab. It is to be expected that the immunological risk at this point in time is low, and the benefit of cyclosporine elimination will continue. For this purpose, a CNI-free regimen will be compared with a standard Sandimmun® Optoral based immunosuppressive regimen. It is expected, that the CNI-free regimen will result in a superior renal function and that the CNI-free are as safe and effective as the standard Sandimmun® Optoral-based regimen with regard to occurrence of biopsy proven acute rejection episodes, graft loss, death, and lost to follow up, as well of treatment failure (composite endpoint).

A maintenance treatment period of 18 weeks under both regimens is considered sufficiently long to explore any meaningful differences between the groups in the glomerular filtration rate, which is the primary efficacy outcome measure. This has been confirmed in previous clinical trials, where CNI-withdrawal let to an immediate and significant improvement of renal function within a few weeks. The design chosen for this study was also used in other switch studies (Johnson et al., 2001; Flechner et al., 2002).

In summary, the present study design is adequate for investigating the influence of different immunosuppressive regimens with standard or without CNI on short- and long-term renal function.

# 12 References

1. Abramowicz D, Manas D, Lao M, Vanrenterghem Y, Del Castillo D, Wijngaard P, Fung S; Cyclosporine Withdrawal Study Group. Cyclosporine withdrawal from a mycophenolate mofetil-containing immunosuppressive regimen in stable kidney transplant recipients: a randomized, controlled study. Transplantation. 2002;74:1725-1734.

2. Abramowicz D, Del Carmen Rial M, Vitko S, del Castillo D, Manas D, Lao M, Gafner N, Wijngaard P; Cyclosporine Withdrawal Study Group. Cyclosporine withdrawal from a mycophenolate mofetil-containing immunosuppressive regimen: results of a five-year, prospective, randomized study. J Am Soc Nephrol. 2005 Jul;16(7):2234-40.

3. Amado Andres Belmonte. Renal function at month 6 is similar with immediate vs. delayed tacrolimus administration in elderly renal transplant recipients. Senior study presented at ESOT 2007 Congress 30 September- 3 October 2007. Prague. Abstract O092

4. Amit O, Heiberger RM, Lane PW: Graphical approaches to the analysis of safety data from clinical trials. Pharmaceut. Statist. 2008; 7: 20–35

5. Arbogast H, Hueckelheim H, Schneeberger H et al. A calcineurin antagonist-free induction/maintenance strategy for immunosuppression in elderly recipients of renal allografts from elderly cadaver donors: long-term results from a prospective single center trial. Clin Transplant. 2005 Jun; 19(3): 309-315.

6. Arns W, Breuer S, Choudhury S et al. Enteric-coated mycophenolate sodium delivers bioequivalent MPA exposure compared with mycophenolate mofetil. Clin Transplant. 2005 Apr; 19(2): 199-206.

7. Arns W, Petra Glander, Robert Schuhmann, Daniel Ottenberg, I Mai, Wolfgang H Fischer, Hans H Meumayer, Klemens Budde. Conversion from tacrolimus to everolimus does not influence the pharmacokinetic but increases pharmacodynamic response of mycophenolate sodium in renal transplant patients. Transplantation. 2006 Jul 15;82(1 Suppl 2):488.

8. Arns W. et al. Conversion from tacrolimus to everolimus does not influence the pharmacokinetic but increases pharmacodynamic response of mycophenolate sodium in renal transplant patients. Transplantation 82 (1 Suppl 2):488; 2006.

9. Baboolal K. A phase III prospective, randomized study to evaluate concentration-controlled sirolimus (rapamune) with cyclosporine dose minimization or elimination at six months in de novo renal allograft recipients. Transplantation. 2003 Apr 27;75(8):1404-8.

10. Budde K, Bauer S, Hambach P et al. Pharamacokinetic and pharmacodynamic comparison of enteric-coated mycophenolate sodium and mycophenolate mofetil in maintenance renal transplant patients. Am J Transplant. 2007 Apr; 7(4): 888-98.

11. Budde K, Curtis J, Knoll G, Chan L, Neumayer HH, Seifu Y, Hall M. Enteric-coated mycophenolate sodium can be safely administered in maintenance renal transplant patients: results of a 1-year study. Am J Transplant 2004;4(2):237-43.

12. Budde K, Bosmans J-L, Zeier M *et al*. Safety and efficacy of reduced or full dose of cyclosporine (Neoral®) in combination with mycophenolate sodium (Myfortic®), basiliximab (Simulect®), and steroids in *de novo* kidney transplant recipients. International Society of Transplantation XX Congress, 5-10 September 2005, Vienna. Abstract 0212.

13. Budde K,Glander P, Schuhmann R, Ottenberg D, Mai I, Bauer S, Fischer W, Kamar N, Neumayer HH, Arns W. Conversion from cyclosporine to everolimus leads to better renal function and to profound changes in everolimus and mycophenolate metabolism Transplantation 82 (1 Suppl 2):999; 2006.

14. Budde K, Glander P. Pharmacokinetic principles of immunosuppressive drugs. Ann Transplant. 2008;13(3):5-10.

15. Budde K, Arns W, Glander P, Sommerer C, Ariatabar T, Kramer S, Eichele K, Fischer W, Zeier M, Pharmacokinetic and pharmacodynamic comparison of an initially intensified dosing regimen versus a standard dosing regimen of enteric-coated mycophenolate sodium (EC-MPS) in renal transplant patients. ICTS 2008, Sydney

16. Campistol JM, Eris J, Oberbauer R, Friend P, Hutchison B, Morales JM, Claesson K, Stallone G, Russ G, Rostaing L, Kreis H, Burke JT, Brault Y, Scarola JA, Neylan JF. Sirolimus therapy after early cyclosporine withdrawal reduces the risk for cancer in adult renal transplantation. J Am Soc Nephrol. 2006 Feb;17(2):581-9.

17. Certican® (everolimus, SDZ RAD, RAD, RAD001). Transplantation. Oral formulation. Mayer H, Compiler: Investigator's Brochure. Edition 5. Novartis Pharma AG: Basel, Switzerland; 02-Jun-2003.

18. Citterlo F, Scata MC, Violi P, Romagnoli J, Pozzetto U, Nanni G, Castagneto M. Rapid conversion to sirolimus for chronic progressive deterioration of the renal function in kidney allograft recipients. Transplant Proc 2003;35:1292-1294.

19. Cockcroft DW, Gault MH. Prediction of creatinine clearance from serum creatinine. Nephron. 1976; 16(1): 31-41.

20. CPMP (1998): Note for Guidance on Statistical Principles for Clinical Trials. CPMP/ICH/363/96.

21. Diekmann F, Budde K, Oppenheimer F, Fritsche L, Neumayer HH, Campistol JM. Predictors of success in conversion from calcineurin inhibitor to sirolimus in chronic allograft dysfunction. Am J Transplant. 2004 Nov;4(11):1869-75.

22. Diekmann F, Campistol JM. Conversion from calcineurin inhibitors to sirolimus in chronic allograft nephropathy: benefits and risks. Nephrol Dial Transplant. 2006 Mar;21(3):562-8.

23. Dominguez J, Mahalati K, Kiberd B, McAlister VC, MacDonald AS. Conversion to rapamycin immunosuppression in renal transplant recipients: report of an initial experience. Transplantation 2000;70:1244-1247.

24. Doyle SE, Matas AJ, Gillingham K, Rosenberg ME. Predicting clinical outcome in the elderly renal transplant recipient. Kidney Int. 2000 May;57(5):2144-50.

25. Dudley C et al. Transplantation 2002; 2 (Suppl. 3):148 (Abs. 41).

26. Dunn C, Croom KF. Everolimus: a review of its use in renal and cardiac transplantation. Drugs 66:547-70; 2006.

27. Egidi MF, Cowan PA, Naseer A, Gaber AO. Conversion to Sirolimus in solid organ transplantation: a single-center experience. Transplant Proc 2003;35(3 Suppl):131S-137S.

28. Ekberg H, Grinyó J, Nashan B et al., on behalf of the CAESAR study group study. Cyclosporine Sparing with Mycophenolate Mofetil, Daclizumab and Corticosteroids in Renal Allograft Recipients: The CAESAR Study. Am J Transplant. 2007a Mar;7(3):560-70.

29. Ekberg H, Tedesco-Silva H, Demirbas A, Vítko S, Nashan B, Gürkan A, Margreiter R, Hugo C, Grinyó JM, Frei U, Vanrenterghem Y, Daloze P, Halloran PF; ELITE-Symphony Study. Reduced exposure to calcineurin inhibitors in renal transplantation. N Engl J Med. 2007b Dec 20;357(25):2562-75.

30. Emparan C, Wolters H, Laukötter M, Senninger N. Long-term results of calcineurin-free protocols with basiliximab induction in “old-to-old” programs. Transplant Proc. 2004 Nov; 36(9): 2646-8.

31. Eisen HJ, Tuzcu EM, Dorent R, Kobashigawa J, Mancini D, Valantine-von Kaeppler HA, Starling RC, Sorensen K, Hummel M, Lind JM, Abeywickrama KH, Bernhardt P; RAD B253 Study Group. Everolimus for the prevention of allograft rejection and vasculopathy in cardiac-transplant recipients. N Engl J Med. 2003;349:847-858.

32. Fabrizii V, Kovarik J, Bodingbauer M, Kramar R, Hörl WH,, Winkelmayer WC. Long-term patient and graft survival in the Eurotransplant Senior Program: A single-center experience. Transplantation 2005 Sept; 80(5): 582-589.

33. Fellström B. Cyclosporine toxicity. Transpl Proc 2004;36(Suppl 2S):220S-223S.

34. Flechner SM, Goldfarb D, Modlin C, Feng J, Krishnamurthi V, Masstroianni B, Savas K, Cook DJ, Novick AC. Kidney transplantation without calcineurin inhibitor drugs: a prospective, randomized trial of sirolimus versus cyclosporine. Transplantation 2002;74:1070-1076.

35. Flechner SM, Kurian SM, Solez K, Cook DJ, Burke JT, Rollin H, Hammond JA, Whisenant T, Lanigan CM, Head SR, Salomon DR. De novo kidney transplantation without use of calcineurin inhibitors preserves renal structure and function at two years. Am J Transplant. 2004 Nov;4(11):1776-85

36. Formica RN Jr, Lorber KM, Friedman AL, Bia MJ, Lakkis F, Smith JD, Lorber MI. The evolving experience using everolimus in clinical transplantation. Transpl Proc 2004;36(Suppl 2S):495S-499S.

37. Frei U, Noeldeke J, Machold-Fabrizzi V, et al. Prospective age-matching in elderly kidney transplant recipients – a 5-year analysis of the Eurotransplant Senior Program. Am J Transplant 2008 Jan; 8(1): 50-57.

38. Fritsche L, Hörstrup J, Budde K, et al. Old-for-old kidney allocation allows successful expansion of the donor and recipient pool. Am J of Transplant 2003 Nov; 3(11): 1434-1439.

39. Gaspari F, Ferrari S, Stucchi N, et al.; on the behalf of the MY.S.S. study investigators. Performance of different prediction equations for estimating renal function in kidney transplantation. Am J Transplant. 2004 Nov;4(11):1826-1835.

40. Giessing M, Budde K. Sirolimus and lymphocele formation after kidney transplantation: an immunosuppressive medication as co-factor for a surgical problem? Nephrol Dial Transplant. 2003;18:448-449.

41. Goel M, Flechner SM, Zhou L, Mastroianni B, Savas K, Derweesh I, Patel P, Modlin C, Goldfarb D, Novick AC. The influence of various maintenance immunosuppressive drugs on lymphocele formation and treatment after kidney transplantation. J Urol. 2004;171:1788-1792.

42. Gretz NM. How to assess the rate of progression of chronic renal failure in children? Pediatr Nephrol. 1994 Aug; 8(4):499-504.

43. Hariharan S, Kasiske B, Matas A, Cohen A, Harmon W, Rabb H. Surrogate markers for long-term renal allograft survival. Am J Transplant. 2004;4:1179-1183.

44. Höcker B, Kovarik JM, Daniel V, Opelz G, Fehrenbach H, Holder M, Hoppe B, Hoyer P, Jungraithmayr TC, Köpf-Shakib S, Laube GF, Müller-Wiefel DE, Offner G, Plank C, Schröder M, Weber LT, Zimmerhackl LB, Tönshoff B. Pharmacokinetics and immunodynamics of basiliximab in pediatric renal transplant recipients on mycophenolate mofetil comedication. Transplantation. 2008 Nov 15;86(9):1234-40

45. Hong JC, Kahan BD. Use of anti-CD25 monoclonal antibody in combination with rapamycin to eliminate cyclosporine treatment during the induction phase of immunosuppression. Transplantation. 1999 Sep; 68(5): 701-4.

46. Houde I, Isenring P, Boucher D, Noel R, Lachanche JG. Mycophenolate mofetil, an alternative to cyclosporine A for long-term immunosuppression in kidney transplantation? Transplantation. 2000;70:1251-1253.

47. Hricik DE, for the Rapamune Renal Function Study Group. Improved renal function with cyclosporine elimination in sirolimus-treated renal transplant recipients: one-year results from a phase II trial. Am J Transpl. 2001;1(Suppl 1):149S.

48. Jain A, Reyes J, Kashyap R, Rohal S, Abu-Elmagd K, Starzl T, Fung J: What have we learned about primary liver transplantation under tacrolimus immunosuppression? Long-term follow-up of the first 1000 patients. Ann Surg 1999;230:441-449

49. Johnson RWG, Kreis H, Oberbauer R, Brattström C, Claesson K, Eris J. Sirolimus allows early cyclosporine withdrawal in renal transplantation resulting in improved renal function and lower blood pressure. Transplantation Sep 2001;72(5):777-786.

50. Kahan B. Is there a rationale to combine cyclosporin and sirolimus? Nephrol Dial Transplant. 1996 Jan;11(1):21-3.

51. Kahan BD, Rajagopalan PR, Hall M. Reduction of the occurrence of acute cellular rejection among renal allograft recipients treated with basiliximab, a chimeric anti-interleukin-2-receptor monoclonal antibody. United States Simulect Renal Study Group. Transplantation. 1999 Jan 27;67(2):276-84.

52. Kahan B et al. Efficacy of sirolimus compared with azathioprine for reduction of acute renal allograft rejection: a randomised multicentre study. The Rapamune US Study Group. Lancet. 2000 Jul 15;356(9225):194-202.

53. Kahan BD. Sirolimus: a ten-year perspective. Transplant Proc. 2004;36:71-75.

54. Kamar N, Garrigue V, Karras A et al. Impact of early or delayed cyclosporine on delayed graft function in renal transplant recipients: a randomized, multicenter study. Am J Transplant. 2006 May; 6(5): 1042-8.

55. Kaplan B, Meier-Kriesche HU, Minnick P et al. Randomized calcineurin inhibitor cross over study to measure the pharmacokinetics of co-administered enteric-coated mycophenolate sodium. Clin Transplant. 2005 Aug; 19(4): 551-8.

56. Kaplan B, Meier-Kriesche HU, Napoli KL, Kahan BD. The effects of relative timing of sirolimus and cyclosporine microemulsion formulation coadministration on the pharmacokinetics of each agent. Clin Pharmacol Ther 1998; 63: 48-53.

57. Kaplan B, Schold J, Meier-Kriesche HU. Poor predictive value of serum creatinine for renal allograft loss. Am J Transplant. 2003 Dec; 3(12):1560-1565.

58. MA, Hernandez D Kasiske BL. Cardiovascular disease after renal transplantation. Semin Nephrol. 2000 Mar;20(2):176-87.

59. Kasiske BL, Andany, Silkensen J, Rabb H, McClean J, Roel JP, Danielson B. Comparing methods for monitoring serum creatinine to predict late renal allograft failure. Am J Kidney Dis. 2001 Nov; 38(5):1065-1073.

60. Kasiske BL, Snyder JJ, Gilbertson D, Matas AJ. Diabetes mellitus after kidney transplantation in the United States. Am J Transplant. 2003 Feb; 3(2):178-85.

61. Kovarik JM, Rawlings E, Sweny P, Fernando O, Moore R, Griffin PJ, Fauchald P, Albrechtsen D, Sodal G, Nordal K, Amlot PL. Pharmacokinetics and immunodynamics of chimeric IL-2 receptor monoclonal antibody SDZ CHI 621 in renal allograft recipients. Transpl Int. 1996;9 Suppl 1:S32-3.

62. Kovarik JM, Kahan BD, Rajagopalan PR, Bennett W, Mulloy LL, Gerbeau C, Hall ML. Population pharmacokinetics and exposure-response relationships for basiliximab in kidney transplantation. The U.S. Simulect Renal Transplant Study Group. Transplantation. 1999 Nov 15;68(9):1288-94.

63. Kovarik JM, Kahan BD, Kaplan B, Lorber M, Winkler M, Rouilly M, Gerbeau C, Cambon N, Boger R, Rordorf C; Everolimus Phase 2 Study Group. Longitudinal assessment of everolimus in de novo renal transplant recipients over the first post-transplant year: pharmacokinetics, exposure-response relationships, and influence on cyclosporine. Clin Pharmacol Ther 2001 Jan; 69(1):48-56.

64. Kovarik JM, Pescovitz MD, Sollinger HW, Kaplan B, Legendre C, Salmela K, Book BK, Gerbeau C, Girault D, Somberg K; Simulect Phase IV Sudy group. Differential influence of azathioprine and mycophenolate mofetil on the disposition of basiliximab in renal transplant patients. Clin Transplant. 2001 Apr;15(2):123-30.

65. Kovarik JM, Kalberg J, Figueiredo J, Rouilly M, Frazier OL, Rordorf C. Differential influence of two cyclosporine formulations on everolimus pharmacokinetics: a clinically relevant pharmacokinetic interaction. J Clin Pharmacol 2002a;42:95-99.

66. Kovarik JM, Hartmann S, Figueiredo J, Rordorf C, Golor G, Lison A, Budde K, Neumayer HH. Effect of food on everolimus absorption: quantification in healthy subjects and a confirmatory screening in patients with renal transplants. Pharmacotherapy. 2002b;22:154-159.

67. Kovarik JM, Kaplan B, Tedesco Silva H, Kahan BD, Dantal J, Vitko S, Boger R, Rordorf C. Exposure-response relationships for everolimus in de novo kidney transplantation: defining a therapeutic range. Transplantation. 2002c; 73(6): 920-925.

68. Kovarik JM, Curtis JJ, Hricik DE, Pescovitz MD, Scantlebury V, Vasquez A.Differential pharmacokinetic interaction of tacrolimus and cyclosporine on everolimus. Transplant Proc. 2006 Dec;38(10):3456-8.

69. Kreis H, Cisterne JM, Land W, Wramner L, Squifflet JP, Abramowicz D, Campistol JM, Morales JM, Grinyo JM, Mourad G, Berthoux FC, Brattström C, Lebranchu Y, Vialtel P. Sirolimus in association with mycophenolate mofetil induction for the prevention of acute graft rejection in renal allograft recipients. Transplantation. 2000 Apr 15;69(7):1252-60

70. Kreis H, Oberbauer R, Campistol JM, Mathew T, Daloze P, Schena FP, Burke JT, Brault Y, Gioud-Paquet M, Scarola JA, Neylan JF; Rapamune Maintenance Regimen Trial. Long-term benefits with sirolimus-based therapy after early cyclosporine withdrawal. J Am Soc Nephrol. 2004;15:809-817.

71. Kuypers DR Benefit-risk assessment of sirolimus in renal transplantation. Drug Saf. 2005;28(2):153-81.

72. Lacour B. Creatinine and renal function. Nephrologie (France). 1992;13(2):73-81.

73. Levey AS, Bosch JP, Lewis JB, Greene T, Rogers N, Roth D. A more accurate method to estimate glomerular filtration rate from serum creatinine: a new

prediction equation. Modification of Diet in Renal Disease Study Group. Ann Intern Med. 1999 Mar;130(6):461-470.

74. Lo A, Egidi MF, Gaber LW, Shokouh-Amiri MH, Nazakatgoo N, Shisher JS, Gaber AO. Observations regarding the use of sirolimus and tacrolimus in high-risk cadaveric renal transplantation. Clin Transplant 2004;18:53-61.

75. Lorber MI, Mulgaonkar S, Butt KMH, et al. Everolimus versus mycophenolate mofetil in the prevention of rejection in de novo renal trasnplant recipients: a 3-year randomized, multicenter, phase III study. Transplantation 2005;80:244-252.

76. MacDonald AS; RAPAMUNE Global Study Group. A worldwide, phase III, randomized, controlled, safety and efficacy study of a sirolimus/cyclosporine regimen for prevention of acute rejection in recipients of primary mismatched renal allografts. Transplantation. 2001;71:271-280.

77. Marcén R, Morales JM, del Castillo D et al. Posttransplant diabetes mellitus in renal allograft recipients: A prospective multicenter study at 2 years. Transplant Proc. 2006 Dec ; 38(10): 3530-3532.

78. Martins PN, Pratschke J, Pascher A et al. Age and immune response in organ transplantation. Transplantation 2005 Jan; 79(2):127-132.

79. McKane W, Kanganas C, Preston R, Cairns T, Hakim N, Palmer A, Taube D: Treatment of calcineurin inhibitor toxicity by dose reduction plus introduction of mycophenolate mofetil. Transplant Proceedings 2001;33:1224-1225

80. Meier-Kriesche HU, Ojo A, Hanson J, et al. Increased immunosuppressive vulnerability in elderly renal transplant recipients. Transplantation 2000 Mar; 69(5): 885-889.

81. Meier-Kriesche HU, Ojo AO, Cibrik DM et al. Relationship of recipient age and development of chronic allograft failure. Transplantation. 2000 Jul; 70(2): 306-10.

82. Meier-Kriesche HU, Steffen BJ, Chu AH, Loveland JJ, Gordon RD, Morris JA, Kaplan BD. Sirolimus with Neoral Versus Mycophenolate Mofetil with Neoral is Associated with Decreased Renal Allograft Survival Am J Transplant 2004;4(12): 2058-2066.

83. Meier-Kriesche HU, Schold JD, Srinivas TR, Howard RJ, Fujita S, Kaplan B. Sirolimus in combination with tacrolimus is associated with worse renal allograft survival compared to mycophenolate mofetil combined with tacrolimus. Am J Transplant. 2005 Sep;5(9):2273-80.

84. Mehrabi A, Fonouni H, Wente M, Sadeghi M, Eisenbach C, Encke J, Schmied BM, Libicher M, Zeier M, Weitz J, Buchler MW, Schmidt J. Wound complications following kidney and liver transplantation. Clin Transplant. 2006;20 Suppl 17:97-110.

85. Miller LW. Cardiovascular toxicities of immunosuppressive agents. Am J Transplant. 2002 Oct;2(9):807-18.

86. Moien-Afshari F, McManus BM, Laher I. Immunosuppression and transplant vascular disease: benefits and adverse effects. Pharmacol Ther. 2003;100:141-156.

87. Mourad G, Garrigue V, Squifflet JP et al. Induction versus noninduction in renal transplant recipients with a tacrolimus-based immunosuppression. Transplantation. 2001 Sep; 72(6): 1050-5.

88. Mulay AV, Hussain N, Fergusson D, Knoll GA. Calcineurin inhibitor withdrawal from sirolimus-based therapy in kidney transplantation: a systematic review of randomized trials. Am J Transplant. 2005 Jul;5(7):1748-56.

89. Nankivell BJ, Gruenewald SM, Allen RD, Chapman JR. Predicting glomerular filtration rate after kidney transplantation. Transplantation. 1995 Jun 27;59(12):1683-9.

90. Nankivell BJ, Fenton-Lee CA, Kuypers DR, Cheung E, Allen RD, O'Connell PJ, Chapman JR. Effect of histological damage on long-term kidney transplant outcome. Transplantation 2001;71:515-523

91. Nankivell BJ, Borrows RJ, Fung CL, O'Connell PJ, Allen RD, Chapman JR. The natural history of chronic allograft nephropathy. N Engl J Med. 2003;349:2326-2333.

92. Nashan B, Moore R, Amlot P, Schmidt AG, Abeywickrama K, Soulillou JP. Randomised trial of basiliximab versus placebo for control of acute cellular rejection in renal allograft recipients. CHIB 201 International Study Group. Lancet. 1997 Oct 25;350(9086):1193-8

93. Nashan B, Curtis J, Ponticelli C, et al for the 156 Study Group. Everolimus and reduced-exposure cyclosporine in de novo renal-transplant recipients: a three-year phase II, randomized, multicenter, open-label study. Transplantation 78:1332-1340; 2004.

94. Neumayer HH, Paradis K, Korn A, Jean C, Fritsche L, Budde K, Winkler M, Kliem V, Pichlmayr R, Hauser IA, Burkhardt K, Lison AE, Barndt I, Appel-Dingemanse S. Entry-into-human study with the novel immunosuppressant SDZ RAD in stable renal transplant recipients. Br J Clin Pharmacol 1999;48:694-703.

95. Neumayer HH. Introducing everolimus (Certican) in organ transplantation: an overview of preclinical and early clinical developments. Transplantation 79(9 Suppl): S72-75; 2005.

96. Oberbauer R. Improved renal function in de novo renal transplant patients on sirolimus maintenance therapy following discontinuation of cyclosporine. Ther Drug Monit. 2005 Feb;27(1):7-9.

97. Oberbauer R, Kreis H, Johnson RW, Mota A, Claesson K, Ruiz JC, Wilczek H, Jamieson N, Henriques AC, Paczek L, Chapman J, Burke JT; Rapamune Maintenance Regimen Study Group. Long-term improvement in renal function with sirolimus after early cyclosporine withdrawal in renal transplant recipients: 2-

year results of the Rapamune Maintenance Regimen Study. Transplantation. 2003 Jul 27;76(2):364-70.

98. Ojo AO, Held PJ, Port FK, Wolfe RA, Leichtman AB, Young EW, Arndorfer J, Christensen L, Merion RM. Chronic renal failure after transplantation of a nonrenal organ. N Engl J Med. 2003;349:931-940.

99. Olyaei AJ, de Mattos AM, Bennett WM. Nephrotoxicity of immunosuppressive drugs: new insight and preventive strategies. Curr Opin Crit Care. 2001;7:384-389.

100. Osuna A, Gentil MA, Capdevila L et al. Two doses of daclizumab with delayed introduction of low-dose tacrolimus in elderly recipients of cadaveric renal transplants from donors >55 years of age. Transplant Proc. 2005 Apr; 37(3): 1438-1440.

101. Paoletti E, Amidone M, Cassottana P, Gherzi M, Marsano L, Cannella G. Effect of sirolimus on left ventricular hypertrophy in kidney transplant recipients: a 1-year nonrandomized controlled trial. Am J Kidney Dis. 2008 Aug;52(2):324-30.

102. Pascual J Boletis IN Campistol JM Everolimus (Certican) in renal transplantation: a review of clinical trial data, current usage, and future directions. Transplantation reviews 20: 1-18; 2006

103. Pearson TC, Mulgaonkar S, Patel A, Scandling J, Shidban H, Weir M, Patel D. Efficacy and Safety of Mycophenolate Mofetil (MMF)/Sirolimus (SRL) Maintenance Therapy after Calcineurin Inhibitor (CNI) Withdrawal in Renal Transplant Recipients: Final Results of the Spare-the-Nephron (STN) Trial. ATC 2008; Toronto

104. Pierrat A, Gravier E, Saunders C, Caira MV, Ait-Djafer Z, Legras B, Mallie JP. Predicting GFR in children and adults: a comparison of the Cockcroft-Gault, Schwartz, and modification of diet in renal disease formulas. Kidney Int. 2003 Oct;64(4):1425-1436.

105. Podder H, Stepkowski SM, Napoli KL, Clark J, Verani RR, Chou TC, Kahan BD. Pharmacokinetic interactions augment toxicities of sirolimus/cyclosporine combinations. J Am Soc Nephrol. 2001;12:1059-1071.

106. Ponticelli C, Yussim A, Cambi V, Legendre C, Rizzo G, Salvadori M, Kahn D, Kashi H, Salmela K, Fricke L, Heemann U, Garcia-Martinez J, Lechler R, Prestele H, Girault D; Simulect Phase IV Study Group. A randomized, double-blind trial of basiliximab immunoprophylaxis plus triple therapy in kidney transplant recipients. Transplantation. 2001 Oct 15;72(7):1261-7

107. Rodrigo E, Fernandez-Fresnedo G, Ruiz JC, Pinera C, Heras M, de Francisco AL, Sanz de Castro S, Cotorruelo JG, Zubimendi JA, Arias M. Assessment of glomerular filtration rate in transplant recipients with severe renal insufficiency by Nankivell, Modification of Diet in Renal Disease (MDRD), and Cockroft-Gault equations. Transplant Proc. 2003 Aug; 35(5):1671-1672.

108. Rostaing L, Cantarovich D, Mourad G et al. Corticosteroid-free immunosuppression with tacrolimus, mycophenolate mofetil, and daclizumab induction in renal transplantation. Transplantation 2005 Apr; 79(7): 807-14.

109. Russ G, Segoloni G, Oberbauer R, Legendre C, Mota A, Eris J, Grinyo JM, Friend P, Lawen J, Hartmann A, Schena FP, Lelong M, Burke JT, Neylan JF; for the Rapamune Maintenance Regimen Study Group. Superior outcomes in renal transplantation after early cyclosporine withdrawal and sirolimus maintenance therapy, regardless of baseline renal function. Transplantation. 2005 Nov 15;80(9):1204-11.

110. Salvadori M, Holzer H, de Mattos A, Sollinger H, Arns W, Oppenheimer F, Maca J, Hall M. Enteric-coated mycophenolate sodium is therapeutically equivalent to mycophenolate mofetil in de novo renal transplant patients. Am J Transplant 2003;4:231-236.

111. Satchithananda DK, Parameshwar J, Sharples L, Taylor GJ, McNeil K, Wallwork J, Large SR. The incidence of end-stage renal failure in 17 years of heart transplantation: a single center experience. J Heart Lung Transplant 2002;21:651-657

112. Seckinger J, Sommerer C, Hinkel UP, Hoffmann O, Zeier M, Schwenger V. Switch of immunosuppression from cyclosporine A to everolimus: impact on pulse wave velocity in stable de-novo renal allograft recipients. J Hypertens. 2008 Nov;26(11):2213-9.

113. Shaw LM, Korecka M, Venkataramanan R, Goldberg L, Bloom R, Brayman KL. Mycophenolic acid pharmacodynamics and pharmacokinetics provide a basis for rational monitoring strategies. Am J Transplant. 2003 May; 3(5):534-542.

114. Smak Gregoor PJ, de Sevaux RG, Ligtenberg G, Hoitsma AJ, Hene RJ, Weimar W, Hilbrands LB, van Gelder T. Withdrawal of cyclosporine or prednisone six months after kidney transplantation in patients on triple drug therapy: a randomized, prospective, multicenter study. J Am Soc Nephrol. 2002;13:1365-1373.

115. Smits JM, Persijn GG, van Houwelingen HC, Claas FH, Frei U. Evaluation of the Eurotransplant Senior Program. The results of the first year. Am J Transplant 2002 Aug; 2(7): 664-670.

116. Solez K, Vincenti F, Filo RS. Histopathologic findings from 2-year protocol biopsies from a U.S. multicenter kidney transplant trial comparing tarolimus versus cyclosporine: a report of the FK506 Kidney Transplant Study Group. Transplantation 1998;66:1736-1740.

117. Srinivas TR, Kaplan B, Meier-Kriesche HU. Mycophenolate mofetil in solid-organ transplantation. Expert Opin Pharmacother 2003;4:2325-2345.

118. Suwelack B, Gerhardt U, Hohage H. Withdrawal of cyclosporine or tacrolimus after addition of mycophenolate mofetil in patients with chronic allograft nephropathy. Am J Transplant. 2004 Apr;4(4):655-62.

119. US Renal Data System: 2003 USRDS Annual Data Report. National Institute of Health, National Institute of Diabetes and Digestive and Kidney Diseases, Bethesda, MD, USA, 2003.

120. Valente JF, Hricik D, Weigel K, Seaman D, Knauss T, Siegel CT, Bodziak K, Schulak JA. Comparison of sirolimus vs. mycophenolate mofetil on surgical complications and wound healing in adult kidney transplantation. Am J Transplant. 2003;3:1128-1134.

121. van Gelder T, Balk AH, Zietse R, Hesse C, Mochtar B, Weimar W. Renal insufficiency after heart transplantation: a case-control study. Nephrol Dial Transplant 1998;13: 2322-2326.

122. Velosa JA, Larson TS, Gloor JM, Stegall MD. Cyclosporine elimination in the presence of TOR inhibitors: effects on renal function, acute rejection, and safety. Am J Kidney Dis. 2001;38(4 Suppl 2):S3-S10.

123. Vincenti F, Ramos E, Brattstrom C, Cho S, Ekberg H, Grinyo J, Johnson R, Kuypers D, Stuart F, Khanna A, Navarro M, Nashan B. Multicenter trial exploring calcineurin inhibitors avoidance in renal transplantation. Transplantation. 2001;71:1282-1287

124. Vincenti F, Friman S, Scheuermann E et al. DIRECT (Diabetes Incidence after Renal Transplantation: Neoral C Monitoring Versus Tacrolimus) Investigators. Results of an international, randomized trial comparing glucose metabolism disorders and outcome with cyclosporine versus tacrolimus. Am J Transplant. 2007 Jun; 7(6): 1506-14.

125. Vincenti F, Schena FP, Paraskevas S, Hauser IA, Walker RG, Grinyo J; FREEDOM Study Group. A randomized, multicenter study of steroid avoidance, early steroid withdrawal or standard steroid therapy in kidney transplant recipients. Am J Transplant. 2008 Feb;8(2):307-16.

126. Vitko S, Tedesco H, Eris J, Pascual J, Whelchel J, Magee JC, Campbell S, Civati G, Bourbigot B, Filho GA, Leione J, Garcia VD, Rigotti P, Esmeraldo R, Cambi V, Haas T, Jappe A, Bernhardt P, Geissler J, Cretin J. Everolimus with optimized cyclosporine dosing in renal transplant recipients: 6-month safety and efficacy results of two randomized studies. Am J Transplant 2004;4:626-635.

127. Vitko S, Margreiter R, Weimar W, et al. For the RAD B201 Study Group. Three-year efficacy and safety results from a study of everolimus versus mycophenolate mofetil in de novo renal transplant patients. Am J Transplant. 5:2521-2530; 2005.

128. Vitko S, Wlodarczyk Z, Kyllonen L, Czajkowski Z, Margreiter R, Backman L, Perner F, Rigotti P, Jaques B, Abramowicz D, Kessler M, Sanchez-Plumed J, Rostaing L, Rodger RS, Donati D, Vanrenterghem Y. Tacrolimus combined with two different dosages of sirolimus in kidney transplantation: results of a multicenter study. Am J Transplant. 2006 Mar;6(3):531-8.

129. Webster AC, Woodroffe RC, Taylor RS, Chapman JR, Craig JC. Tacrolimus versus ciclosporin as primary immunosuppression for kidney transplant recipients:

meta-analysis and meta-regression of randomized trial data. BMJ 2005 Oct; 331(7520): 810.

130. Wilson PW, D'Agostino RB, Levy D, Belanger AM, Silbershatz H, Kannel WB. Prediction of coronary heart disease using risk factor categories. Circulation 1998 May; 97(18):1837-1847.

131. Wheeler DC, Steiger J. Evolution and etiology of cardiovascular diseases in renal transplant recipients. Transplantation. 2000 Dec 15;70(11 Suppl):SS41-5.

132. Yakupoglu YK, Kahan BD. Sirolimus: a current perspective. Exp Clin Transplant. 2003 Jun;1(1):8-18.

# Appendix 1: Administrative procedures

## Regulatory and ethical compliance

This clinical study was designed and shall be implemented and reported in accordance with the ICH Harmonized Tripartite Guidelines for Good Clinical Practice, with applicable local regulations (including European Directive 2001/83/EC and US Code of Federal Regulations Title 21), and with the ethical principles laid down in the Declaration of Helsinki.

## Responsibilities of the investigator and IRB/IEC/REB

The protocol and the proposed informed consent form must be reviewed and approved by a properly constituted Institutional Review Board/Independent Ethics Committee/Research Ethics Board (IRB/IEC/REB) before study start. A signed and dated statement that the protocol and informed consent have been approved by the IRB/IEC/REB must be given to Novartis before study initiation. Prior to study start, the investigator is required to sign a protocol signature page confirming his/her agreement to conduct the study in accordance with these documents and all of the instructions and procedures found in this protocol and to give access to all relevant data and records to Novartis monitors, auditors, Novartis Clinical Quality Assurance representatives, designated agents of Novartis, and regulatory authorities as required.

## Informed consent

Eligible patients may only be included in the study after providing written (witnessed, where required by law or regulation), IRB/IEC/REB-approved informed consent, or, if incapable of doing so, after such consent has been provided by a legally acceptable representative of the patient. Informed consent must be obtained before conducting any study-specific procedures (i.e., all of the procedures described in the protocol). The process of obtaining informed consent should be documented in the patient source documents.

The informed consent form that complies with regulatory requirements and is considered appropriate for this study is provided separately. Any changes to the consent form suggested by the investigator must be agreed to by Novartis before submission to the IRB/IEC/REB, and a copy of the approved version must be provided to the Novartis monitor after IRB/IEC/REB approval.

## Amendments to the protocol

Any change or addition to the protocol can only be made in a written protocol amendment that must be approved by Novartis, Health Authorities where required, and the IRB/IEC/REB. Only amendments that are required for patient safety may be implemented prior to IRB/IEC/REB approval. Notwithstanding the need for approval of formal protocol amendments, the investigator is expected to take any immediate action required for the safety of any patient included in this study, even if this action

represents a deviation from the protocol. In such cases, Novartis should be notified of this action and the IRB/IEC/REB at the study site should be informed within 10 working days.

### Discontinuation of the study

Novartis reserves the right to discontinue this study under the conditions specified in the clinical trial agreement.

### Study drug supply and resupply, storage, and tracking

Study drugs must be received by a designated person at the study site, handled and stored safely and properly, and kept in a secured location to which only the investigator and designated assistants have access. Upon receipt, the study medication should be stored according to the instructions specified on the drug labels. Clinical supplies are to be dispensed only in accordance with the protocol.

Medication labels will be in the German and comply with the legal requirements. They will include storage conditions for the drug and the randomization number but no information about the patient.

The investigator must maintain an accurate record of the shipment and dispensing of study drug in a drug accountability ledger. Drug accountability will be noted by the field monitor during site visits and at the completion of the trial. Patients will be asked to return all unused study drug and packaging at the end of the study or at the time of study drug discontinuation.

At the conclusion of the study, and, as appropriate during the course of the study, the investigator will return all used and unused study drug, packaging, drug labels, and a copy of the completed drug accountability ledger to the Novartis monitor or to the Novartis address provided in the investigator folder at each site.

# Appendix 2: Notable laboratory value criteria, special methods and scales

| Laboratory variable | Standard units | SI units |
|---|---|---|
| **Liver function and related variables** | | |
| SGOT (AST) | ≥3 × ULN | ≥3 × ULN |
| SGPT (ALT) | ≥3 × ULN | ≥3 × ULN |
| Bilirubin | ≥3 × ULN | ≥3 × ULN |
| **Renal function, metabolic and electrolyte variables** | | |
| Urea | None | None |
| Creatinine | Day 8 or after:<br>>30% above value from preceding visit<br>**or**<br>Day 8 to Wk 4: ≥ 4 mg/dL,<br>After Wk 4: ≥ 3 mg/dL | Day 8 or after:<br>>30% above value from preceding visit<br>**or**<br>Day 8 to Wk 4: ≥ 354 µmol/L,<br>After Wk 4: ≥ 265 µmol/L |
| Uric acid | M ≥12 mg/dL<br>F ≥9 mg/dL | M ≥714 µmol/L<br>F ≥535 µmol/L |
| Glucose | <45 mg/dL<br>>250 mg/dL | <2.5 mmol/L<br>>13.9 mmol/L |
| Cholesterol | ≥350 mg/dL | ≥9.1 mmol/L |
| Triglycerides | ≥750mg/dL | ≥8.5 mmol/L |
| CPK (MB) | None | None |
| Potassium | ≤3.0 mEq/L<br>≥6.0 mEq/L | ≤3 mmol/L<br>≥6 mmol/L |
| Calcium | ≤6mg/dL<br>≥13mg/dL | ≤1.5mmol/L<br>≥3.2mmol/L |
| **Hematology variables** | | |
| Hemoglobin | <7 g/dL | <4.39 mmol/L |
| Platelets (thrombocytes) | <50 k/mm$^3$<br>≥700 k/mm$^3$ | <50 × 10$^9$/L<br>≥700 ×10$^9$/L |
| Leukocytes (WBCs) | ≤2.0 k/mm$^3$<br>≥16 k/mm$^3$ | ≤2.0 × 10$^9$/L<br>≥16 × 10$^9$/L |
| **Hematology variables: differential** | | |
| Granulocytes (poly, neutrophils) | ≤1,000/mm$^3$ | ≤1 x 10$^9$/L |
| Eosinophils | ≥12% | ≥12% |
| Lymphocytes | ≤1,000/mm$^3$ | ≤1× 10$^9$/L |

| **Harmonized notable vital signs (and weight)** | |
|---|---|
| **Vital sign variables** | **Notable criteria** |
| Pulse (beats/min.) | None |
| Systolic BP (mm/Hg) | Either an increase of ≥30 that results in ≥180 or >200(mm/Hg)<br>or<br>Either a decrease of ≥30 that results in ≤90 or <75 (mm/Hg) |
| Diastolic BP (mm/Hg) | Either an increase of ≥20 that results in ≥105 or >115 (mm/Hg)<br>or<br>Either a decrease of ≥20 that results in ≤50 or <40 (mm/Hg) |
| Weight (Kg) | None |

# Appendix 3: Possible drug interactions

All of the individual drugs cited below are well substantiated to interact with cyclosporine. In addition, concomitant non-steroidal anti-inflammatory drugs, particularly in the setting of dehydration, may potentiate renal dysfunction.

## Drugs that may potentiate renal dysfunction

| Antibiotics | Antifungals | Gastrointestinal agents |
|---|---|---|
| Gentamicin<br>Tobramycin<br>Vancomycin<br>Trimethoprim with-sulfamethoxazole | Amphotericin B<br>Ketoconazole | Cimetidine<br>Ranitidine |
| **Antineoplastics** | **Anti-inflammatory drugs** | **Immunosuppressives** |
| Melphalan | Azapropazon<br>Diclofenac<br>Naproxen<br>Sulindac | Tacrolimus |

## Drugs that alter cyclosporine concentrations

Cyclosporine is extensively metabolized. Cyclosporine concentrations may be influenced by drugs that affect microsomal enzymes, particularly cytochrome P-450 III-A. Substances that inhibit this enzyme could decrease metabolism and increase cyclosporine concentrations. Substances that are inducers of cytochrome P-450 activity could increase metabolism and decrease cyclosporine concentrations. Monitoring of circulating cyclosporine concentrations and appropriate Sandimmun® Optoral dosage adjustments are essential when these drugs are used concomitantly.

## Drugs that increase cyclosporine concentrations

| Calcium channel blockers | Antifungals | Antibiotics |
|---|---|---|
| Diltiazem<br>Nicardipine<br>Verapamil | Ketoconazole<br>Fluconazole<br>Itraconazole | Clarithromycin<br>Erythromycin |
| **Glucocorticoids** | **Other drugs** | |
| | Allopurinol<br>Bromocriptine<br>Danazol<br>Metoclopramide | |

Grapefruit and grapefruit juice affect cyclosporine metabolism, increasing blood concentrations of cyclosporine, thus should be avoided.

### Drugs that decrease cyclosporine concentrations

| Antibiotics | Anticonvulsants | Other drugs |
|---|---|---|
| Nafcillin<br>Rifampin | Carbamazepine<br>Phenobarbital<br>Phenytoin | Octreotide<br>Ticlopidine<br>Troglitazone |

Rifabutin is known to increase the metabolism of other drugs metabolized by the cytochrome P-450 system. The interaction between rifabutin and cyclosporine has not been studied. Care should be exercised when these two drugs are administered concomitantly.

### Other drug interactions

Reduced clearance of prednisolone, digoxin and lovastatin and simvastatin has been observed when these drugs are administered with cyclosporine. In addition, a decrease in the apparent volume of distribution of digoxin has been reported after cyclosporine administration. Severe digitalis toxicity has been seen within days of starting cyclosporine in several patients taking digoxin. Cyclosporine should not be used with potassium-sparing diuretics because hyperkalemia can occur.

During treatment with cyclosporine, vaccination may be less effective. The use of live vaccines should be avoided. Myositis has occurred with concomitant lovastatin and simvastatin, frequent gingival hyperplasia with nifedipine, and convulsions with high dose methylprednisolone.

### Population screen for co-medications influencing everolimus (Certican®)

Patients concomitantly receivingerythromycin antibiotics (erythromycin or azithromycin) had an average 20% lower everolimus CL/F than those not receiving these co-medications. A single patient receiving intraconazole had a 3-fold lower everolimus CL/F compares with the population average. These observations indicate that potent inhibitors of CYP3A can decrease the clearance of everolimus and increase its blood levels.

Additional drugs or drug classes explored in the population model for which no influence on everolimus CL/F was detected were: atorvastatin (n=74 patients), pravastatin (n=41), simvastatin (n=18), gemfibrozil (n=10), quinolone antibiotics (n=124), Bactrim® (n=450), and various calcium-channel blockers (dihydropyridines, n=267, diltiazem, n=22 and verapamil, n=5).

# Appendix 4: Dose Reduction of Certican® and Myfortic®

Dose reduction or temporary interruption may be performed for Myfortic® or Certican®. However, Certican® should be continued on a stable dose level, if possible, and reduced or interrupted only if the symptoms do not respond to the reduction of the Myfortic® dosage.

Implementation of dose reduction will be based on thrombocytopenia, leukopenia, neutropenia, hyperscholesterolemia, hypertriglyceridemia, or other adverse events which are suspected to be related to study medication, and in the opinion of the investigator, are clinically warranted. The following guidelines should be used for both dose reduction and restarting or increasing the dose of study medication back to original levels.

## Dose Reduction Guidelines

### Platelets

- platelet count < 100,000/mm³ dose **may** be reduced at the discretion of the investigator
- platelet count < 75,000/mm³ a second dose reduction should be **considered**
- platelet count < 50,000/mm³ **MANDATORY** interruption of medication

### WBC

- WBC < 2,500/mm³ dose may be reduced at the discretion of the investigator A second dose reduction may be implemented if the WBC continues to fall

| Dose Reduction | Myfortic® |
|---|---|
| **Maintenance dose** | 2 tablets 360 mg b.i.d. |
| **Initial dose reduction** | 1 tablets 360 mg + 1 tablets 180 mg b.i.d. |
| **Second dose reduction** | 2 tablets 180 mg b.i.d. |

## Increase or restart of study medication after a drop in platelets / WBC

### Increase dose of study medication following a single dose reduction

After a single dose reduction, full dose of study medications **must** restart if:

- platelet count > 100,000/$mm^3$ (at least 3 days)
- WBC > 3,500/$mm^3$ (at least 3 days)

### Increase dose of study medication following a double dose reduction

After a double dose reduction the mid dose of study medications **may** be restarted if:

- platelet count > 75,000/$mm^3$ (at least 3 days)
- WBC > 3,500/$mm^3$ (stable) (at least 3 days)

the full dose of study medications **must** be restarted if

- platelet count > 100,000/$mm^3$ (at least 7 days)
- WBC > 3,500/$mm^3$ (stable) (at least 7 days)

### Restart of dose of study medication following an interruption

After an interruption of study medications (due to a decrease in platelets/WBC) study medication **may** be restarted at the lowest dose if:

- platelet count > 50,000/$mm^3$ (at least 3 days)
- WBC > 3,500/$mm^3$ (stable) (at least 3 days)

the mid dose of study medications **may** be restarted if:

- platelet count > 75,000/$mm^3$ (at least 3 days)
- WBC > 3,500/$mm^3$ (stable) (at least 3 days)

the full dose of study medications **must** be restarted if

- platelet count > 100,000/$mm^3$ (at least 7 days)
- WBC > 3,500/$mm^3$ (stable) (at least 7 days)

## Absolute neutrophil count cutoff levels

A decrease in absolute neutrophil counts (ANC) to less than 1,000/mm³ should be avoided during the study. The investigator should use his/her discretion to reduce the dose of non-study medication known to be associated with neutropenia or leukopenia (e.g., ganciclovir, trimethoprim-sulfamethoxazole) before considering initiating a reduction in the dose of study medication.

Recommendations for adjusting the dose of study medication in case of a decrease in absolute neutrophil counts are as follows:

| | |
|---|---|
| ANC < 1,500/mm³: | Consider dose reduction (per investigator discretion) |
| ANC < 1,000/mm³: | Mandatory interruption of study medication |

Restart of study medication after a drop in ANC that required interruption of study medication

| | |
|---|---|
| ANC > 1,500/mm³ for 7 days: | Restart full dose of study medication |

## Lipid cutoff levels

Evaluations of plasma cholesterol and/or triglyceride levels can result from a number of drugs commonly used in renal transplantation, including study medication, steroids, cyclosporine, and other drugs.

If plasma cholesterol and/or triglyceride levels are found to be elevated, the dosing of all concomitant medication potentially affecting cholesterol and/or triglyceride levels should be re-considered and optimized as appropritate, in addition to providing dietary advice.

If plasma cholesterol and/or triglyceride levels are still found to be elevated and the elevation is confirmed by a second measurement (performed within two weeks under fasting conditions [last meal ≥ 8 hours prior to sampling]) treatment based on the National Cholesterol Education Program Adult Treatment Panel II (NCEP ATP II) guidelines (see Appendix 3) should be instituted. The patient’s CHD risk should be assessed as the NCEP ATP II guidelines are based on presence/absence of CHD and number of CHD risk factors.

Treatment of elevated lipids should be maximized prior to dose reduction or interruption of study medication.

Based on the National Cholesterol Education Program (NCEP) guidelines (Appendix 3) the recommendations for treatment of hyperlipidemia prior to reduction/interruption of study medication are as follows:

If Total Cholesterol ≥ **250** mg/dL (6.5 mmol/L) and/or

LDL-C ≥ **160** mg/dL (4.1 mmol/L):

***Review Concomitant Medication that may potentially contribute to lipid elevations and provide Dietary Instruction (Registered Dietitian)***

↓

If Total Cholesterol still ≥ **250** mg/dL (6.5 mmol/L) and/or

LDL-C still ≥ **160** mg/dL (4.1 mmol/L):

***Institute Statin (HMG CoA Reductase Inhibitor, e.g. Lescol®) Therapy Unless Contraindicated***

↓

If Total Cholesterol still ≥ **250** mg/dL (6.5 mmol/L) and/or

LDL-C still ≥ **160** mg/dL (4.1 mmol/L):

***Optimize Statin Therapy and/or Implement Other Lipid Lowering Therapy***

↓

If Total Cholesterol still ≥ **250** mg/dL (6.5 mmol/L) and/or

LDL-C still ≥ **160** mg/dL (4.1 mmol/L) and/or

TG ≥ **600** mg/dL (6.9 mmol/L):

***Consider Fibrate Therapy for Treatment of Hypertriglyceridemia***

↓

If Total Cholesterol still ≥ **250** mg/dL (6.5 mmol/L) and/or

LDL-C still ≥ **160** mg/dL (4.1 mmol/L) and/or

TG still ≥ **600** mg/dL (6.9 mmol/L):

***Consider Dose Reduction / Interruption***

↓

Recommendations for adjusting the dose of study medication in case of hypercholesterolemia [Total Cholesterol ≥ 250 mg/dL (6.5 mmol/L) and/or LDL-Cholesterol ≥ 160 mg/dL (4.1 mmol/L)] and/or hypertriglyceridemia [TG ≥ 600 mg/dL (6.9 mmol/L)], after treatment of elevated lipids had been maximized, are as follows:

**Total Cholesterol** and/or **Triglycerides**

| | | |
|---|---|---|
| = 250 – 750 mg/dL<br>= 6.5 – 19.4 mmol/L | = 600 – 1000 mg/dL<br>= 6.9 – 11.4 mmol/L | Dose reduction should be considered |
| >750 mg/dL<br>> 19.4 mmol/L | > 1000 mg/dL<br>> 11.4 mmol/L | Study medication should be interrupted |

**NB:** If a patient is restarting the dose of study medication, the investigator my use his/her discretion with regard to the adjusted dose of study medication, i.e., they are not required to increase doses back to full dose regardless of the platelet or WBC levels, the ANC levels, and/or the cholesterol or triglyceride levels.

## Handling of Gastrointestinal Problems

In case of gastrointestinal problems that are caused by the treatment with Myfortic® or Certican® the first step should be to divide the total daily dose in 3 to 4 single dosages. In case of persisting gastrointestinal complaints, the Myfortic® dosage should be reduced in steps of 360 mg/day until the event resolves.

In the event of gastrointestinal problems that are caused by any other reason (e.g. infections) the medication dosage of Myfortic® and Certican®, if applied, should be maintained. Other appropriate actions should be taken based on the investigators' experience and discretion.

# Appendix 5: National Cholesterol Education Program Guidelines[1]

| Total cholesterol | |
|---|---|
| Under 200 mg/dL | Desirable |
| 200 – 239 mg/dL | Borderline-high |
| 240 mg/dL and over | High |

| HDL cholesterol | |
|---|---|
| Under 35 mg/dL | Low |

Treatment based on LDL cholesterol

| *Diet Therapy* | *Initiation Level* | *LDL Goal* |
|---|---|---|
| Without CHD and with fewer than 2 risk factors* | 160 mg/dL or over | Under 160 mg/dL |
| Without CHD and with 2 or more risk factors* | 130 mg/dL or over | Under 130 mg/dL |
| With CHD | Over 100 mg/dl | 100 mg/dL or under |

| *Drug Treatment* | *Initiation Level* | *LDL Goal* |
|---|---|---|
| Without CHD and with fewer than 2 risk factors* | 190 mg/dL or over | Under 160 mg/dL |
| Without CHD and with 2 or more risk factors* | 160 mg/dL or over | Under 130 mg/dL |
| With CHD | Over 130 mg/dl | 100 mg/dL or under |

***CHD Risk Factors:**

Positive

- Age: Men ≥ 45 yrs
  Women ≥ 55 yrs or premature menopause without estrogen replacement therapy
- Family history of premature CHD (definite myocardial infarction or sudden death before 55 yrs of age in father or other male first-degree relative, of before 65 yrs of age in mother or other female first-degree relative)
- Current cigarette smoking
- Hypertension (Blood pressure ≥ 140/90 mmHg [confirmed by measurements on several occasions] or taking antihypertensive medication)
- HDL Cholesterol < 35 mg/dL (0.6 mmol/L)
  (confirmed by measurements on several occasions)
- Diabetes mellitus

Negative:

- HDL Cholesterol ≥ 60 mg/dL (1.6 mmol/L)
  (confirmed by measurements on several occasions)

[1] Data from Expert Panel on Detection, Evaluation, and Treatment of High Blood Cholesterol in Adults: Summary of the second report of the National Cholesterol in Adults [Adult Treatment Panel II] JAMA 269[23]:3015-3023, 1993

# Appendix 6: Definition of renal allograft rejection

## Diagnostic categories for renal allograft biopsies (Banff'07 update)

1. **Normal**
2. **Antibody-mediated changes** (may coincidence with categories 3,4 and 5 and 6)

   Due to documentation of circulating antidonor antibody, and C4d* or allograft pathology

   *C4d deposition without morphologic evidence of active rejection*

   C4d+, presence of circulating antidonor antibodies, no signs of acute or chronic TCMR or ABMR (i.e. g0, cg0, ptc0, no ptc lamination). Cases with simultaneous borderline changes or ATN are considered as indeterminate

   *Acute antibody-mediated rejection*

   C4d+, presence of circulating antidonor antibodies, morphologic evidence of acute tissue injury, such as (Type/Grade):

   I. ATN-like minimal inflammation

   II. Capillary and or glomerular inflammation (ptc/g > 0) and/or thrombose

   III. Arterial-v3

   *Chronic active antibody-mediated rejection***

   C4d+, presence of circulating antidonor antibodies, morphologic evidence of chronic tissue injury, such as glomerular double contours and/or peritubular capillary basement membrane multilayering and/or interstitial fibrosis/tubular atrophy and/or fibrous intimal thickening in arteries
3. **Borderline changes:** Suspicious for acute T-cell mediated rejection (may coincidence with categories 2 and 5 and 6)

   This category is used when no intimal arteritis is present, but there are foci of mild tubulitis

   (t1, t2 or t3) with minor interstitial infiltration (i0 or i1) or interstitial infiltration (i2, i3) with mild (t1) tubulitis
4. **T-cell mediated rejection** (TCMR, may coincidence with categories 2 and 5 and 6)

   *Acute T-cell mediated rejection (Type/Grade)*

   GRADE IA - Cases with significant interstitial infiltration (>25% of parenchyma affected, i2 or i3) and foci of moderate tubulitis (t2).

   GRADE IB - Cases with significant interstitial infiltration (>25% of parenchym affected, i2 or i3) and foci of severe tubulitis (t3 ).

   GRADE IIA - Cases with mild-to-moderate intimal arteritis (v1).

   GRADE IIB - Cases with severe intimal arteritis comprising >25% of the luminal area (v2).

   GRADE III - Cases with "transmural" arteritis and/or fibrinoid change and necrosis of medial smooth muscle cells with accompanying lymphocyte inflammation (v3).

   *Chronic active T-cell-mediated rejection*

"chronic allograft arteriopathy" (arterial intimal fibrosis with mononuclear cell infiltration in fibrosis, formation of neo-intimal)

5. **Interstitial fibrosis and tubular atrophy**, no evidence of any specific ethiology

   (may include nonspecific vascular and glomerular sclerosis, but severity graded by tubulointerstitial features)

   Grade

   I. Mild interstitial fibrosis and tubular atrophy (<25% of cortical area)

   II. Moderate interstitial fibrosis and tubular atrophy (25-50% of cortical area)

   III. Severe interstitial fibrosis and tubular atrophy/loss (>50% of cortical area)

6. **Other:** Changes not considered to be due to rejection – acute and/or chronic (for diagnoses see Tabel 14 in Racusen et al., 1997; may include isolated g, cg or cv lesions and coincide with categories 2, 3, 4 and 5)

* Please refer to Scoring of C4d staining

** Suspicious for antibody-mediated rejection if C4d (in the presence of antibody) or alloantibody (C4d+) not demonstrated in the presence of morphologic evidence of tissue injury.

## Specimen adequacy and lesion scoring (Banff '07)

Numerical codes, specimen adequacy and minimum sampling standards

### Quantitative criteria for tubulitis ("t") score (assumes minimum sampling)

| | |
|---|---|
| t0 | No mononuclear cells in tubules |
| t1 | Foci with 1-4 cells/tubular cross section or 10 tubular cells |
| t2 | Foci with 5-10 cells/tubular cross section |
| t3 | Foci with >10 cells/tubular cross section, or the presence of at least two areas of tubular basement membrane destruction accompanied by i2/i3 and t2 tubulitis elsewhere in the biopsy |

### Quantitative criteria for mononuclear cell interstitial inflammation ("i")

| | |
|---|---|
| i0 | No or trivial interstitial inflammation (<10% unscarred parenchyma) |
| i1 | 10%-25% of parenchyma inflamed cells |
| i2 | 26%-50% of parenchyma inflamed |
| i3 | >50% of parenchyma inflamed |

Indicate presence of remarkable numbers of eosinophils, polys, or plasma cells (specify which) with an asterisk* on i

### Quantitative criteria for the early type of allograft glomerulitis ("g")

| | |
|---|---|
| g0 | No glomerulitis |
| g1 | Glomerulitis in a minority of glomeruli |
| g2 | Segmental or global glomerulitis in about 25%-75% of glomeruli |
| g3 | Glomerulitis (mostly global) in all or almost all glomeruli |

## Quantitative criteria for arteriolar hyaline thickening ("ah")

| | |
|---|---|
| ah0 | No PAS-positive hyaline thickening |
| ah1 | Mild to moderate PAS-positive hyaline thickening in at least one arteriole |
| ah2 | Moderate to severe PAS-positive hyaline thickening in more than one arteriole |
| ah3 | Severe PAS-positive hyaline thickening in many arterioles |

Indicate arteriolitis (significance unknown) by an asterisk on ah

## Quantitative criteria for intimal arteritis ("v")

| | |
|---|---|
| v0 | No arteritis |
| v1 | Mild to moderate arteritis in at least one arterial cross section |
| v2 | Severe intimal arteritis with at least 25% luminal area lost in at least one arterial cross section |
| v3 | Arterial fibrinoid change and/or transmural arteritis with medial smooth muscle necrosis |

Note number of arteries present and number affected. Indicate infarction and/or interstitial hemorrhage by an asterisk (with and level "v" score)

## Quantitative criteria for allograft glomerulopathy ("cg")

| | |
|---|---|
| cg0 | No glomerulopathy, double contours in 10% of peripheral capillary loops |
| cg1 | BM thickening with double contours affecting up to 25% of peripheral capillary loops in nonsclerotic glomeruli |
| cg2 | Increased severity of changes described above affecting 26%-50% of peripheral capillary loops in nonsclerotic glomeruli |
| cg3 | Severe changes of classic transplant glomerulopathy with double contours affecting more than 50% of peripheral capillary loops in nonsclerotic glomeruli |

Note number of glomeruli and number sclerotic

## Quantitative criteria for interstitial fibrosis ("ci")

| | |
|---|---|
| ci0 | Interstitial fibrosis tissue in up to 5% of cortical area |
| ci1 | Mild interstitial fibrosis tissue in 6%-25% of cortical area |
| ci2 | Moderate interstitial fibrosis of 26%-50% of cortical area |
| ci3 | Moderate interstitial fibrosis of >50% of cortical area |

## Quantitative criteria for tubular atrophy ("ct")

| | |
|---|---|
| ct0 | No tubular atrophy |
| ct1 | Tubular atrophy in up to 25% of the area of cortical tubules |
| ct2 | Tubular atrophy involving 26%-50% of the area of cortical tubules |
| ct3 | Tubular atrophy of >50% of the area of cortical tubules |

## Quantitative criteria for fibrous intimal thickening ("cv")

| | |
|---|---|
| cv0 | No chronic vascular change |
| cv1 | Vascular narrowing of up to 25% luminal area by fibrointimal thickening of arteries ± breach of internal elastic lamina or presence of foam cells or occasional mononuclear cells* |

| | |
|---|---|
| cv2 | Increased severity of changes described above with 26%-50% narrowing of vascular luminal area* |
| cv3 | Severe vascular changes with >50% narrowing of vascular luminal area |

*In most severely affected vessel. Note if lesions characteristic of chronic rejection (elastica breaks, inflammatory cells in fibrosis) are seen

## Quantitative criteria for mesangial matrix increase (“mm”)*

| | |
|---|---|
| mm0 | No mesangial matrix increase |
| mm1 | Up to 25% of nonsclerotic glomeruli affected (at least moderate matrix increase) |
| mm2 | 26%-50% nonsclerotic glomeruli affected (at least moderate matrix increase) |
| mm3 | >50% nonsclerotic glomeruli affected (at least moderate matrix increase) |

*The threshold criterion for the moderately increased “mm” is the expanded mesangial interspace between adjacent capillaries. If the width of the interspace exceeds two mesangial cells on the average in at least two glomerular lobules the “mm” is moderately increased.

## Quantitative criteria for peritubular capillaritis (“ptc”)*

| | |
|---|---|
| ptc0 | No significant cortical ptc, or < 10% of PTCs with inflammation |
| ptc1 | ≥10% of cortical peritubular capillaries with capillaritis, with max 3 to 4 luminal inflammatory cells |
| ptc2 | ≥10% of cortical peritubular capillaries with capillaritis, with max 5 to 10 luminal inflammatory cells |
| ptc3 | ≥10% of cortical peritubular capillaries with capillaritis, with max >10 luminal inflammatory cells |

*It is recommended that one comment on the composition (mononuclear cells vs. neutrophils) and extent (focal, ≤50% vs.diffuse >50%) of peritubular capillaritis.

## Scoring of C4d staining (% of biopsy or 5 high-power fields)

| | |
|---|---|
| C4d0 | Negative (0%) |
| C4d1 | Minimal C4d stain/detection (1<10%) |
| C4d2 | Focal C4d stain/positive (10-50%) |
| C4d3 | Diffuse C4d stain/positive (>50%) |

The recommended format of report is a descriptive narrative signout followed by numerical codes in parentheses. Categorization should in the first instance be based solely on pathologic changes then integrated with clinical data as a second step. More than one diagnostic category may be used if appropriate.

## Specimen adequacy (a necessary prerequisite for numeric coding)

| | |
|---|---|
| Unsatisfactory | No glomeruli or arteries |
| Marginal | 1-9 glomeruli with an artery |

| | |
|---|---|
| Adequate | 10 or more glomeruli with at least two arteries |
| **Minimum sampling** | |
| 7 slides | 3 H &E, 3 PAS or silver stains, and 1 trichrome |

## References relating to methods

1. Solez K, Axelsen RA, Benediktsson H, et al. International standardization of criteria for the histologic diagnosis of renal allograft rejection: The Banff working classification of kidney transplant pathology. Kidney Internat 1993;44:411-22.

2. Solez K, et al. Report of the third Banff conference on allograft pathology (July 20-24, 1995) on classification and lesion scoring in renal allograft pathology. Transpl Proc 1996;28(1):441-4.

3. Racusen LC, et al. The Banff 97 working classification of renal allograft pathology Kidney Int. 1999 Feb;55(2):713-23.

4. Solez K, et al. Banff 07 classification of renal allograft pathology: updates and future directions. Am J Transplant. 2008 Apr;8(4):753-60. Epub 2008 Feb 19

# Appendix 7: Coronary Disease Risk Prediciton Score (Framingham Score)

### Coronary Disease Risk Prediction Score Sheet for Men Based on LDL Cholesterol Level

**Step 1**

| Age | |
|---|---|
| Years | Points |
| 30-34 | -1 |
| 35-39 | 0 |
| 40-44 | 1 |
| 45-49 | 2 |
| 50-54 | 3 |
| 55-59 | 4 |
| 60-64 | 5 |
| 65-69 | 6 |
| 70-74 | 7 |

**Step 2**

| LDL - Cholesterol | | |
|---|---|---|
| (mg/dl) | (mmol/L) | Points |
| <100 | ≤2.59 | -3 |
| 100-129 | 2.60-3.36 | 0 |
| 130-159 | 3.37-4.14 | 0 |
| 160-189 | 4.15-4.91 | 1 |
| ≥190 | ≥4.92 | 2 |

| Key | |
|---|---|
| Color | Risk |
| green | Very low |
| white | Low |
| yellow | Moderate |
| rose | High |
| red | Very high |

**Step 3**

| HDL - Cholesterol | | |
|---|---|---|
| (mg/dl) | (mmol/L) | Points |
| <35 | ≤0.90 | 2 |
| 35-44 | 0.91-1.16 | 1 |
| 45-49 | 1.17-1.29 | 0 |
| 50-59 | 1.30-1.55 | 0 |
| ≥60 | ≥1.56 | -1 |

**Step 4**

| Blood Pressure | | | | | |
|---|---|---|---|---|---|
| Systolic | Diastolic (mmHg) | | | | |
| (mmHg) | <80 | 80-84 | 85-89 | 90-99 | ≥100 |
| <120 | 0 | | | | |
| 120-129 | | 0 pts | | | |
| 130-139 | | | 1 | | |
| 140-159 | | | | 2 | |
| ≥160 | | | | | 3 pts |

Note: When systolic and diastolic pressures provide different estimates for point scores, use the higher number

**Step 5**

| Diabetes | |
|---|---|
| | Points |
| No | 0 |
| Yes | 2 |

**Step 6**

| Smoker | |
|---|---|
| | Points |
| No | 0 |
| Yes | 2 |

Risk estimates were derived from the experience of the NHLBI's Framingham Heart Study, a predominantly Caucasian population in Massachusetts, USA

**Step 7** (sum from steps 1-6)

| Adding up the points | |
|---|---|
| Age | ______ |
| LDL Cholesterol | ______ |
| HDL Cholesterol | ______ |
| Blood Pressure | ______ |
| Diabetes | ______ |
| Smoker | ______ |
| Point Total | ______ |

**Step 8** (determine CHD risk from point total)

| CHD Risk | |
|---|---|
| Point Total | 10 Yr CHD Risk |
| ≤-3 | 1% |
| -2 | 2% |
| -1 | 2% |
| 0 | 3% |
| 1 | 4% |
| 2 | 4% |
| 3 | 6% |
| 4 | 7% |
| 5 | 9% |
| 6 | 11% |
| 7 | 14% |
| 8 | 18% |
| 9 | 22% |
| 10 | 27% |
| 11 | 33% |
| 12 | 40% |
| 13 | 47% |
| ≥14 | ≥56% |

**Step 9** (compare to man of the same age)

| Comparative Risk | | |
|---|---|---|
| Age (years) | Average 10 Yr CHD Risk | Low* 10 Yr CHD Risk |
| 30-34 | 3% | 2% |
| 35-39 | 5% | 3% |
| 40-44 | 7% | 4% |
| 45-49 | 11% | 4% |
| 50-54 | 14% | 6% |
| 55-59 | 16% | 7% |
| 60-64 | 21% | 9% |
| 65-69 | 25% | 11% |
| 70-74 | 30% | 14% |

*Low risk was calculated for a man the same age, normal blood pressure, LDL cholesterol 100-129 mg/dL, HDL cholesterol 45 mg/dL, non-smoker, no diabetes

## Coronary Disease Risk Prediction Score Sheet for Women Based on LDL Cholesterol Level

**Step 1**

| Age | |
|---|---|
| **Years** | **Points** |
| 30-34 | -9 |
| 35-39 | -4 |
| 40-44 | 0 |
| 45-49 | 3 |
| 50-54 | 6 |
| 55-59 | 7 |
| 60-64 | 8 |
| 65-69 | 8 |
| 70-74 | 8 |

**Step 2**

| LDL - Cholesterol | | |
|---|---|---|
| **(mg/dl)** | **(mmol/L)** | **Points** |
| <100 | ≤2.59 | -2 |
| 100-129 | 2.60-3.36 | 0 |
| 130-159 | 3.37-4.14 | 0 |
| 160-189 | 4.15-4.91 | 2 |
| ≥190 | ≥4.92 | 2 |

| Key | |
|---|---|
| **Color** | Risk |
| green | Very low |
| white | Low |
| yellow | Moderate |
| rose | High |
| red | Very high |

**Step 3**

| HDL - Cholesterol | | |
|---|---|---|
| **(mg/dl)** | **(mmol/L)** | **Points** |
| <35 | ≤0.90 | 5 |
| 35-44 | 0.91-1.16 | 2 |
| 45-49 | 1.17-1.29 | 1 |
| 50-59 | 1.30-1.55 | 0 |
| ≥60 | ≥1.56 | -2 |

**Step 4**

| Blood Pressure | | | | | |
|---|---|---|---|---|---|
| **Systolic** | **Diastolic (mmHg)** | | | | |
| **(mmHg)** | **<80** | **80-84** | **85-89** | **90-99** | **≥100** |
| <120 | -3 pts | | | | |
| 120-129 | | 0 pts | | | |
| 130-139 | | | 0 pts | | |
| 140-159 | | | | 2 pts | |
| ≥160 | | | | | 3 pts |

Note: When systolic and diastolic pressures provide different estimates for point scores, use the higher number

**Step 5**

| Diabetes | |
|---|---|
| | **Points** |
| No | 0 |
| Yes | 4 |

**Step 6**

| Smoker | |
|---|---|
| | **Points** |
| No | 0 |
| Yes | 2 |

Risk estimates were derived from the experience of the NHLBI's Framingham Heart Study, a predominantly Caucasian population in Massachusetts, USA

**Step 7** (sum from steps 1-6)

| Adding up the points | |
|---|---|
| Age | ______ |
| LDL Cholesterol | ______ |
| HDL Cholesterol | ______ |
| Blood Pressure | ______ |
| Diabetes | ______ |
| Smoker | ______ |
| Point Total | ______ |

**Step 8** (determine CHD risk from point total)

| CHD Risk | |
|---|---|
| **Point Total** | **10 Yr CHD Risk** |
| ≤-2 | 1% |
| -1 | 2% |
| 0 | 2% |
| 1 | 2% |
| 2 | 3% |
| 3 | 3% |
| 4 | 4% |
| 5 | 5% |
| 6 | 6% |
| 7 | 7% |
| 8 | 8% |
| 9 | 9% |
| 10 | 11% |
| 11 | 13% |
| 12 | 15% |
| 13 | 17% |
| 14 | 20% |
| 15 | 24% |
| 16 | 27% |
| ≥17 | ≥32% |

**Step 9** (compare to women of the same age)

| Comparative Risk | | |
|---|---|---|
| **Age (years)** | **Average 10 Yr CHD Risk** | **Low* 10 Yr CHD Risk** |
| 30-34 | <1% | <1% |
| 35-39 | 1% | <1% |
| 40-44 | 2% | 2% |
| 45-49 | 5% | 3% |
| 50-54 | 8% | 5% |
| 55-59 | 12% | 7% |
| 60-64 | 12% | 8% |
| 65-69 | 13% | 8% |
| 70-74 | 14% | 8% |

*Low risk was calculated for a woman the same age, normal blood pressure, LDL cholesterol 100-129 mg/dL, HDL cholesterol 55 mg/dL, non-smoker, no diabetes
