# Supplementary material for: Everolimus in de novo kidney transplant recipients participating in the Eurotransplant senior program: Results of a prospective randomized multicenter study (SENATOR)
Source: PLoS One. 2019 Sep 19;14(9):e0222730. doi: 10.1371/journal.pone.0222730 (PMC6752944; doi:10.1371/journal.pone.0222730)
Supplement: S3 File — (PDF) [file pone.0222730.s003.pdf]

NOVARTIS

Clinical Development

Certican® (Everolimus)

Amendment No.2 to Protocol No. CRAD001ADE19 (SENATOR)

6-month, open-label, randomized, multicenter, prospective, controlled study to evaluate the efficacy, safety and tolerability of Everolimus in *de novo* renal transplant recipients participating in the Eurotransplant senior program

Author(s):	Dr. Eva-Maria Vogel

Document type:	Protocol Amendment
Development Phase:	Phase IIIb/IV
Protocol date:	13-May-2010
Protocol Amendment release date:	26-May-2010

Property of Novartis
Confidential
It may not be used, divulged, published or otherwise disclosed
without the consent of Novartis

Novartis approval signatures for:

Amendment No. 2 to Clinical Study Protocol CRAD001ADE19

Dr. Eva-Maria Vogel<br>Clinical Trial Leader	Vogel<br>Signature	07.07.2010<br>Date
Dr. Stefan Kramer<br>Head Clinical Research	S. Kramer<br>Signature	07.07.2010<br>Date
Dr. Christoph May<br>Trial Statistician	[illegible]<br>Signature	07-Jul-2010<br>Date
Prof. Klemens Budde<br>Co-ordinating Investigator	Signature	Date

Novartis approval signatures for:

Amendment No. 2 to Clinical Study Protocol CRAD001ADE19

Dr. Eva-Maria Vogel		
Clinical Trial Leader	Signature	Date
Dr. Stefan Kramer		
Head Clinical Research	Signature	Date
Dr. Christoph May		
Trial Statistician	Signature	Date
Prof. Klemens Budde	K. Budde	10. 6. 2010
Co-ordinating Investigator	Signature	Date

Investigator approval signatures for:

Amendment No. 2 to Clinical Study Protocol CRAD001ADE19

Investigator signature

I have read the protocol amendment and agree to conduct this trial in accordance with all stipulations of the protocol as amended, with applicable laws and regulations and in accordance with the ethical principles outlined in the Declaration of Helsinki.

K. BUDDE	K. Budde	10.6.2010
Investigator	Signature	Date

Affiliation:

Table of contents

1 Rationale for amendment ... 5
2 Changes to protocol ... 5
2.1 Section - Table of contents ... 5
2.2 Section – Glossary ... 5
2.3 Section - Protocol Synopsis ... 6
2.4 Section 1 - Background ... 6
2.5 Section 3 - Objectives ... 7
2.6 Section 4 – Study Design ... 7
2.7 Section 6.1 – Components of Investigational therapies ... 8
2.8 Section 6.2 – Treatment groups ... 8
2.9 Section 6.3 – Treatment assignment ... 8
2.10 Section 6.5.1 - Study drug administration ... 9
2.11 Section 6.5.2 - Permitted study drug adjustments ... 10
2.12 Section 6.5.5 - Study drug discontinuation ... 10
2.13 Section 6.5.6 - Premature patient withdrawal from study treatment ... 10
2.14 Section 7 - Visit schedule and assessments ... 11
2.15 Section 7.1 – Other biomarkers ... 12
2.16 Section 7.2 - Information to be collected on screening failures ... 12
2.17 Section 7.3 - Patient demographics/other baseline characteristics ... 12
2.18 Section 7.4 - Treatments ... 12
2.19 Section 7.5 – Efficacy ... 13
2.20 Section 7.6 - Safety ... 13
2.21 Section 10 – Statistical methods ... 14
2.22 Section 10.5.2 - Safety ... 15
2.23 Section 10.5.6 - Pharmacokinetics ... 15
2.24 Section 11 - Discussion and rationale for study design features ... 15
3 IRB/IEC Approval ... 15

1 Rationale for amendment

The long-term outcomes after renal transplantation are becoming the focus of scientific interest, and for a thorough evaluation of the different immunosuppressive regimens, especially for the evolution of graft function with potentially nephrotoxic Calcineurin inhibitors To further assess the long-term consequences of the different immunosuppressive protocols, it is planned that after completion of the study all randomized patients will be observed for additional 54 months.

This amendment was implemented to assess long-term effects on renal function, long-term graft and patient survival. Therefore subjects will be followed-up for up to 60 months in an observational extension period. In this extension period efficacy (biopsy-proven acute rejection, graft loss, death), renal function (creatinine, glomerular filtration rate, proteinuria) and safety (e.g. hospitalization, infection, tumor incidence, cardiovascular risk) will be assessed. Additional visits (Follow up visits) will take place at month 12, 24, 36, 48, and 60.

Finally, some inconsistencies in the protocol were corrected

2 Changes to protocol

Corrections are indicated by ***bold italic fonts*** for the addition to the text, while deleted text is indicated by, strikethrough ~~strikethrough~~.

2.1 Section - Table of contents

A ***new paragraph*** will be added to the Table of contents section:

6.5.1 Study and immunosuppressive drug administration

6.5.2 Permitted adjustments of immunosuppressive drugs

6.5.2 Discontinuation of study and immunosuppressive drug

7.1 Follow-up of Patients after End of Study

7.2 Information to be collected on screening failures and not randomized patients

2.2 Section – Glossary

The Glossary will be changed as followed:

Premature patient withdrawal	Point/time when the patient exits from the study prior to the planned completion of all study drug administration and assessments; at this time all study drug administration is discontinued and ~~no further assessments are planned~~ ***early/premature discontinuation visit (Visit 7) should be performed***

2.3 Section - Protocol Synopsis

A ***new paragraph*** will be added to the end of Secondary objectives, section Protocol synopsis:

- ***To assess efficacy (BPAR, graft loss, death), renal function (creatinine, GFR, evalution of GFR over time, proteinuria) and safety (e.g. hospitalization, infection, tumor incidence, cardiovascular risk) at follow-up visits at month 12, 24, 36, 48, and 60.***

The Study design section in the Protocol synopsis will be changed to the following and a ***new paragraph*** will be added:

Study design

Prospective, multi-center, randomized, controlled, parallel group, open-label study in de novo senior renal transplant recipients.

For the first 6 weeks post transplantation, all patients will be treated with Simulect® (induction therapy) and immunosuppressive regimen consisting of Myfortic® + Sandimmun® Optoral + corticosteroids (steroids ***should be withdrawn after*** ~~only first~~ 2 weeks)…..

Group 2: Certican® switch immunosuppressive therapy in the following steps

Step 1: Day 1 morning: Myfortic® + Sandimmun® Optoral
Day 1 evening: Myfortic® + Sandimmun® Optoral + Certican® (3 mg)

Step 2: Day 2 morning: Myfortic® + Certican® (1.5 mg)
Day 2 evening: Myfortic® + Certican® (1.5 mg)

Step 3: Day 3 morning: Myfortic® + Certican® (Trough Level [TL] 5-10 ng/mL)
Day 3 evening: Myfortic® + Certican® (Trough Level [TL] 5-10 ng/mL)

Additionally, in an observational extension period all randomized patients will be followed up for safety and graft survival for additional 4 and a half years with control assessments in yearly intervals (i.e., at Month 12, 24, 36, 48, and 60)....

.... Additionally, on day 7 ± 2 after randomization ***a control visit including*** blood level control will be performed

2.4 Section 1 - Background

The end of Background section will be added as followed:

A CNI-free regimen with Certican® and Myfortic® under the umbrella of the extended use of the IL-2R Ab Simulect will be compared with a standard Sandimmun® Optoral based immunosuppressive regimen. It is expected, that the Sandimmun® Optoral-free regimen will result in a superior renal function, while the experimental arm is as safe and effective as the Sandimmun® Optoral-based regimen with regard to occurrence of biopsy proven acute rejection episodes, graft loss, and death. ***To further assess the long-term consequences of the different immunosuppressive protocols, it is planned that after completion of the study all randomized patients will be observed for additional 48 months.*** The current study therefore aims to compare two different treatment regimens rather than individual drugs……

2.5 Section 3 - Objectives

A ***new paragraph*** will be added to the end of Secondary objectives, section 3:

- ***To assess efficacy (BPAR, graft loss, death), renal function (creatinine, GFR, evalution of GFR over time proteinuria) and safety (e.g. hospitalization, infection, tumor incidence, cardiovascular risk) at follow-up visits at month 12, 24, 36, 48, and 60.***

2.6 Section 4 – Study Design

The Study Design section will be added as followed and ***numbering of the treatment groups*** will be unified according to the numbering on page 14:

….For the first six weeks post transplantation, all study patients will receive induction therapy with Simulect® (2 x 20 mg (day 0 [2 hrs prior to Tx] and day 4 post Tx) and will commence on an immunosuppressive regimen consisting of Myfortic® "loading dose" + Sandimmun® Optoral (based on C0-h level) with corticosteroids (steroids ***should be withdrawn*** after week 2).

At BL2 (Week 7, Visit 3), patients whose eligibility is confirmed by additional in- and exclusion criteria will be randomized and hence allocated to one of the two treatment groups in a ~~2:1~~ ***1:2*** ratio.

Group ~~2~~ ***1***: Control maintain prior immunosuppressive regimen consisting of Myfortic® + Sandimmun® Optoral

Group ~~1~~ ***2***: Certican® switch immunosuppressive therapy in the following steps

Step 1: Day 1 morning: Myfortic® + Sandimmun® Optoral
Day 1 evening: Myfortic® + Sandimmun® Optoral + Certican® (3 mg)

Step 2: Day 2 morning: Myfortic® + Certican® (1.5 mg)
Day 2 evening: Myfortic® + Certican® (1.5 mg)

Step 3: Day 3 morning: Myfortic® + Certican® (TL 5 – 10 ng/mL)
Day 3 evening: Myfortic® + Certican® (TL 5 – 10 ng/mL)

In group ~~1~~ ***2***, step 1 of the therapy switch will be performed the day after randomization and will be continued on Day 2 with step 2. The therapy switch will be completed on Day 3 with step 3.

2.7 Section 6.1 – Components of Investigational therapies

The section Components of Investigational therapies will be added as followed:

Corticosteroids
according to local standard. ***The following tapering scheme for corticosteroids is recommended: 500 mg operative, 125 mg Day 1, 20 mg until Day 8, 10 mg Day 9 to Day 14.*** Steroid***s should be withdrawn*** after week 2.

2.8 Section 6.2 – Treatment groups

The ***numbering of the treatment groups*** will be unified according to the numbering on page 14:

At Baseline visit 2 (Visit 3, Week 7) patients will be assigned to one of the following two treatment groups in a ***1:2*** ~~2:1~~ ratio:

Group ~~2~~ ***1***: Control: maintain prior immunosuppressive regimen consisting of:
Sandimmun® Optoral + Myfortic®

Group ~~1~~ ***2***: Certican: switch to immunosuppressive regimen consisting of:
Certican® + Myfortic®

2.9 Section 6.3 – Treatment assignment

The section will change from the current version to the following:

At Baseline 1 which has to be performed prior to the transplantation surgery, patients will be assigned a unique patient identification by the investigator. The patient identification has two parts. The first part is the four-digit center number, which is assigned by Novartis. The second part will be assigned by the investigator starting with number “00001” in each study site. Once assigned to a patient, the patient identification will not be reused. If the patient is a screening faiure ***patient’s age, sex, ethnicity, the reason for not continuing and AEs occurred after signing the informed consent will be entered in the screening failure log.*** If the patient fails to be randomized for any reason, the patient’s identification and the reason for not being randomized will be entered on the Study Completion Page ***and Visit 1 to 3 and Visit 7, if applicable, will be entered in the CRF.*** Note: The patient identification is different from the randomization number. ....

Randomization of individual patients will be performed centrally by the designated CRO. Allocation of a patient to one of the two treatment groups will be performed in the following steps.

1. Information about patient inclusion (BL 1)

Study sites will inform the randomization department of the designated CRO about each patient inclusion via fax

~~2. Reminder (Visit 2/Week 2):~~

~~By a reminder that will be included in the CRF of the Week 2 assessments, site will be reminded to schedule the next Visit 3 (Week 7) on time~~

2~~3~~. Randomization (BL 2):

At day of randomization, the site will provide the patient information of eligible patients to the designated CRO by fax.

2.10 Section 6.5.1 - Study drug administration

The headline and the section will change from the current version to the following:

6.5.1 Study and immunosuppressive drug administration

~~One component of the box consists of a 2-part label which includes an identifier of each single box of study medication.~~ Before dispensing the study medication to an individual patient, investigator staff must enter the 4-digit patient code and the date of dispensing of the medication box ***on the box. Investigator staff also has to enter dispensing and returning of study medication on drug accounting pages.*** ~~both parts of the label. The outer part of the label will be detached from the packaging and affixed to the source document (Drug Label Form in the CRF) containing that patient's unique patient number.~~

For the other immunosuppressive drugs (~~Simulect® induction therapy~~, Sandimmun® Optoral, Myfortic®, Corticosteroids) the patient will receive a prescription.

~~Patients randomized to Certican® group will receive additional doses of~~ Simulect® (each 20 mg) ***on Day 0, Day 4 and in patients randomized to Certican® group*** on Week 7 and 12 as ~~rejection prophylaxis. These doses~~ will be provided as investigational drug……

In patients randomized to the Certican® regimen, withdrawal of Sandimmun® Optoral and commencement of Certican® will be performed in different steps as described below, starting at Visit 3 (Week 7).

Step 1: Day 1 morning: Myfortic® + Sandimmun® Optoral
Day 1 evening: Myfortic® + Sandimmun® Optoral + Certican® (3 mg)

Step 2: Day 2 morning: Myfortic® + Certican® (1.5 mg)
Day 2 evening: Myfortic® + Certican® (1.5 mg)

Step 3: Day 3 morning: Myfortic® + Certican® (TL 5 – 10 ng/mL)
Day 3 evening: Myfortic® + Certican® (TL 5 – 10 ng/mL)

….Corticosteroids will be added to the immunosuppressive regimen according to local standard but with minimum dose of 5 mg/day ***prednisolon or equivalent***. ***The following tapering scheme for corticosteroids is recommended: 500 mg operative, 125 mg Day 1, 20 mg until Day 8, 10 mg Day 9 to Day 14.*** Steroids ***should*** ~~will~~ be withdrawn after week 2 in both groups.

2.11 Section 6.5.2 - Permitted study drug adjustments

The headline and the section will change from the current version to the following:

6.5.2 – Permitted adjustments of immunosuppressive drugs

Oral corticosteroids

Oral corticosteroids will be given at a minimum dose of 5 mg prednisolon or equivalent during the first two weeks post transplantation. ***The following tapering scheme for corticosteroids is recommended: 500 mg operative, 125 mg Day 1, 20 mg until Day 8, 10 mg Day 9 to Day 14.*** If possible, it should remain stable during these two weeks. However, dose adjustments for medically indicated reasons will be possible. ***After randomization a minimum dose of 5 mg prednisolon or equivalent is allowed if in the opinion of the investigator oral corticosteroid therapy cannot be withdrawn***.

2.12 Section 6.5.5 - Study drug discontinuation

The headline will changed and ***a new paragraph*** will be added to the end of Study drug discontinuation, section 6.5.5:

6.5.5 – Discontinuation of study and immunosuppressive drug

Patients who discontinue study treatment before completing the 6-month period should be scheduled for a the end of study visit as soon as possible, at which time all of the assessments listed for the end of study visit (Visit 7, Month 6) will be performed. ***Randomized Patients who discontinue from the study prematurely will also be asked to attend the yearly safety follow-up visits (at month 12, 24, 36, 48, and 60 post transplantation).***

All patients who discontinue study drug, including those who refuse to return for a final visit, will be contacted for safety evaluations during the 30 days following the last dose of study drug taken.

2.13 Section 6.5.6 - Premature patient withdrawal from study treatment

A ***new paragraph*** will be added to the end of Premature patient withdrawal from study treatment, section 6.5.6:

All ***randomized*** patients who prematurely discontinue study treatment will not be considered as withdrawn from the study and will be asked to attend the end of study visit (at time point of

discontinuation) ***and the planned follow-up visits to obtain follow up information on renal function including evalution of GFR over time, proteinuria, rejection episodes, allograft and patient survival, hospitalizations, tumor incidence and immunosuppressive medication (starting with the first follow-up visit at month 12 for prematurely discontinued patients. This information will be captured on the follow-up CRF. Patients should be encouraged to return to the site for a required biopsy at the time of any rejection episode.***

The End of Study CRF should be completed at month 6 or earlier if the patient can no longer be followed, e.g., due to death, withdrawal of consent, or loss to follow-up.

2.14 Section 7 - Visit schedule and assessments

The section will changes as followed and a ***new section 7.1*** will be added prior to Information to be collected on screening failures, section 7.2:

Visit 7 (Week 24) – End of Treatment

In case of early discontinuation, Week 24 assessments need to be performed

- as soon as possible after treatment discontinuation
- ***as soon as possible after the patients failed to be randomized***

7.1 Follow-up of Patients after End of Study

All randomized patients who were included into this trial (i.e., who signed informed consent and received the renal allograft), whether discontinued treatment prematurely or completed the study will be followed up for safety and graft survival for additional four and a half years. Control assessments will be performed once per year, i.e. 12, 24, 36, 48, and 60 months after transplantation. Additionally, patients who discontinued prematurely for any reason before completing the 6 months study period will be asked to attend their first follow-up visit 6 months after randomization.

During the extension period, immunosuppressive regimen as taken during the study should be maintained. However, changes in the regimen based on each patient's clinical needs and the investigators' experience and discretion will be possible at any time.

During the follow-up visits (Month 12, 24, 36, 48, and 60) the following information will be obtained and recorded:

1. ***Status of patient and graft survival***
2. ***(Changes in) smoking status will be recorded and the cardiovascular risk (Framingham Score, see Appendix 7) will be calculated.***
3. ***Vital signs will be measured.***
4. ***Venous blood will be drawn for measurement of hematology and biochemistry (extended program)***
5. ***Venous blood will be drawn for measurement of Cyclosporine C0h (if Cyclosporine is taken) or Everolimus trough level (if Everolimus is taken).***
6. ***Urinalysis will be performed, the Glomerular Filtration Rate (GFR) will be assessed***

7. ***Currently taken immunosuppressive (including dosage) will be recorded***
8. ***Information on rejection episodes, renal biopsies, (severe) infections, malignancies and, hospitalization(s) that occurred since the last visit will be recorded***

2.15 Section 7.1 – Other biomarkers

The section will be added as followed:

Assessment of basiliximab concentration as well as lymphocyte immunophenotyping may be ***optional*** performed by some of the sites.

2.16 Section 7.2 - Information to be collected on screening failures

The headline and the section will be added as followed:

Section 7.2 Information to be collected on screening failures and not randomized patients

Patients who signed informed consent but in whom ***inclusion and exlusion criteria were not met or*** no transplantation was performed for any reason (“not transplanted patients”) will be recorded on the Screening Failure Log. ***Patient’s age, sex, ethnicity, the reason for not continuing and AEs occurred after signing the informed consent will be recorded.*** No further information will be obtained.

If the patient fails to be randomized into the trial after Week 7 for any reason, the patient’s number and the reason for non-randomization will be entered on the **Study Completion** ~~End of Study~~ Page. Information on CRF for Visits 1 to 3 ***and 7*** will be completed, as far as applicable and obtained.

2.17 Section 7.3 - Patient demographics/other baseline characteristics

A ***new paragraph*** will be added to Patient demographics/other baseline characteristics, section 7.3:

Relevant medical history / current medical conditions:
Relevant prior diseases and surgeries as well as concomitant diseases will be recorded with date of diagnosis / surgery and information on whether it is an active problem. The smoking status (non-smoker, ex-smoker, smoker, number of cigarettes per day) will be recorded (at BL1, BL2, and Month 6 ***as well as during follow-up visits***).

2.18 Section 7.4 - Treatments

A section will be added as followed:

Records of Simulect®, Myfortic®, Certican®, and Sandimmun® Optoral medications used and dosages administered are to be kept during the study. All changes to the medication dosing

regimen should be recorded in the Dosage Administration Record CRF, along with ***the dosage***, the reason for change and dates.

2.19 Section 7.5 – Efficacy

The section will be added as followed:

• **Rejection episodes and graft loss**
All suspected rejection episodes must be recorded on the Rejection CRF, with the date rejection was first suspected, whether a biopsy was performed, whether follow-up biopsies were performed (with subsequent dates), whether anti-rejection therapy was administered, whether the acute rejection was confirmed or with final clinical diagnosis specified, and final clinical outcome. ***Acute rejections and suspected acute rejections (e.g. increase of creatinine) are considered to be a protocol exempted event. They should not be reported simply because they result in a hospitalization and thus meet the criteria for an SAE. Acute rejections should be reported as SAEs only if they are unusual in appearance, clinical course and/or graft threatening***

<u>Biopsy-proven acute rejection</u>

....Rejection episodes will be reported on the Rejection CRF only, and not on the AE CRF, ***unless they meet the SAE criteria described above***. .....

<u>Graft loss</u>

.... The reason for graft loss will be recorded on the Graft Loss CRF. ***Graft Loss will also be reported on the AE pages of the CRF as a SAE.*** ......

2.20 Section 7.6 - Safety

Adverse events section will change from the current version to the following

<u>Adverse events</u>

An adverse event is the appearance or worsening of any undesirable sign, symptom, or medical condition occurring …….. be evaluated to determine:

1. the severity grade (mild, moderate, severe)
2. its relationship to the study drug (Certican®, Simulect® dose ***1, 2***, 3 & 4) (suspected/not suspected)
3. its duration (start and end dates or if continuing at final exam)
4. action taken i.e:
   (a) no action taken;
   (b) study drug dosage adjusted/temporarily interrupted;
   (c) study drug permanently discontinued due to this adverse event;
   (d) concomitant medication taken/adjusted/stopped
   (e) non-drug therapy given;
   (f) hospitalization/prolonged hospitalization)
5. whether it is serious, where a serious adverse event (SAE) is defined as one which:

- is fatal or life-threatening
- results in persistent or significant disability/incapacity
- constitutes a congenital anomaly/birth defect
- requires inpatient hospitalization or prolongation of existing hospitalization, unless hospitalization is for:
    - routine treatment or monitoring of the studied indication, not associated with any deterioration in condition
    - elective or pre-planned treatment for a pre-existing condition that is unrelated to the indication under study and has not worsened since the start of study drug
    - treatment on an emergency outpatient basis for an event not fulfilling any of the definitions of a SAE given above and not resulting in hospital admission
    - social reasons and respite care in the absence of any deterioration in the patient's general condition
    - ***treatment of acute rejection: acute rejections and suspected acute rejections (e.g. increase of creatinine)are considered to be a protocol exempted event. They should not be reported simply because they result in a hospitalization and thus meet the criteria for an SAE. Acute rejections should be reported as SAEs only if they are unusual in appearance, clinical course and/or graft threatening.......***

Cardiovascular risk assessment will change from the current version to the following:

Cardiovascular Risk (Framingham Score)

At BL1 (Screening), BL2 (Week 7) and at the End of Study (Month 6) ***as well as at the yearly follow-up assessments*** the patients' cardiovascular risk will be calculated using the Framingham score.

New-Onset of Diabetes Mellitus section will change from the current version to the following:

New-Onset of Diabetes Mellitus

At Baseline visit 2 and on week 24 (EOS) assessments will be performed to investigate the development of a diabetes mellitus. Therefore, fasting blood sugar level will be analyzed as well as an oral glucose tolerance test with a concomitant insulin and/or C-Peptid measurement will be performed. ***Patients with pre-existing diabetes mellitus do not require these examinations.***

2.21 Section 10 – Statistical methods

The first paragraph will be amended as follows:

The data will be analyzed by Novartis and/or by the designated CRO. Any data analysis carried out independently by the investigator(s) should be submitted to Novartis before publication or presentation. The final analysis will be done when all patients have completed the study treatment at Visit 6 (Week 24/Month 6) or discontinued prematurely. ***Data of the***

follow-up visits at Month 12, 24, 36, 48, and 60 will be analyzed and reported separately as appropriate. It is planned that the data from all centers that participate in this protocol will be used, so that an adequate number of patients will be available for analysis.

2.22 Section 10.5.2 - Safety

Cardiovascular risk calculation will change from the current version to the following:

Cardiovascular Risk (Framingham Score)

At BL1, BL2 and at Month 6, ***and at follow-up visits*** the cardiovascular risk will be calculated using the Framingham Score (see Appendix 7).

2.23 Section 10.5.6 - Pharmacokinetics

A ***new paragraph*** will be added to the Pharmacokinetics, section 10.5.6:

Analysis of the cyclosporine blood levels and Certican® (everolimus) blood levels will be done by treatment group for the treatment phase (up to Month 6) ***and separately during the follow-up phase.***

2.24 Section 11 - Discussion and rationale for study design features

A ***new paragraph*** will be added to the end of Discussion and rationale for study design features, section 11:

A maintenance treatment period of 18 weeks under both regimens is considered sufficiently long to explore any meaningful differences between the groups in the glomerular filtration rate, which is the primary efficacy outcome measure.

To assess long-term effects on renal function, randomized patients will be followed-up for up to 60 months in an extension period. Although it cannot be expected that all patients will stay on the randomized study regimen for such a long period, especially in the standard group, changes in renal function parameters can be related to those measures which are available from the first 6 month of treatment.

This has been confirmed in previous clinical trials, where CNI-withdrawal let to an immediate and significant improvement of renal function within a few weeks……

3 IRB/IEC Approval

A copy of this CRAD001ADE19 Protocol Amendment will be sent to the EC for review. The changes described in this amendment require IRB/EC approval prior to implementation.

In addition, a revised informed consent that takes into account the changes to the protocol described herein will be submitted to the IRB/EC for approval.
