# Supplementary material for: Everolimus in de novo kidney transplant recipients participating in the Eurotransplant senior program: Results of a prospective randomized multicenter study (SENATOR)
Source: PLoS One. 2019 Sep 19;14(9):e0222730. doi: 10.1371/journal.pone.0222730 (PMC6752944; doi:10.1371/journal.pone.0222730)
Supplement: S4 File — (PDF) [file pone.0222730.s004.pdf]

NOVARTIS

Clinical Development

Certican® (Everolimus)

Amendment No.3 to Protocol No. CRAD001ADE19 (SENATOR)

6-month, open-label, randomized, multicenter, prospective, controlled study to evaluate the efficacy, safety and tolerability of Everolimus in *de novo* renal transplant recipients participating in the Eurotransplant senior program

Author(s):	Dr. Eva-Maria Paulus, Dr. Christoph May
Document type:	Protocol Amendment
Development Phase:	Phase IIIb/IV
Protocol date:	13-May-2010
Protocol Amendment release date:	06-Feb-2012

Property of Novartis
Confidential
It may not be used, divulged, published or otherwise disclosed
without the consent of Novartis

Novartis approval signatures for:

Amendment No. 3 to Clinical Study Protocol CRAD001ADE19

Dr. Daniel Bäumer<br>Clinical Trial Leader	[signature]<br>Signature	15.02.2012<br>Date
Dr. Eva-Maria Paulus<br>Head Clinical Research	[signature]<br>Signature	15.02.2012<br>Date
Dr. Christoph May<br>Trial Statistician	[signature]<br>Signature	15.02.2012<br>Date
Prof. Klemens Budde<br>Co-ordinating Investigator	[signature]<br>Signature	10.2.2012<br>Date

Investigator approval signatures for:

Amendment No. 3 to Clinical Study Protocol CRAD001ADE19

Investigator signature

I have read the protocol amendment and agree to conduct this trial in accordance with all stipulations of the protocol as amended, with applicable laws and regulations and in accordance with the ethical principles outlined in the Declaration of Helsinki.

Investigator	Signature	Date

Affiliation:

Table of contents

1 Rationale for amendment 5
2 Changes to protocol 5
2.1 Changes to Section 3 Objectives 5
2.2 Changes to Section 5 Study population 5
2.3 Changes to Section 10 Data analysis 5
2.4 Changes to Section 10.7 Sample size calculation 6
3 IRB/IEC Approval 6

1 Rationale for amendment

This amendment addresses the size of study population. In the study protocol 240-260 patients were intended to be randomized. The randomization of patients is currently far beyond target. Only 74 patients could be randomized so far in a period of 30 months . It is very unlikely that the required number of patients will be achieved in a considerable amount of time. Therefore, the enrollment of patients into this study will be stopped. The analysis of the data will be performed after LPLV. The study will be analyzed in a purely exploratory manner. Thus, any results of statistical tests will be interpreted purely exploratory. Preferably, the examination of treatment differences will be based on confidence intervals.

2 Changes to protocol

Corrections are indicated by ***bold italic fonts*** for the addition to the text, while deleted text is indicated by, strikethrough ~~strikethrough~~.

2.1 Changes to Section 3 Objectives

The first paragraph has been amended as follows:

The primary objective of this trial is to ~~demonstrate~~ **exploratively examine** superiority of a treatment regimen with Certican with respect to the renal function […].

2.2 Changes to Section 5 Study population

The first paragraph has been amended as follows:

The study population will consist of a representative group of approximately 240-260 de novo senior kidney transplant patients participating at the Eurotransplant senior program (ESP).....

Due to slow recruiting it was determined that the planned sample size of 240-260 patients cannot be achieved. Thus, enrollment will be terminated with 74 randomized patients.

2.3 Changes to Section 10 Data analysis

The last paragraph was amended by adding the following sentence:

Only exploratory analyses will be performed as the trial was terminated early due to lack of obtaining the required sample size.

Changes to Section 10.4 Primary objective

The primary objective of this study is to ~~demonstrate~~ **exploratively examine** superior renal function under the Certican®-based regimen as compared to the control regimen.

Changes to Section 10.4.2 Statistical hypothesis, model, and method of analysis

This section was amended as follows

The null hypothesis of no difference in GFR at Month 6 between both treatment groups

H0: μCertican = μControl

will be tested against the two-sided alternative hypothesis of different mean GFR at Month 6 of the Certican® compared to control regimen

H1: μCertican ≠ μControl

μ indicates the mean GFR at Month 6.

The hypotheses will be tested **exploratively** with an analysis of covariance (ANCOVA) with treatment, center, and occurrence of rejections before randomization as factors, and GFR at Visit 3 (Baseline 2) as covariate. Raw as well as adjusted means (= LS-means, LS: least square means) will be presented for the treatment contrast together with its confidence interval and a two-sided p-value. The significance level will be 5% (two-sided). The full analysis set (FAS) will be used for the primary analysis. There are no multiplicity issues to be addressed since only the hypothesis of superior efficacy of the Certican® vs. the control regimen will be tested confirmatorily.

2.4 Changes to Section 10.7 Sample size calculation

The section was amended by adding the following sentence:

From data for the subgroup of elderly patients in study CRAD001A2418, the probable difference in the GFR (Cockcroft-Gault) between the CNI-free regimen group and the standard regimen group is estimated as δ = 8 ml/min with a standard deviation of 16 ml/min at Month 6 after transplantation. Assuming a difference in the mean GFR of 8 ml/min (60 ml/min in the CNI-free group and 52 ml/min in the standard regimen group) and a common standard deviation of 19 ml/min, a two-group t-test with a 5% two-sided significance level will have 80% power to detect this difference when the sample sizes in the CNI-free group is 135 and in the standard regimen group is 68, respectively (a total sample size of 203). Taking into account a common drop-out rate of 10%, 162 patients need to be randomized into the CNI-free group, and 82 into the standard regimen group (a total drop-out adjusted sample size of 244 patients). Enrollment will be continued until the required sample size will be achieved. **Due to slow recruiting it was determined that the planned sample size of 240-260 patients cannot be achieved. Thus, enrollment will be terminated with 74 randomized patients.**

3 IRB/IEC Approval

A copy of this CRAD001ADE19 Protocol Amendment will be sent to the EC for review. The changes described in this amendment require IRB/EC approval prior to implementation.
