# Supplementary material for: Everolimus in de novo kidney transplant recipients participating in the Eurotransplant senior program: Results of a prospective randomized multicenter study (SENATOR)
Source: PLoS One. 2019 Sep 19;14(9):e0222730. doi: 10.1371/journal.pone.0222730 (PMC6752944; doi:10.1371/journal.pone.0222730)
Supplement: S5 File — (PDF) [file pone.0222730.s005.pdf]

NOVARTIS

Clinical Development

Certican® (Everolimus)

Amendment No.4 to CRAD001ADE19 (SENATOR)

6-month, open-label, randomized, multicenter, prospective, controlled study to evaluate the efficacy, safety and tolerability of Everolimus in *de novo* renal transplant recipients participating in the Eurotransplant senior program

Author(s): Dr. Daniel Bäumer, Dr. Celia Antonio Schell

Document type: Amendment

Development Phase: Phase IIIb/IV

Protocol date: 08-Feb-2009

Amendment date: 06-Nov-2012

Property of Novartis
Confidential
It may not be used, divulged, published or otherwise disclosed
without the consent of Novartis

Novartis approval signatures for:

Amendment No. 4 to Clinical Study CRAD001ADE19

Dr. Daniel Bäumer<br>Clinical Trial Leader	[signature]<br>Signature	28.11.2012<br>Date
Dr. Eva-Maria Paulus<br>Head Clinical Research	Paulus<br>Signature	29.11.2012<br>Date
Christian Sieder<br>Trial Statistician	[signature]<br>Signature	28NOV2012<br>Date
Prof. Klemens Budde<br>Co-ordinating Investigator	K. Budde<br>Signature	20.11.2012<br>Date

CHARITÉ
UNIVERSITÄTSMEDIZIN BERLIN
Klinik mit Schwerpunkt Nephrologie
Prof. Dr. med. Klemens Budde
Campus Charité Mitte [illegible]
Charitéplatz 1 | D-10117 [illegible]
Telefon: (030) 450 - 5[illegible]
Telefax: (030) 450 - 514 [illegible]

Investigator approval signatures for:

Amendment No. 4 to Clinical Study CRAD001ADE19

Investigator signature

I have read the protocol amendment and agree to conduct this trial in accordance with all stipulations of the protocol as amended, with applicable laws and regulations and in accordance with the ethical principles outlined in the Declaration of Helsinki.

Investigator	Signature	Date

Affiliation:

Table of contents

1 Rationale for amendment 5
2 Follow-up End of Treatment Visit 5
3 IRB/IEC..6

1 Rationale for amendment

This amendment addresses the end of the study CRAD001ADE19 in the Follow up Phase at month 60.

In the original approved study protocol (v 0.1 release date 08-02-2009) 240-260 patients were intended to be randomized. Due to a very slow enrollment, randomization of patients has been far beyond target. Only 74 patients could be randomized in a period of 30 months, therefore enrollment of patients into this study was stopped (protocol amendment 3) and the last patient was randomized in May 2012.

The sample size in the CNI-free group has been originally estimated to a required number of 162 randomized patients. Actually only 26 of 74 randomized patients are on study drug (Everolimus) in the CNI-free arm of the study. Due to the low number of patients in the Everolimus arm, the long-term follow-up will be stopped. An “End of study” visit will be conducted for all patients to obtain final data and to inform the patients of the study termination.

2 Follow-up End of Treatment Visit

All patients that have completed the core study and are currently in the follow-up period will be asked to attend a final end of study visit (End of Follow-up visit) as soon as possible and no later than end of 28.02.2013. This visit will be conducted for all patients currently in follow-up even if they e.g. already attended month visit 24 follow up visit.

During the End of Follow-up visit the patients will be informed that Study CRAD001ADE19 will be stopped. In addition, the following information will be obtained and recorded during the visit:

	End of Follow-up visit
Status of patient and graft survival	x
Information on rejection episodes, renal biopsies, (severe) infections, malignancies and hospitalizations	x
General medical history<br>-graft survival<br>-smoking status	x
Diabetes Status	x
Physical examination	x

Vital signs	x
Currently taken immunosuppression[1]<br>-drug concentration trough level[2]	x
Laboratory test-Haematology/Biochemistry[3]	x
Urinanalysis/GFR[4]	x

1) Currently taken immunosuppressive (including dosage) will be recorded
2) Venous blood will be drawn for measurement of Cyclosporine C0h
   (if Cyclosporine is taken) or Everolimus trough level (if Everolimus is taken).
3) Venous blood will be drawn for measurement of hematology
4) Urinalysis will be performed, the Glomerular Filtration Rate (GFR) will be assessed

3 IRB/IEC

A copy of this CRAD001ADE19 Protocol Amendment will be sent to the EC for review. The changes described in this amendment require IRB/EC approval prior to implementation.
